# The impact of rare protein coding genetic variation on adult cognitive function

**DOI:** 10.1038/s41588-023-01398-8

**Published:** 2023-05-25

**Authors:** Chia-Yen Chen, Ruoyu Tian, Tian Ge, Max Lam, Gabriela Sanchez-Andrade, Tarjinder Singh, Lea Urpa, Jimmy Z. Liu, Mark Sanderson, Christine Rowley, Holly Ironfield, Terry Fang, Biogen Biobank Team, The SUPER-Finland study, Aija Kyttälä, Amanda Elliott, Anders Kämpe, Andre Sourander, Annamari Tuulio-Henriksson, Anssi Solismaa, Antti Tanskanen, Ari Ahola-Olli, Arto Mustonen, Arttu Honkasalo, Asko Wegelius, Atiqul Mazumder, Auli Toivola, Benjamin Neale, Elina Hietala, Elmo Saarentaus, Erik Cederlöf, Erkki Isometsä, Heidi Taipale, Imre Västrik, Jaana Suvisaari, Jari Tiihonen, Jarmo Hietala, Johan Ahti, Jonne Lintunen, Jouko Lönnqvist, Juha Veijola, Julia Moghadampour, Jussi Niemi-Pynttäri, Kaisla Lahdensuo, Katja Häkkinen, Katriina Hakakari, Kimmo Suokas, Marjo Taivalantti, Markku Lähteenvuo, Martta Kerkelä, Minna Holm, Nina Lindberg, Noora Ristiluoma, Olli Kampman, Olli Pietiläinen, Risto Kajanne, Sari Lång-Tonteri, Solja Niemelä, Steven E. Hyman, Susanna Rask, Teemu Männynsalo, Tiina Paunio, Tuomas Jukuri, Tuomo Kiiskinen, Tuula Kieseppä, Ville Mäkipelto, Willehard Haaki, Zuzanna Misiewicz, The Northern Finland Intellectual Disability study, Mitja I. Kurki, Jarmo Körkkö, Jukka Moilanen, Outi Kuismin, Mark Daly, Aarno Palotie, Ellen A. Tsai, Hailiang Huang, Matthew E. Hurles, Sebastian S. Gerety, Todd Lencz, Heiko Runz

**Affiliations:** 1 Research and Development, Biogen Inc, Cambridge, MA USA; 2 Psychiatric and Neurodevelopmental Genetics Unit, Center for Genomic Medicine, Massachusetts General Hospital, Boston, MA USA; 3 Department of Psychiatry, Massachusetts General Hospital, Harvard Medical School, Boston, MA USA; 4 Stanley Center for Psychiatric Research, Broad Institute of MIT and Harvard, Cambridge, MA USA; 5 Division of Psychiatry Research, The Zucker Hillside Hospital, Northwell Health, Glen Oaks, NY USA; 6 Institute of Behavioral Science, Feinstein Institutes for Medical Research, Manhasset, NY USA; 7 Wellcome Sanger Institute, Cambridge, UK; 8 Analytic and Translational Genetics Unit, Massachusetts General Hospital, Boston, MA USA; 9 Institute for Molecular Medicine Finland, HiLIFE, University of Helsinki, Helsinki, Finland; 10 Program in Medical and Population Genetics, Broad Institute of MIT and Harvard, Cambridge, MA USA; 11 Department of Medicine, Harvard Medical School, Boston, MA USA; 12 Department of Psychiatry, Zucker School of Medicine at Hofstra/Northwell, Hempstead, NY USA; 13 Department of Molecular Medicine, Zucker School of Medicine at Hofstra/Northwell, Hempstead, NY USA; 14 Finnish Institute for Health and Welfare, Helsinki, Finland; 15 Department of Child Psychiatry, University of Turku, Turku, Finland; 16 Department of Psychology and Logopedics, Faculty of Medicine, University of Helsinki, Helsinki, Finland; 17 Tampere University and Tampere University Hospital, Tampere, Finland; 18 Niuvanniemi Hospital, University of Eastern Finland, Kuopio, Finland; 19 Department of Clinical Neuroscience, Karolinska Institutet, Stockholm, Sweden; 20 Impact Assessment Unit, Finnish Institute for Health and Welfare, Helsinki, Finland; 21 University of Turku, Turku, Finland; 22 University of Helsinki, Helsinki, Finland; 23 Department of Psychiatry, University of Helsinki and Helsinki University Hospital, Helsinki, Finland; 24 Unit of Clinical Neuroscience, Faculty of Medicine, University of Oulu, Oulu, Finland; 25 School of Pharmacy, University of Eastern Finland, Kuopio, Finland; 26 Mental Health Unit, Finnish Institute for Health and Welfare, Helsinki, Finland; 27 Department of Forensic Psychiatry, Niuvanniemi Hospital, University of Eastern Finland, Kuopio, Finland; 28 Department of Psychiatry, Turku University Hospital, Turku, Finland; 29 Department of Psychiatry, Research Unit of Clinical Neuroscience, University of Oulu, Oulu, Finland; 30 Department of Psychiatry, University Hospital of Oulu, Oulu, Finland; 31 Mehiläinen, Helsinki, Finland; 32 Hospital District of Helsinki and Uusimaa, Helsinki, Finland; 33 Department of Psychiatry, Pirkanmaa Hospital District, Tampere, Finland; 34 Research Unit of Clinical Neuroscience, University of Oulu, Oulu, Finland; 35 Neuroscience Center, HiLIFE, University of Helsinki, Helsinki, Finland; 36 Department of Psychiatry, University of Turku, Turku, Finland; 37 Center for Intellectual Disability Care, Oulu University Hospital, Oulu, Finland; 38 Department of Clinical Genetics, Research Unit of Clinical Medicine, Medical Research Center Oulu, Oulu University Hospital and University of Oulu, Oulu, Finland; 39 Present Address: Dewpoint Therapeutics, Boston, MA USA; 40 Present Address: GlaxoSmithKline, Philadelphia, PA USA

**Keywords:** Genetics research, Genetic association study, Neurodevelopmental disorders

## Abstract

Compelling evidence suggests that human cognitive function is strongly influenced by genetics. Here, we conduct a large-scale exome study to examine whether rare protein-coding variants impact cognitive function in the adult population (*n* = 485,930). We identify eight genes (*ADGRB2*, *KDM5B*, *GIGYF1*, *ANKRD12*, *SLC8A1*, *RC3H2*, *CACNA1A* and *BCAS3*) that are associated with adult cognitive function through rare coding variants with large effects. Rare genetic architecture for cognitive function partially overlaps with that of neurodevelopmental disorders. In the case of *KDM5B* we show how the genetic dosage of one of these genes may determine the variability of cognitive, behavioral and molecular traits in mice and humans. We further provide evidence that rare and common variants overlap in association signals and contribute additively to cognitive function. Our study introduces the relevance of rare coding variants for cognitive function and unveils high-impact monogenic contributions to how cognitive function is distributed in the normal adult population.

## Abstract (web summary)

Analysis of rare coding variants in the UK Biobank identifies eight genes associated with adult cognitive function, including *KDM5B*. Rare and common variant signals overlap and contribute additively to the phenotype.

## Main

Cognitive function is a complex trait consisting of mental processes that include attention, memory, processing speed, spatial ability, language and problem-solving^1–4^. General cognitive function and specific cognitive domains can be reliably measured across individuals in the human population and throughout the life span^2^. Cognitive function in adults, as ascertained either directly via cognitive tests or using proxy measures such as educational attainment (EDU), is strongly influenced by genetics and shows substantial genetic correlation with physical and mental health outcomes as well as mortality^1^. Nearly 4,000 cognitive function loci of individually small effect sizes have been identified through common variant-based genome-wide association studies (GWAS)^2–6^. GWAS have also demonstrated shared genetic contributions between cognitive function and neurodevelopmental disorders^7–10^, for which large-scale exome studies have identified hundreds of underlying genes^7,11–13^. However, beyond a proposed deleterious effect of exome-wide rare protein-truncating variant (PTV) burden^14,15^, no studies have yet systematically interrogated the impact of rare coding variants on cognitive phenotypes in the adult general population.

To advance gene discovery for cognitive phenotypes beyond GWAS and gain deeper insights into the shared genetic components between adult cognitive function and neurodevelopmental disorders, we analyzed exome sequencing and genome-wide genotyping data from 454,787 UK Biobank (UKB) participants with measures of cognitive function. We show that adult cognitive function is strongly influenced by the exome-wide burden of rare protein-coding variants and identify and replicate eight genes that are associated with adult cognitive phenotypes. For one of these cognitive function genes, *KDM5B*, we demonstrate in mice and humans that reduced cognitive function at the population level can be part of a phenotypic spectrum in which cognitive performance depends on the genetic dose of a single gene. Finally, our study bridges a gap between common complex trait and rare disease genetics by demonstrating that adult cognitive function is influenced by additive effects between rare and common variant-based polygenic risk that can be traced to overlapping genomic loci and biological pathways.

## Results

The UKB is a prospective cohort study of over 500,000 participants with extensive health and lifestyle data and genome-wide genotyping and sequencing^16–22^. We chose to study the genetic basis of three distinct, yet interrelated phenotypes that previous studies used to approximate adult cognitive function: educational attainment (EDU); reaction time (RT); and verbal-numerical reasoning (VNR)^23^. EDU is derived from a survey regarding years of schooling, which is genetically correlated with both adult (*r*_*g*_ = 0.66) and childhood cognitive function (*r*_*g*_ = 0.72) (refs. ^5,24,25^). RT is based on a digital test that measures processing speed, a component of general cognitive function^26,27^. VNR is a measure of general cognitive function based on questionnaires. We annotated exome sequencing data from 454,787 UKB participants^18,19^ for PTVs, missense variants and synonymous variants^28,29^ and identified rare coding variants with a minor allele frequency (MAF) < 10^−5^ in the UKB, following previous exome studies on cognition-related traits^12,14,15,21,30^. We further annotated all variants according to gene intolerance to loss-of-function (LoF) and missense variants for deleteriousness^31^. In total, we analyzed 649,321 protein-truncating, 5,431,793 missense and 3,060,387 synonymous rare variants.

### Rare variants influence adult cognitive function

**Fig. 1:**
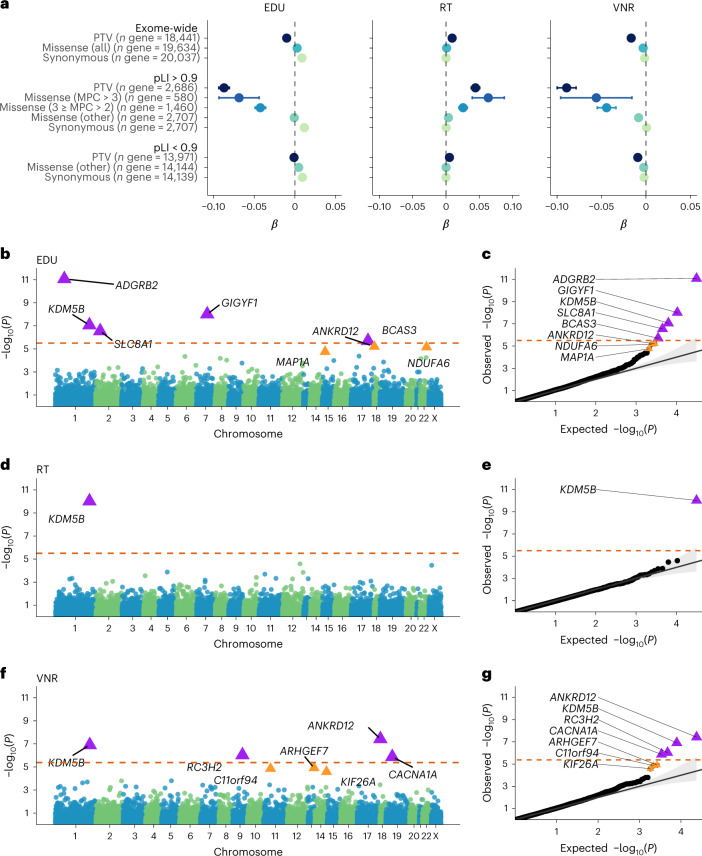
Impact of exome-wide burden of rare protein-coding variants and gene discovery based on the PTV burden for EDU, RT and VNR in EUR samples in the UKB. **a**, The effects of protein-truncating, missense (stratified by MPC) and synonymous variant burden on EDU, RT and VNR across the exome and stratified by genes intolerant (pLI ≥ 0.9) or tolerant (pLI < 0.9) to PTVs. Unrelated UKB EUR samples were included in this analysis (*n* = 318,844 for EDU, *n* = 319,536 for RT and *n* = 128,812 for VNR). pLI is the probability of being LOF-intolerant as recorded in the gnomAD database. Missense variants were classified according to deleteriousness (MPC) into three tiers: MPC > 3; 3 ≥ MPC > 2; and other missense variants not in the previous two tiers. The number of genes included in each burden was labeled. Data are presented as effect size estimates (*β*) with 95% confidence intervals (CIs). **b**, Exome-wide, gene-based PTV burden association for EDU (related UKB EUR sample *n* = 393,758). The −log_10_ *P* values (two-sided *t*-test) for each gene were plotted against the genomic position (Manhattan plot). The orange dashed line indicates the Bonferroni-corrected exome-wide significance level per phenotype (*P* < 0.05/15,782 = 3.17 × 10^−6^ for EDU). The purple triangles indicate Bonferroni-significant genes. The orange triangles indicate FDR-significant genes (FDR *Q* < 0.05). **c**, Observed −log_10_ *P* value (two-sided *t*-test) plotted against expected values (Q–Q plot) for exome-wide, gene-based PTV burden association for EDU. The orange dashed line indicates the Bonferroni-corrected exome-wide significance level per phenotype (*P* < 0.05/15,782 = 3.17 × 10^−6^ for EDU). **d**, Exome-wide, gene-based PTV burden association for RT (related UKB EUR sample *n* = 394,600). The −log_10_ *P* values (two-sided *t*-test) for each gene were plotted against the genomic position (Manhattan plot). The orange dashed line indicates the Bonferroni-corrected exome-wide significance level per phenotype (*P* < 0.05/15,798 = 3.16 × 10^−6^ for RT). **e**, Observed −log_10_ *P* value (two-sided *t*-test) plotted against the expected values (Q–Q plot) for exome-wide, gene-based PTV burden association for RT. The orange dashed line indicates the Bonferroni-corrected exome-wide significance level per phenotype (*P* < 0.05/15,798 = 3.16 × 10^−6^ for RT). **f**, Exome-wide, gene-based PTV burden association for VNR (related UKB EUR sample *n* = 159,026). The −log_10_ *P* values (two-sided *t*-test) for each gene were plotted against the genomic position (Manhattan plot). The orange dashed line indicates the Bonferroni-corrected exome-wide significance level per phenotype (*P* < 0.05/11,905 = 4.20 × 10^−6^ for VNR). **g**, Observed −log_10_ *P* value (two-sided *t*-test) plotted against the expected values (Q–Q plot) for exome-wide, gene-based PTV burden association for VNR. The orange dashed line indicates the Bonferroni-corrected exome-wide significance level per phenotype (*P* < 0.05/11,905 = 4.20 × 10^−6^ for VNR).

We first examined the impact of rare coding variant burden on EDU, RT and VNR in unrelated UKB participants of European (EUR) ancestry (*n* = 321,843; Fig. 1a and Supplementary Tables 1 and 2). We showed that exome-wide PTV and missense burden have significant deleterious effects on cognitive function, which is reflected in lower EDU, longer RT and lower VNR scores per variant count (exome-wide PTV burden: *P* = 1.95 × 10^−21^ for EDU, 8.79 × 10^−19^ for RT and 6.99 × 10^−22^ for VNR; missense burden: *P* = 5.95 × 10^−24^ for EDU, 5.95 × 10^−4^ for RT and 4.87 × 10^−12^ for VNR). Consistent with previous exome studies^12–15,30^, the most pronounced signals were driven by PTVs and damaging missense variants (missense badness, PolyPhen-2, and constraint (MPC) > 3 and 3 ≥ MPC > 2) in LoF-intolerant genes (pLI ≥ 0.9) (refs. ^32,33^). The effect sizes of PTV and the MPC > 3 missense burden in LoF-intolerant genes were not significantly different (Fig. 1), suggesting that both classes of variants may impact cognitive function similarly. The synonymous variant burden showed an inverse, albeit small, effect on EDU (exome-wide *β* = 0.0087, *P* = 8.59 × 10^−75^), but not on RT and VNR.

After the exome-wide burden analyses, we performed gene-based PTV burden tests to identify genes associated with EDU, RT and VNR using two-step whole-genome regression implemented in regenie^34^. By analyzing 397,434 EUR samples in the UKB, we identified eight genes associated with one or more cognitive phenotypes at exome-wide significance after Bonferroni correction (Table 1 and Fig. 1b–g). These cognitive function genes included *KDM5B* (for all three phenotypes), *ADGRB2*, *GIGYF1*, *SLC8A1*, *BCAS3* (for EDU), *ANKRD12* (for VNR and for EDU with false discovery rate (FDR) significance), *RC3H2* and *CACNA1A* (for VNR). As expected, PTV burden in these eight genes showed deleterious effects on cognitive function^14,15^. We also identified five putative cognitive function genes at an FDR of *Q* < 5% (*NDUFA6*, *ARHGEF7*, *C11orf94*, *KIF26A* and *MAP1A*; Supplementary Tables 3–5). In addition to the EUR samples, we also examined the impact of rare coding variants in UKB participants of South Asian (SAS) (*n* = 9,224) and African (AFR) (*n* = 8,406) ancestries; Supplementary Tables 4–7 and Supplementary Figs. 1 and 2). However, analyses in non-EUR samples were underpowered to replicate our findings in UKB EUR samples.

**Table 1:** Exome-wide, gene-based, PTV burden association-identified genes for EDU, RT and VNR in EUR samples in the UKB

Gene symbol	Associated phenotype(s)	EDU			RT			VNR			Known gene–phenotype relationships
		*β* (95% CI)	*P*	*n* PTV carrier	*β* (95% CI)	*P*	*n* PTV carrier	*β* (95% CI)	*P*	*n* PTV carrier	
*ADGRB2*	EDU^a^	−0.664 (−0.854 to −0.473)	**8.55** **×** **10**^**−12**^	71	0.159 (−0.070 to 0.388)	0.174	70	−0.615 (−0.976 to −0.255)	8.28 × 10^−4^	25	
*KDM5B*	EDU^a^/RT^a^/VNR^a^	−0.307 (−0.419 to −0.195)	**8.68** **×** **10**^**−8**^	204	0.447 (0.311 to 0.582)	**9.60** **×** **10**^**−11**^	201	−0.547 (−0.750 to −0.344)	**1.24** **×** **10**^**−7**^	79	MIM: autosomal recessive intellectual developmental disorder-65
											Exome study: DD, ASD
*GIGYF1*	EDU^a^	−0.492 (−0.660 to −0.324)	**9.80** **×** **10**^**−9**^	91	0.275 (0.076 to 0.473)	6.78 × 10^−3^	93	−0.490 (−0.771 to −0.209)	6.43 × 10^−4^	41	Exome study: DD, ASD
*ANKRD12*	VNR^a^/EDU^b^	−0.310 (−0.445 to −0.176)	6.26 × 10^−6^	142	0.101 (−0.057 to 0.260)	0.210	146	−0.694 (−0.941 to −0.447)	**3.77** **×** **10**^**−8**^	53	Exome study: SCZ
*SLC8A1*	EDU^a^	−0.992 (−1.371 to −0.613)	**2.84** **×** **10**^**−7**^	18	0.202 (−0.250 to 0.654)	0.381	18	NA	NA	4	
*RC3H2*	VNR^a^	−0.337 (−0.612 to −0.062)	0.016	34	0.132 (−0.196 to 0.461)	0.430	34	−1.126 (−1.576 to −0.676)	**9.32** **×** **10**^**−7**^	16	
*CACNA1A*	VNR^a^	−0.210 (−0.391 to −0.029)	0.023	78	0.352 (0.129 to 0.575)	1.95 × 10^−3^	74	−0.824 (−1.159 to −0.490)	**1.33** **×** **10**^**−6**^	29	MIM: spinocerebellar ataxia-6; type 2 episodic ataxia; familial hemiplegic migraine-1; developmental and epileptic encephalopathy-42. Exome study: DD
*BCAS3*	EDU	−0.419 (−0.592 to −0.246)	**1.99** **×** **10**^**−6**^	86	0.207 (−0.003 to 0.417)	0.054	83	−0.361 (−0.670 to −0.053)	0.022	34	MIM: Hengel–Maroofian–Schols syndrome

The sample sizes, number of genes tested and *λ*_GC_ for each phenotype are as follows: *n*_sample_ = 393,758, *n*_test_ = 15,782 and *λ*_GC_ = 0.967 for EDU; *n*_sample_ = 394,600, *n*_test_ = 15,798 and *λ*_GC_ = 0.961 for RT; and *n*_sample_ = 159,026, *n*_test_ = 11,905 and *λ*_GC_ = 0.959 for VNR. We excluded genes with fewer than ten PTV carriers from the analysis. The ‘associated phenotype(s)’ column indicates the phenotype for each gene with Bonferroni significance (adjusted by *n*_test_ for each phenotype). ^a^Indicates genes that showed exome-wide significant association (bold) after Bonferroni correction across all tests (two-sided *t*-test: *P* < 0.05/43,485 = 1.15 × 10^−6^). ^b^FDR was significant for EDU. *β* values represent rank-based inverse-normal transformed phenotypes and correspond to s.d. change in the phenotype. The table was sorted according to the lowest *P* value across three phenotypes.

We next aimed to replicate our findings in three independent EUR cohorts: the SUPER-Finland study (9,883 cases with psychosis); Northern Finland Intellectual Disability (NFID) study (1,097 cases with intellectual disability (ID), 11,774 controls)^35^; and Mass General Brigham Biobank (MGBB) (8,389 population cohort), for which exome sequencing and cognitive function phenotypes were available. We performed association analyses on an aggregated gene set of all eight cognitive function genes identified in the UKB against developmental disorders (DDs)/ID (SUPER-Finland and NFID studies), academic performance (SUPER-Finland study) and EDU (SUPER-Finland study and MGBB). Consistent with our findings in the UKB, PTV burden was associated with lower EDU (*β* = −0.424, *P* = 0.0021), lower academic performance (*β* = −0.338, *P* = 0.0125) and higher risk for DD/ID (odds ratio (OR) = 4.812, *P* = 8.30 × 10^−4^) in the SUPER-Finland study (Supplementary Table 8). The association between the cognitive function gene set and cognitive function in the SUPER-Finland study was conditioned on all samples from this cohort being cases with psychosis, which suggests that the observed effects on cognitive function were independent from and in addition to the potential effects of psychosis. In the NFID study, PTV burden in the cognitive function gene set was also associated with higher risk for DD/ID (OR = 4.973, *P* = 3.63 × 10^−5^). The MGBB data showed concordant results in the general population (*β* = −0.731, *P* = 0.5013 for EDU). Meta-analyses across replication cohorts for DD/ID (SUPER-Finland and NFID studies) showed lower association *P* values (*P* = 1.57 × 10^−8^; Supplementary Table 8) than the individual studies. Replication analyses for individual genes yielded supportive results but did not reach statistical significance due to the much smaller replication sample sizes than those in the UKB. Overall, our replication analyses validated that LoF in the cognitive function genes identified in the UKB reduces adult cognitive function.

**Extended Data Fig. 1:**
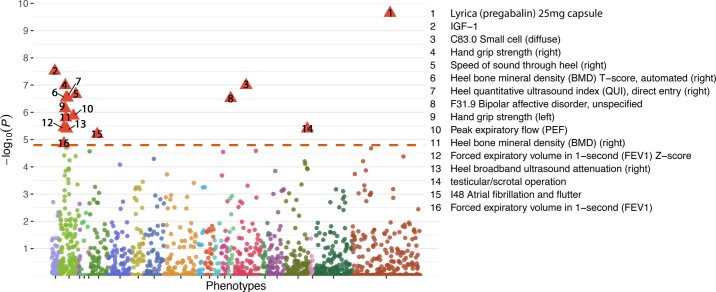
PTV burden-based phenome-wide association analysis (3,150 phenotypes) for *KDM5B* in the UK Biobank unrelated European samples (N = 321,843). FEV1 Z-score is Inverted GLI 2012 z-score for FEV1. Phenotypes were grouped and color-coded from left to right in the following categories: biomarker; composite phenotypes; family history; ICD-10 cause of death, ICD-10 congenital malformations; deformations and chromosomal abnormalities; ICD-10 diseases of the circulatory system; ICD-10 diseases of the digestive system; ICD-10 diseases of the eye and adnexa; ICD-10 diseases of the genitourinary system; ICD-10 diseases of the musculoskeletal system and connective tissue; ICD-10 diseases of the nervous system; ICD-10 diseases of the respiratory system; ICD-10 diseases of the skin and subcutaneous tissue; ICD-10 endocrine, nutritional and metabolic diseases; ICD-10 mental, behavioral and neurodevelopmental disorders; ICD-10 neoplasms; ICD-10 pregnancy, childbirth and the puerperium; ICD-10 symptoms, signs and abnormal clinical and laboratory findings, not elsewhere classified; operation code; self-reported illness: cancer; self-reported illness: non−cancer; self-reported medication. Bonferroni corrected p-value (two-sided *t*-test) significance threshold was 0.05/3150 = 1.59×10^−5^.

To systematically assess whether the LoF of the eight cognitive genes also impacted phenotypes beyond cognitive function, we conducted PTV burden-based phenome-wide association studies (PheWAS) with 3,150 phenotypes in unrelated UKB EUR samples. Indeed, PheWAS suggested pleiotropy for six of the eight cognitive function genes. For instance, a rare PTV burden in *KDM5B* was not only strongly associated with all three cognitive function phenotypes studied (*β* = −0.307, *P* = 8.68 × 10^−8^ for EDU; *β* = 0.447, *P* = 9.60 × 10^−11^ for RT; *β* = −0.547, *P* = 1.24 × 10^−7^ for VNR), but also showed 16 additional phenome-wide significant associations related to muscle function (for example, hand grip strength (right), *P* = 1.02 × 10^−7^), skeletal phenotypes (for example, heel bone mineral density T-score, automated (right), *P* = 2.93 × 10^−7^), bipolar disorder (BD) (*P* = 3.04 × 10^−7^) and pain medication use (pregabalin, *P* = 2.27 × 10^−10^), among others (Extended Data Fig. 1 and Supplementary Table 9). Similarly, the PheWAS for *ANKRD12* identified 11 phenome-wide significant associations including dysarthria and anarthria (motor disorders with speech deficit; International Classification of Diseases 10th Revision (ICD-10) code R47.1; *P* = 2.28 × 10^−9^), which suggests a potential mechanism of how *ANKRD12* might affect VNR and EDU. Other notable phenome-wide-significant associations include type 2 diabetes and related phenotypes for *GIGYF1* (refs. ^36,37^), chlorpromazine (antipsychotic) use and impaired cognitive function and awareness (ICD-10 code R41.8) for *ADGRB2* (Supplementary Fig. 3 and Supplementary Table 9). The substantial pleiotropy indicates that these genes do not impact cognitive function in isolation. To provide insights into the potential mechanisms, we curated known and proposed medical and biological roles for all genes identified in our study (Table 1, Supplementary Table 7 and Supplementary Information).

### Cognition and neurodevelopmental genes overlap

**Fig. 2:**
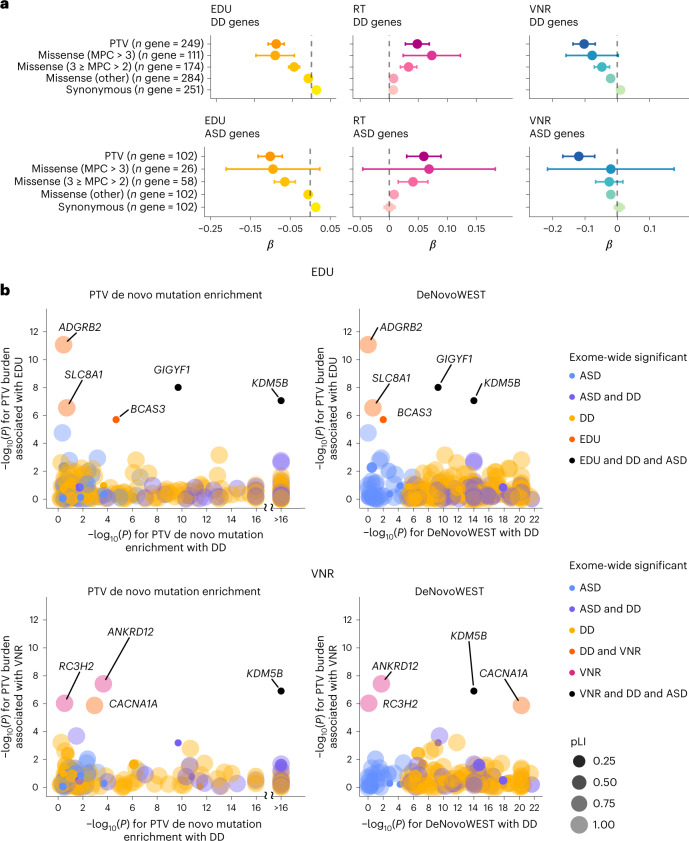
Impact of rare coding variants on cognitive function in DD and ASD genes. **a**, The effects of protein-truncating, missense (stratified by MPC) and synonymous variant burden in the exome sequencing study identified DD^13^ and ASD genes^12^ on EDU, RT and VNR. Unrelated UKB EUR samples were included in this analysis (*n* = 318,844 for EDU, *n* = 319,536 for RT and *n* = 128,812 for VNR). Missense variants were classified according to deleteriousness (MPC) into three tiers: tier 1, MPC > 3; tier 2, 3 ≥ MPC > 2; tier 3 includes all missense variants not in tier 1 or 2. Data are presented as effect size estimates (*β*) with 95% CIs. **b**, Comparison between gene-based associations for DD, EDU and VNR (PTV de novo mutation enrichment tests (simulation-based test) and DeNovoWEST (simulation-based test) for DD; rare PTV burden association tests (two-sided *t*-test) for EDU and VNR). Each dot represents a gene that was identified for DD in Kaplanis et al.^13^ or for EDU or VNR in the current exome analysis. The dots are color-coded according to the phenotypes (DD, ASD, EDU and VNR) that the gene is significantly associated with exome-wide. The size and shade of the dots represent the pLI for the gene. EDU and VNR genes are labeled with gene names.

Sequencing has identified hundreds of genes underlying DDs and autism spectrum disorder (ASD) that both diseases partially share^12,13^. As some of the genes we identified are known to cause Mendelian DDs (Table 1), we aimed to elucidate the overall rare genetic variation overlap between adult cognitive function, DDs and ASD. We tested whether the rare coding variant burdens in 285 DD-associated genes and 102 ASD-associated genes are associated with adult cognitive function. We observed significant deleterious effects of PTV burden in DD and ASD genes on all three cognitive phenotypes analyzed (Fig. 2a and Supplementary Table 10), while damaging missense variants (MPC > 3 or 3 ≥ MPC > 2) also showed similar deleterious effects.

**Extended Data Fig. 2:**
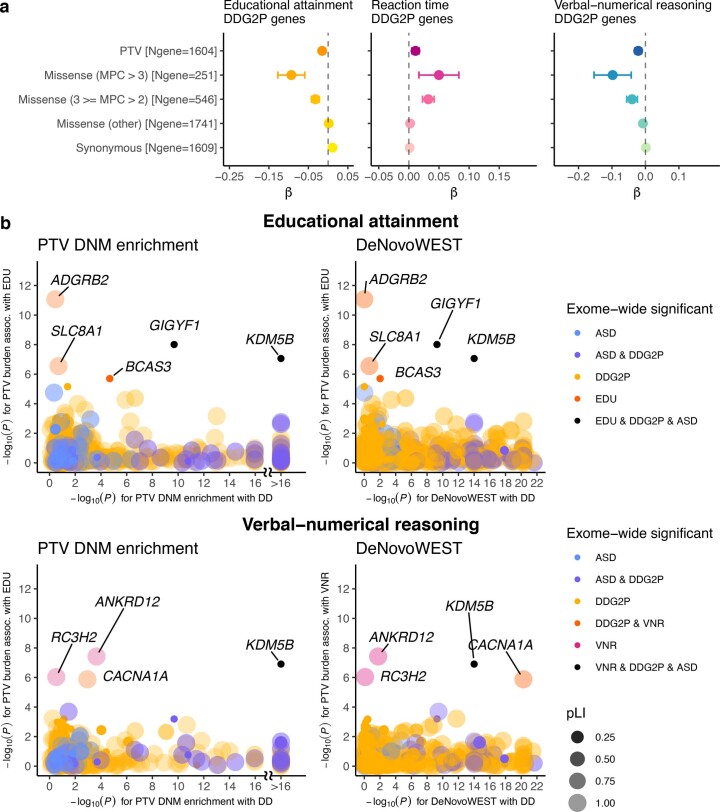
Impact of rare coding variants in genes identified in the Developmental Disorder Genotype - Phenotype Database (DDG2P) on cognitive function. a. The effects of protein-truncating, missense (stratified by MPC) and synonymous variant burden in exome sequencing study identified DDG2P on EDU, RT and VNR. Unrelated UKB EUR samples were included for this analysis (N = 318,844 for EDU, 319,536 for RT, and 128,812 for VNR). DDG2P database (https://www.deciphergenomics.org/ddd/ddgenes) was accessed on December 23, 2020. Missense variants were classified by deleteriousness (MPC) into 3 tiers: tier 1 with MPC > 3; tier 2 with 3 ≥ MPC > 2; tier 3 includes all missense variants not in tier 1 or 2. We note that the effect of damaging missense variants out scaled that of PTV burden for DDG2P genes. This is most likely explained by UKB participants being depleted for highly penetrant PTVs in this gene set that cause disease onset in childhood^13^. Data are presented in effect size estimates (β) with 95% confidence intervals. **b**. Comparison between gene-based associations for genes from DDG2P database, EDU and VNR (PTV DNM enrichment [simulation-based test] and DeNovoWEST [simulation-based test] for DD; rare PTV burden associations [two-sided *t*-test] for EDU and VNR). Each dot represents a gene that is identified for DD in Kaplanis et al. 2020 and for EDU or VNR in the current exome analysis. The dots are color-coded according to the phenotypes (DD, ASD, or EDU) that the gene is exome-wide significantly associated with. The size and shade of the dots are representing the pLI for the gene. EDU and VNR genes are labeled with gene names.

To identify individual genes linking DD, ASD and adult cognitive function, we next extracted PTV de novo mutation enrichment and de novo weighted enrichment simulation test (DeNovoWEST) *P* values^12,13^ and compared the relative impact of rare coding variants in a combined DD, ASD, EDU and VNR gene set (Fig. 2b and Supplementary Table 11). *KDM5B* and *GIGYF1* stood out from these analyses because, interestingly, both genes are LoF-tolerant despite being a cause of DD. *CACNA1A* was also notable because its association with DD was primarily driven by missense variants, whereas PTV burden was primarily associated with VNR. This is consistent with earlier findings for *CACNA1A*, in which both LoF and gain of function mutations may cause neurological diseases with a spectrum of partially overlapping clinical phenotypes^38–43^. We repeated these analyses with 2,020 confirmed or probable rare disease genes from the Developmental Disorder Genotype-Phenotype Database (DDG2P) and observed similar results (Extended Data Fig. 2 and Supplementary Tables 10 and 11). Our analyses support that PTVs and missense variants in *KDM5B*, *GIGYF1* and *CACNA1A* underlie a continuum of conditions with various degrees of cognitive impairment.

### *KDM5B* gene dosage determines clinical phenotype

**Fig. 3:**
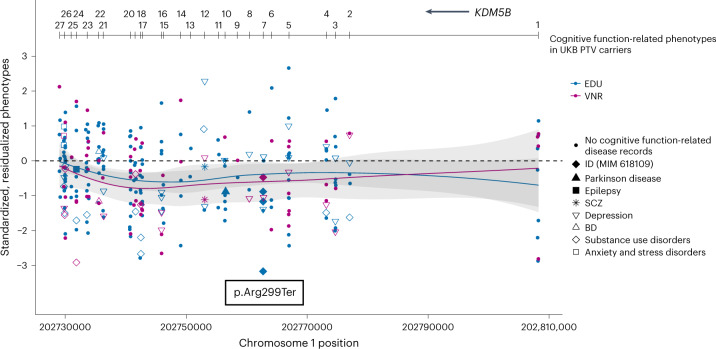
Phenotypic characterization of *KDM5B* in humans. Distribution of EDU and VNR scores for *KDM5B* PTV carriers in the UKB. ClinVar probably pathogenic variants for IDD (MIM 618109) were annotated. Samples with inpatient ICD-10 records of psychiatric (SCZ, BD, depression, substance use disorder or anxiety and stress disorders), neurodegenerative and neurodevelopmental disorders were annotated. Phenotypes were residualized by sex, age, age^2^, sex by age, sex by age^2^, top 20 principal components and recruitment centers and were rank-based inverse-normal transformed. The blue (for EDU) and red (for VNR) lines represent fitted locally estimated scatterplot smoothing (LOESS) regression on standardized, residualized phenotypes. The gray bands represent 95% CIs for the fitted LOESS regression.

**Extended Data Fig. 3:**
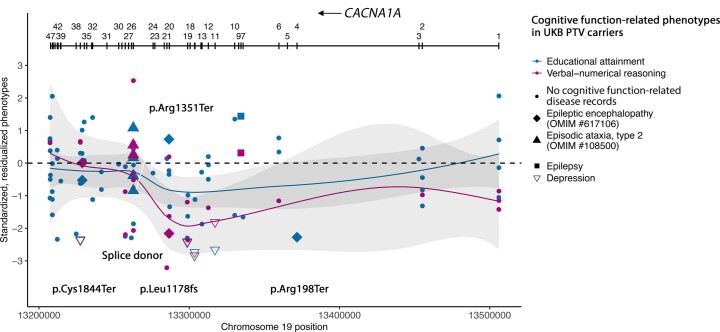
Distribution of cognitive phenotypes (educational attainment and verbal-numerical reasoning) for *CACNA1A* PTV carriers. ClinVar pathogenic/likely pathogenic variants for epileptic encephalopathy (MIM 617106) and/or type 2 episodic ataxia (MIM 108500) was annotated. Samples with inpatient ICD-10 (International Classification of Diseases version-10) records of psychiatric (schizophrenia, bipolar disorder, depression, substance use disorder and/or anxiety and stress disorders), neurodegenerative and neurodevelopmental disorders were annotated. Phenotypes were residualized by sex, age, age^2^, sex by age interaction, sex by age^2^ interaction, top 20 PCs, and recruitment center and inverse rank-based normal transformed. The blue line (for EDU) and red line (for VNR) represent fitted loess regression on standardized, residualized phenotypes. The gray bands represent 95% confidence intervals for the fitted loess regression.

Homozygous (HOM) and compound heterozygous (HET) mutations in the histone lysine demethylase encoded by *KDM5B* cause an autosomal recessive intellectual developmental disorder (IDD) with dysmorphic features (MIM 618109) (refs. ^44,45^). In a HET state, *KDM5B* PTVs were overrepresented in the cases of the Deciphering Developmental Disorders study^7^. To better understand the phenotypic spectrum of *KDM5B* LoF, we examined the phenotypes documented for *KDM5B* PTV carriers in UKB EUR samples (Fig. 3). As expected, EDU and VNR were on average lower in *KDM5B* PTV carriers (*n* = 204 for EDU and *n* = 79 for VNR) than in noncarriers (standardized, residualized phenotype mean = −0.3669 for EDU and −0.5387 for VNR). We identified 35 *KDM5B* PTV carriers who had been diagnosed with psychiatric disorders, epilepsy or Parkinson disease based on hospital diagnostic codes (enriched for disease cases compared with EUR non-*KDM5B* PTV carriers; *P* = 0.0005). EDU was impaired to a similar extent in *KDM5B* PTV carriers with and without such comorbidities (Supplementary Table 12). Notably, all individuals carrying HET PTVs annotated as pathogenic or likely pathogenic in ClinVar showed reduced EDU and VNR to a similar degree as carriers of novel *KDM5B* PTVs, and none of the three UKB participants HET for the pathogenic p.Arg299Ter variant (rs1558498928) had diagnostic records of IDD. We also found similar evidence for low cognitive function irrespective of comorbidities among *CACNA1A* PTV carriers (Extended Data Fig. 3 and Supplementary Table 13).

**Fig. 4:**
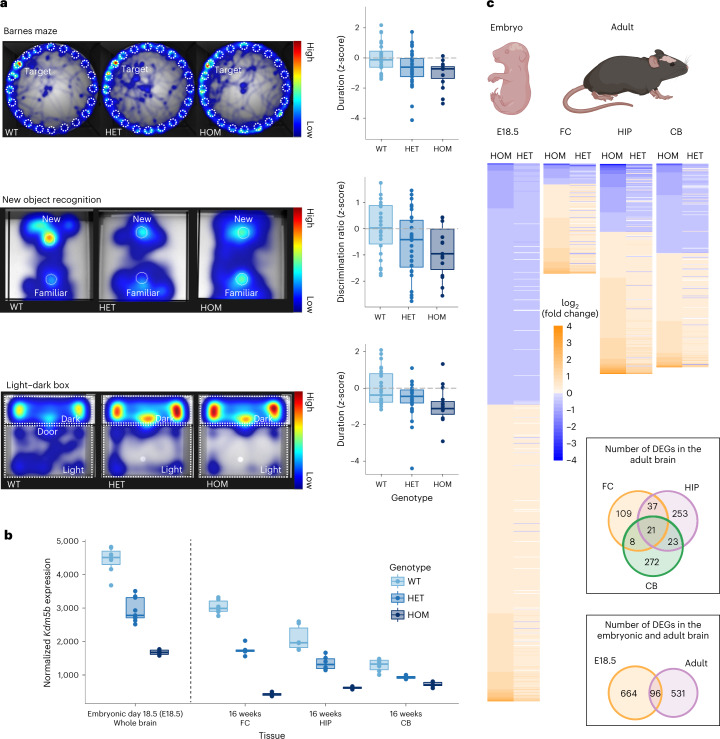
*Kdm5b* LoF alleles display a gene dosage effect on behavioral, cognitive and molecular phenotypes in mice. **a**, Mice carrying *Kdm5b* LoF alleles showed a dose-dependent decrease in spatial memory performance (Barnes maze; two-sided Wald test based on an additive genetic effect with *P* = 0.012; *Kdm5b*^+/−^ *n* = 34 (*P* = 0.031) and *Kdm5b*^−/−^ *n* = 15 (*P* = 0.005) mice spent less time around the goalbox than WT controls, *n* = 24); showed a dose-dependent decrease in object recognition memory performance (new object recognition; two-sided Wald test based on an additive genetic effect with *P* = 0.042; *Kdm5b*^+/−^ *n* = 32 (*P* = 0.038) and *Kdm5b*^−/−^ *n* = 15 (*P* = 0.011) mice had reduced discrimination compared to WT controls, *n* = 26); and showed a dose-dependent increase in anxiety-related behavior (light–dark box; two-sided Wald test based on an additive genetic effect with *P* = 0.008; *Kdm5b*^+/−^ *n* = 15 (*P* = 0.025) and *Kdm5b*^−/−^ *n* = 34 (*P* = 0.004) mice spent less time in the light compared to WT controls). *P* values are based on two-sided Wald tests from a double generalized linear model (dglm v.1.8.5). For the box plot, the center line represents the median, the box limits represent the interquartile range (IQR) and the whiskers indicate the minimum and maximum values. The heatmaps show the relative time spent around various arenas during the trial period of each assay, as a composite of all mice of the same genotype (Barnes maze and light–dark box) or the trace for a single representative animal (new object recognition). *Kdm5b*^+/−^ (HET) and *Kdm5b*^−/−^ (HOM) mice spent less time around the goalbox (Barnes maze), showed reduced discrimination of the new object (new object recognition) and spent more time in the dark zone (light–dark box) compared with WT controls. **b**, Normalized RNA-seq read counts of *Kdm5b* gene expression in WT, *Kdm5b*^+/−^ and *Kdm5b*^−/−^ mice across embryonic and adult tissues as indicated (*n* = 7 for WT, *n* = 7 for *Kdm5b*^+/−^ HET and *n* = 8 for *Kdm5b*^−/−^ HOM embryonic mice whole brain; *n* = 6, 5 and 6 for the FC of WT, HET and HOM adult mice; *n* = 6, 6 and 6 for the HIP of WT, HET and HOM adult mice; *n* = 6, 6 and 6 for the CB of WT, HET and HOM adult mice). For the box plot, the center line represents the median, the box limits represent the IQR and the whiskers indicate the minimum and maximum values. **c**, Heatmap of expression changes (log_2_ fold change) in DEGs in *Kdm5b*^+/−^ (HET) and *Kdm5b*^−/−^ (HOM) mice across embryonic and adult tissues as indicated. There is a strong correlation between direction of change in expression in both mutant genotypes. The Venn diagrams show the overlap of DEGs in both *Kdm5b*^+/−^ and *Kdm5b*^−/−^ mice across tissues and stages. Created with BioRender.com. Source data

**Extended Data Fig. 4:**
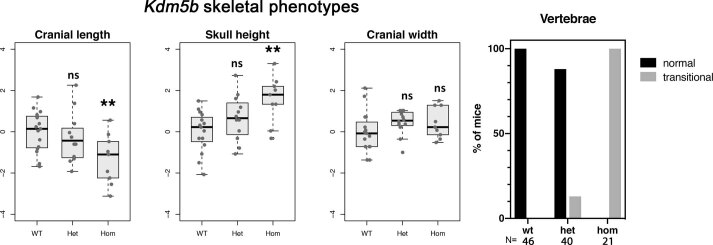
*Kdm5b* loss-of-function impacts craniofacial and skeletal features in mice in a dose-dependent manner. An intermediate effect on cranial length (additive genotype effect two-sided *P* = 0.0268) and height (additive genotype effect two-sided *P* = 0.0056) is detected in *Kdm5b*^*+/−*^ mice, but not in cranial width (additive genotype effect two-sided *P* = 0.3090). For the boxplot, the center line represents the median, the box limits represent the IQR, and the whiskers indicate the minimum and maximum values. A fully penetrant transitional vertebrae phenotype seen in *Kdm5b*^*−/−*^ mice (N = 21) is observed at a lower frequency in *Kdm5b*^*+/−*^ mice (N = 40, Fisher’s exact test vs *Kdm5b*^*+/+*^ two-sided *P* = 0.0189). Source data

**Extended Data Fig. 5:**
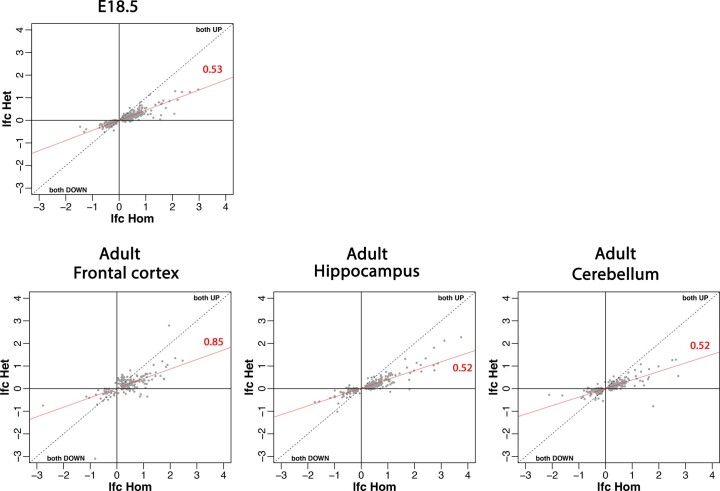
Correlation in differential gene expression between heterozygous and homozygous *Kdm5b* mutant mice. Log2-fold change of differentially expressed genes plotted for *Kdm5b*^+/*−*^ (y-axis) and *Kdm5b*^*−/−*^ (x-axis) mice across embryonic and adult brain tissues as indicated. There is a strong correlation between direction of change in expression in both mutant genotypes (robust linear regression line and slope shown in red).

Based on our findings in the UKB and *KDM5B*’s role in disease, we hypothesized that lower EDU and VNR in HET *KDM5B* PTV carriers may be explained by a gene dosage effect where HET PTV carriers show an attenuated phenotype relative to individuals with HOM *KDM5B* LoF mutations. To test this hypothesis, we conducted a series of cognitive and behavioral tests in a previously reported *Kdm5b* mouse model^45^. Relative to wild-type (WT) siblings, both HET and HOM *Kdm5b* mutant mice showed cognitive, behavioral and skeletal phenotypes consistent with an additive effect of *Kdm5b* LoF (Fig. 4a). Specifically, mutant mice showed deficits in spatial memory (Barnes maze), reduced new object recognition and behavioral abnormalities, such as increased anxiety (light–dark box). Furthermore, skeletal abnormalities observed in HOM knockout mice, such as changes in craniofacial dimensions or transitional vertebrae, were also present in HET *Kdm5b* mice with an intermediate severity or frequency (Extended Data Fig. 4). An additive effect of *Kdm5b* LoF was further supported by *Kdm5b* mRNA levels in the whole-brain tissue of embryonic HET mice and the frontal cortex (FC), hippocampus (HIP) and cerebellum (CB) tissues of adult HET mice being at an intermediate level between WT and HOM mutant mice (Fig. 4b). Consistently, 92% of the 723 differentially expressed genes (DEGs) identified by RNA sequencing (RNA-seq) (FDR *Q* < 0.1) in the *Kdm5b* mutant mouse brain showed the same directionality of change in both HOM and HET mice, with a globally smaller effect size in HET mice than HOM mice (Fig. 4c and Extended Data Fig. 5). We also found that *Kdm5b* brain expression is higher during the embryonic stages than in adult murine tissues (Fig. 4b) and followed a pattern very similar to *KDM5B* brain mRNA levels across the human life span^46^ (Supplementary Fig. 4 and Supplementary Tables 14 and 15). Consistent with the biological function of *Kdm5b*, more genes were differentially expressed in embryonic *Kdm5b* mutant mice than in adults, with a strong enrichment of genes with roles in brain development, synapse function and brain structure (Fig. 4c, Supplementary Fig. 5 and Supplementary Tables 16 and 17). In summary, our data from both mice and humans provide strong evidence that *KDM5B* LoF modulates cognition, behavior, skeletal phenotypes and brain mRNA expression in a dose-dependent manner.

### Rare and common variant signals intersect

**Extended Data Fig. 6:**
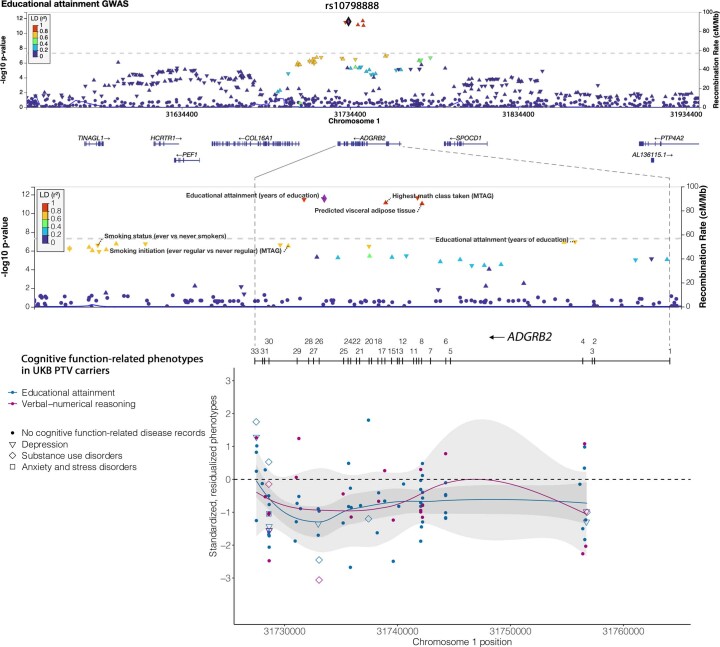
Overlap between educational attainment GWAS (Lee et al.^5^) locus on chromosome 1 and *ADGBR2* identified in PTV burden analysis in UKB. Regional plot of educational attainment GWAS association test results were generated around top independent SNP rs10798888. Additional associations from GWAS catalog were annotated with the associated phenotypes in the regional plot. EDU and VNR score for *ADGRB2* PTV carriers in UKB were plotted (both phenotypes were residualized by sex, age, age^2^, sex by age, sex by age^2^, top 20 PCs and recruitment centers and were inverse rank-based normal transformed). Samples with inpatient ICD-10 (International Classification of Diseases version-10) records of psychiatric, neurodegenerative, and neurodevelopmental disorders were annotated. The blue line (for EDU) and red line (for VNR) represent fitted loess regression on standardized, residualized phenotypes. The gray bands represent 95% confidence intervals for the fitted loess regression.

**Extended Data Fig. 7:**
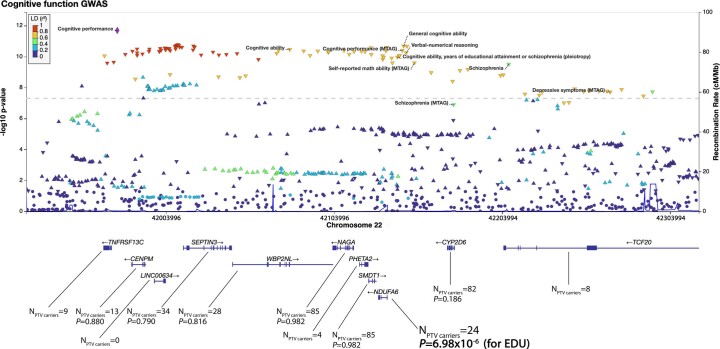
Overlap between cognitive function GWAS (Lam et al.^48^) locus on chromosome 22 and *NDUFA6* identified in PTV burden analysis in UKB (FDR significant for EDU). Regional plot of cognitive function GWAS association test results were generated for top independent SNP rs5751191 and the extended LD region. Additional associations from GWAS catalog were annotated with the associated phenotypes in the regional plot. Number of PTV carriers and gene-based PTV burden association p-value were extracted for genes in the region.

We further tested whether the genes identified through our PTV burden analysis in the UKB overlap with the genetic loci identified in previous common variant-based EDU^5^ and cognitive function GWAS^4^. Indeed, we identified overlapping signals between an EDU GWAS locus on chromosome 1 and *ADGRB2*, which showed PTV burden association with EDU. Notably, the PTVs for which carriers showed lower than average EDU and VNR and the GWAS top associated SNPs were in close genomic proximity, prioritizing *ADGRB2* as the most likely causal gene of the GWAS association signal (Extended Data Fig. 6). We also identified overlapping signals with the FDR-significant EDU gene *NDUFA6*, thus prioritizing *NDUFA6* over other genes at this GWAS locus (Extended Data Fig. 7). To further characterize the overlap between rare coding and common variant associations with cognitive function, we used UKB exomes to calculate rare coding variant burdens for genes identified in cognitive function-related GWAS. PTV burden in EDU GWAS genes showed significant effects on all three cognitive phenotypes (*β* = −0.023 and *P* = 3.69 × 10^−7^ for EDU; *β* = 0.017 and *P* = 6.38 × 10^−5^ for RT; *β* = −0.033 and *P* = 4.05 × 10^−6^ for VNR), while PTV burden in cognitive function and schizophrenia (SCZ) GWAS genes showed significant effects on EDU. Significant effects were also observed for missense variants (Supplementary Figs. 6–8 and Supplementary Table 18).

**Extended Data Fig. 8:**
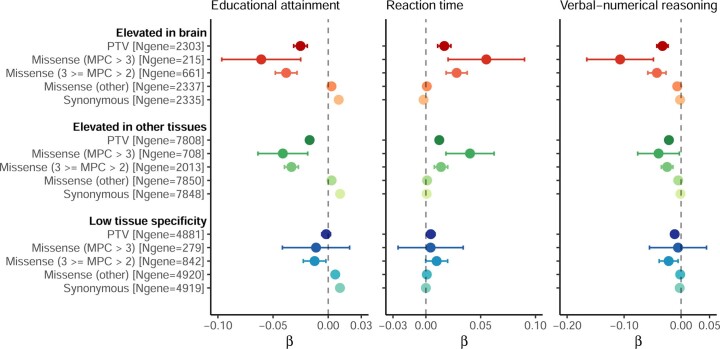
The effects of protein-truncating, missense (stratified by MPC) and synonymous variant burden in genes stratified by brain-specific expression. Unrelated UKB EUR samples were included for this analysis (N = 318,844 for EDU, 319,536 for RT, and 128,812 for VNR). Genes were stratified by elevated expression in brain tissue (2,587 genes), elevated expression in other tissues but also expressed in brain (5,298 genes) and no tissue specific expression (8,342 genes). Number of genes included in the burden is annotated for each set. Data are presented in effect size estimates (β) with 95% confidence intervals.

GWAS have identified biological pathways of potential relevance to cognitive function^2,3^. To further explore the biological mechanisms through which rare variants might impact cognitive function, we performed PTV burden analysis of 13,011 gene sets from the Molecular Signatures Database in the UKB (Supplementary Fig. 9 and Supplementary Table 19). We identified 182, 66 and 56 Bonferroni-corrected significant gene sets for EDU, RT and VNR, respectively. The most significant gene sets were involved in synaptic function, neurogenesis, neuronal differentiation and neuronal development. These signatures highly overlapped with those from cognitive function GWAS^2^, suggesting that rare and common variants modulate cognitive function through similar mechanisms. Further analyses showed that the burden of PTV and damaging missense variants in genes with brain-specific expression impacted cognitive function more strongly than when genes were primarily expressed in other tissues (Extended Data Fig. 8 and Supplementary Table 20), which is also consistent with previous GWAS findings^2,3,5^.

**Fig. 5:**
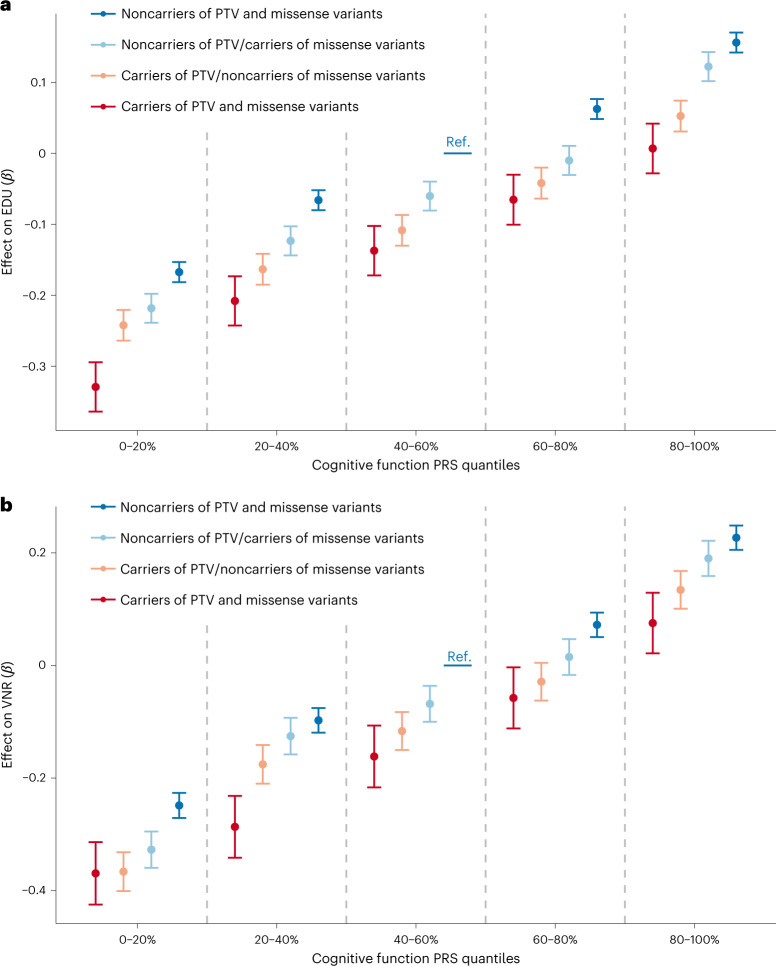
Contribution of common and rare coding variants to EDU and VNR. **a**,**b**, The impact of cognitive function PRS and carrier status of PTV or damaging missense variants (MPC > 2) in LOF-intolerant genes (pLI > 0.9) on EDU (**a**) and VNR (**b**). Unrelated UKB EUR samples were included in this analysis with *n* = 318,844 for EDU and *n* = 128,812 for VNR. EDU and VNR were residualized by sex, age, age^2^, sex by age, sex by age^2^, top 20 principal components and recruitment centers and rank-based inverse-normal transformed. The effect (and 95% CI) of PRS and rare coding variant carrier status on residualized, transformed EDU/VNR was estimated using linear regression, with noncarriers of PTV and damaging missense variants with PRS in the middle quantile as the reference (Ref.) group. Data are presented as effect size estimates (*β*) with 95% CIs.

**Extended Data Fig. 9:**
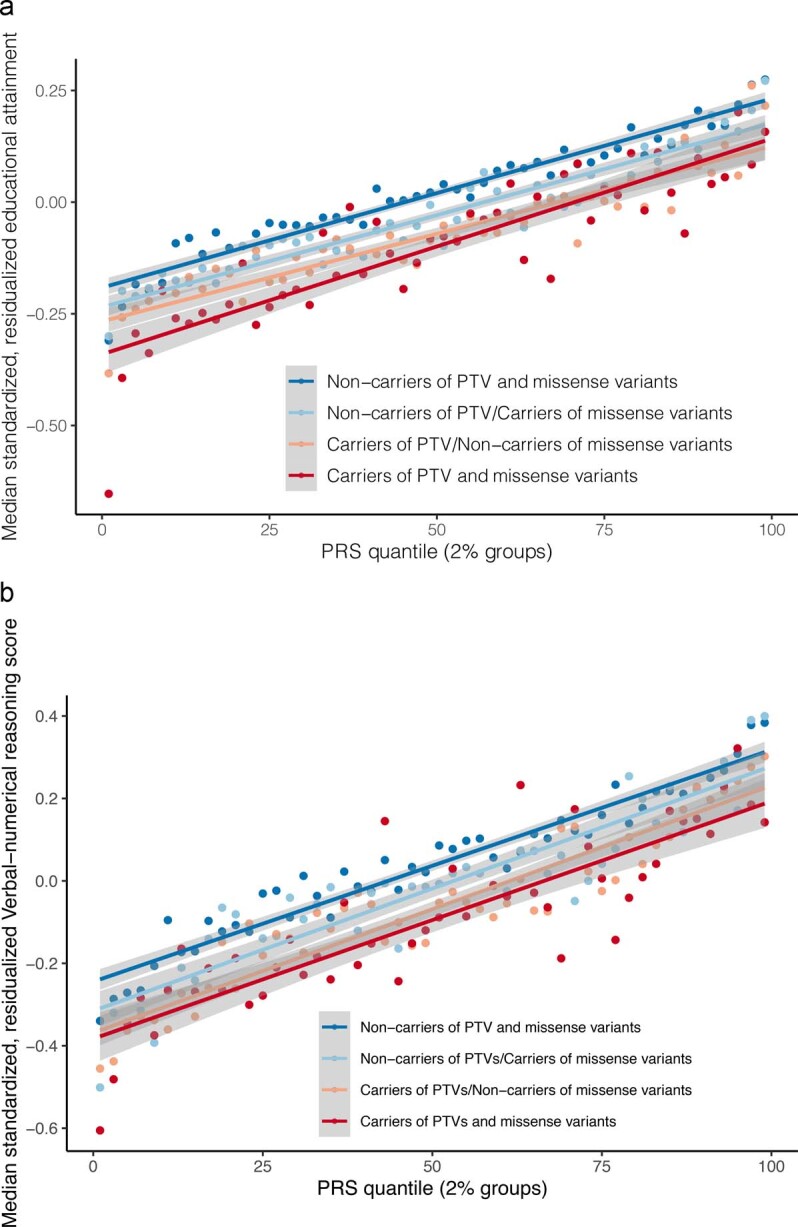
The impact of cognitive function polygenic score and carrier status of PTV and/or damaging missense variants (MPC > 2) in LoF intolerant genes (pLI > 0.9) on a) educational attainment and b) verbal-numerical reasoning. The impact of cognitive function polygenic score (PRS) and carrier status of PTV and/or damaging missense variants (MPC > 2) in LoF intolerant genes (pLI > 0.9) on a) EDU and b) VNR. EDU and VNR were residualized by sex, age, age^2^, sex by age, sex by age^2^, top 20 PCs and recruitment centers and inverse rank-based normal transformed. Median of educational attainment was calculated for individuals stratified by PRS quantiles (in 2% groups) and PTV and/or damaging missense variant carrier status. The blue and red lines represent fitted linear regressions for standardized, residualized phenotypes by PRS percentile groups, stratified by rare coding variant carrier status. The gray bands represent 95% confidence intervals for each of the fitted linear regression.

Finally, we explored the relationship between rare coding variants and common variant-based polygenic risk on cognitive function. We calculated polygenic risk scores (PRS) in unrelated EUR samples in the UKB using imputed genome-wide genotype data and SNP weights based on cognitive function GWAS (excluding the UKB samples)^4^ using PRS-CS^47^, where a higher PRS reflects the genetic liability of increased cognitive function. We tested the joint effects of PRS and carrier status for PTVs or MPC > 2 damaging missense variants in LOF-intolerant genes (pLI ≥ 0.9) on EDU and VNR. These analyses showed that the effects of PRS and rare coding variants are additive (PRS interaction test *P* = 0.27 for PTV and *P* = 0.21 for damaging missense for EDU, *P* = 0.72 for PTV and *P* = 0.59 for damaging missense for VNR; Fig. 5, Extended Data Fig. 9 and Supplementary Tables 21 and 22). For EDU, the conditional effects of PRS, PTV carrier status and damaging missense carrier status were 0.116, −0.095 and −0.053, respectively while the adjusted partial *R*^2^ values were 0.013, 0.0015 and 0.0005, respectively (*P* = 8.96 × 10^−949^ for PRS, 7.33 × 10^−109^ for PTV and 6.76 × 10^−38^ for missense variants). Similar results were observed for VNR. Our results suggest that the genetic prediction of cognitive function through PRS can be further refined by integrating rare coding alleles.

## Discussion

In this study, we present a large-scale exome sequencing study on cognitive function phenotypes in the adult general population. Our findings support previous evidence that an increased exome-wide burden of rare PTVs is associated with lower cognitive function^14,15^ and extend this observation to deleterious missense variants. The large number of exome-sequenced participants in the UKB allowed us to identify eight distinct cognitive function genes, with additional evidence from three independent EUR cohorts. Notably, several of these cognitive function genes have established roles in neurodevelopmental disorders. Our results suggest that a fraction of adults in the normal general population have lower cognitive abilities as a consequence of defects in single disease genes.

Our study is a natural extension of previous GWAS on cognitive function and EDU^2–6^. While highly successful in identifying associated loci through common variants, applying the GWAS approach to cognitive function has received substantial criticism, especially on potential biases due to ancestry, geography and environmental or cultural differences between subpopulations^25,48^. Cognitive function is difficult to assess in isolation for its substantial genetic and nongenetic overlap with other traits^25^. For instance, EDU is not only reflective of childhood and adult IQ, but also strongly correlates with other traits including income, parental age at birth, alcohol dependence or neuroticism^25^. Furthermore, as suggested by recent studies, EDU is a combination of multiple factors at both phenotypic and genetic levels^24,49,50^. Using GWAS of EDU and general cognitive function, common genetic associations of EDU have been shown to contain components of both general cognitive ability and noncognitive skills and overlap with psychiatric disorders^24,49,50^. Nevertheless, we are confident that the results of our exome study are less susceptible to such biases than GWAS. First, we analyzed three distinct phenotypes (EDU, RT and VNR) that each capture different aspects of general cognitive function^49^. The consistency of exome-wide, gene set-level and gene-level associations across EDU, RT and VNR, which also translate to independent exome-sequenced cohorts, increases the confidence that our gene findings are indeed biologically relevant. Second, five of the eight cognitive function genes (*KDM5B* (ref. ^12,13^) (MIM 618109), *ANKRD12* (ref. ^30^), *CACNA1A* (MIM 183086, MIM 108500 and MIM 617106)*, GIGYF1* (refs. ^12,13^) and *BCAS3* (MIM 619641)) are either established Mendelian DD genes or have also been identified in previous exome studies on SCZ^30,51^, DD^13^ or ASD^12^. The biological relevance of the genes discovered in this study is consistent with the well-established tight genetic relationships between cognitive traits and diseases. Third, multiple lines of evidence from our analyses yielded clues to a gene’s biological mechanisms and relevance to cognitive function. For instance, *ANKRD12* was associated with both EDU and VNR in our exome-wide PTV burden association tests; it is also associated with dysarthria and anarthria, myasthenia gravis and disorders of calcium metabolism in our PTV burden PheWAS. This suggests that cognitive dysfunction in individuals with *ANKRD12* LoF is accompanied by imbalances in motor coordination or muscle function and might be part of a yet undescribed genetic syndrome. Likewise, *ADGRB2*, on top of its association with EDU exome-wide PTV burden analyses, was associated with impairment of cognitive function (ICD-10 code R41.8) in our PTV burden-based PheWAS.

A particularly intriguing example for how diseases with cognitive impairment and adult cognitive function intersect is *KDM5B*, a gene that encodes a histone lysine demethylase with roles in neuronal differentiation^45,52–54^, which we interrogated further in humans and mice. Biallelic mutations in *KDM5B* cause an autosomal recessive IDD^44,45,53^, while HET PTVs have been linked to severe DD^7,13,45^ and ASD^12^ with presumed incomplete penetrance^45,55^. However, because previous studies focused on patients, the relevance of *KDM5B* LoF in adult cognitive function in the general population has not yet been appreciated. Unlike most other DD genes^13^, *KDM5B* is LoF-tolerant, leading to a relatively high PTV carrier rate of approximately 1:1,900 participants in the UKB. As UKB participants tend to be healthier and more educated than the general UK population^56,57^, it can be expected that *KDM5B* PTV carrier rates in the general EUR population are even higher. Our results strongly suggest a gene dosage effect for *KDM5B*, where biallelic, near-complete LoF for *KDM5B* will lead to more severely impaired cognitive function as observed in patient cohorts, whereas HET *KDM5B* LoF will present with only moderate cognitive impairment that overlaps with the spectrum of cognitive function in the normal population. Notably, *KDM5B* showed pleiotropic effects on muscle strength, bone density, growth hormone levels and BD, among others, in our PheWAS. This pleiotropy partially overlaps with the dose-dependent cognitive, behavioral and skeletal symptoms in our *Kdm5b* mouse model^45^ as well as *KDM5B* patients^44^. It will be interesting to investigate the phenotypic spectrum of *KDM5B* LoF in humans more comprehensively, for instance, through PheWAS in additional biobanks or targeted follow-up of PTV carriers in recall studies. Genes with a dosage sensitivity like the one described in this study for *KDM5B* are ideal drug targets because the degree of genetic impairment may guide the development of gene-directed therapeutic interventions^58,59^. *KDM5B* is already an established drug target with molecules inhibiting its enzymatic activity in preclinical development for cancer^60^. It could be interesting to explore whether activators exist that might improve cognitive phenotypes^61,62^.

A particular strength of exome studies is that genes and variants identified through rare variant tests tend to exhibit much larger effect sizes than common variants identified in GWAS. For example, HET carriers of *KDM5B* PTVs show on average fewer than 1.51 years of schooling than noncarriers. In contrast, lead SNPs in the most recent EDU GWAS based on three million individuals only show a median 1.4 week increase in schooling per allele (with the 5th and 95th percentiles of the estimated effect being 0.9 and 3.5 weeks, respectively)^6^. This demonstrates that exome studies may uncover substantially stronger genetic effects and complement GWAS to describe the genetic architecture of cognitive function more comprehensively. This is further supported in the case of *ADGRB2* and *NDUFA6*, which our results suggest as the most probable causal genes in loci identified in EDU and cognitive function GWAS^5^.

With both exome sequencing and genome-wide genotype data in the UKB, we were able to explore the relative contribution of common variant-based polygenic risk and rare coding variant burden to cognitive function. Our results provide evidence that rare coding variants affect EDU and VNR additively to PRS and thus suggest that genetic prediction can be further improved by combining PRS and the rare coding variant burden. Similar findings were reported previously for other common complex phenotypes^51,63–65^. Although the phenotypic variance explained by rare coding variants is much smaller than that explained by PRS because of allele frequency constraints, rare coding variants provide orthogonal predictive power that is not relying on external training GWAS (like PRS) and is thus less susceptible to biases^66^.

Future studies are needed to better understand the biological basis of how the genes and variants reported in this study impact cognitive function and related diseases. Moreover, our findings do not imply direct applications in clinical practice, such as for prenatal genetic screening^67,68^, and should be interpreted with similar caution as reported in GWAS^6^. Further work is also needed to assess how well the results from our study can be extrapolated to ancestries other than EUR populations. Nevertheless, our results provide a starting point toward expanding our knowledge on how rare genetic variants impact cognitive function at the population level and support a convergence of rare and common genetic variations that jointly contribute to the spectrum of cognitive traits and diseases.

## Methods

The UKB is approved by the North West Multi-centre Research Ethics Committee (https://www.ukbiobank.ac.uk/learn-more-about-uk-biobank/about-us/ethics). The current study was conducted under UKB application no. 26041. The data in the UKB were collected after written informed consent was obtained from all participants. The Human Research Committee of the MGB approved the Biobank research protocol (no. 2009P002312) (ref. ^69^). The data in the MGBB were collected after broad-based written consent was obtained from all participants. The Coordinating Ethical Committee of the Helsinki and Uusimaa Hospital Region approved the SUPER-Finland study on 16 July 2015 (pilot) and 9 February 2016 (full study). All participants of the SUPER-Finland study signed an informed consent that permits research use of collected samples and data. The ethical committees of the Northern Ostrobothnia Hospital District and the Hospital District of Helsinki and Uusimaa approved the NFID study. All participants or their legal guardians provided written informed consent to participate in the study. The breeding and housing of mice, and all procedures for the *Kdm5b* LOF mouse experiments were assessed by the Animal Welfare and Ethical Review Body of the Wellcome Sanger Institute and conducted under a UK Home Office license (no. P6320B89B) in accordance with institutional guidelines.

### Cognitive function phenotypes in UKB

The UKB is a prospective cohort study of the UK population with over 500,000 participants^16^. Participants were aged between 40 and 69 years at recruitment in 2006–2010 and provided extensive phenotypic data^17^. We extracted three cognitive function phenotypes for our analysis: EDU; RT; and VNR^23^. EDU is based on a survey of years of schooling that reflects both cognitive function and noncognitive components^24^. We extracted the UKB data field 6138 ‘Qualifications’ collected at baseline as a measure of EDU and converted multiple-choice categories to years of schooling as outlined by Lee et al.^5^. RT is a measure of processing speed, which is a component of general cognitive function^2,4,26,27^, implemented as a digital assessment at baseline (UKB data field no. 20023) (ref. ^2^). VNR is a score measured using a structured questionnaire, which contains 13 questions that focus on assessing crystalized and fluid intelligence in both verbal and numerical aspects (UKB data field no. 20016). Note that only a subset of 165,453 UKB participants completed the VNR assessment at baseline, while EDU (*n* = 497,844 at baseline collection) and RT (*n* = 496,660 at baseline collection) data were collected for almost the entire UKB. For the association analyses in the UKB samples, we rank-based inverse-normal transformed the phenotypes. While higher EDU and VNR scores indicate better cognitive function, longer RT represents worse cognitive function.

### The UKB whole-exome sequencing data

Whole-exome sequencing (WES) data from UKB participants was generated by the Regeneron Genetics Center on behalf of the UKB Exome Sequencing Consortium, which is a collaboration between AbbVie, Alnylam Pharmaceuticals, AstraZeneca, Biogen, Bristol Myers Squibb, Pfizer, Regeneron and Takeda^18^. Briefly, WES was done on an Illumina NovaSeq 6000 platform using xGen Exome capture kits. Sequencing reads were aligned to the GRCh38 reference genome using the Burrows–Wheeler Aligner-MEM (v.0.7.17) (refs. ^18,70^). Single-nucleotide variants and indels were called by first generating gVCF files using the WeCall variant caller v.1.1.2 (Genomics PLC) and then joint-called using the GLnexus joint genotyping tool (v.0.4.0) (refs. ^18,21,71^). The joint-called, project-level VCF was then filtered by the Regeneron Genetics Center quality control (QC) pipeline (the ‘Goldilocks’ set). As of November 2020, we obtained QC-passed WES data from 454,787 UKB participants. The UKB can release these data publicly to approved researchers via their Research Analysis Platform.

We annotated variants using Variant Effect Predictor (VEP) v.96 (ref. ^28^) with genome build GRCh38. Stop-gain, splice site-disruptive and frameshift variants were further assessed by Loss-Of-Function Transcript Effect Estimator (LOFTEE) (a VEP plugin)^29^ and high-confidence predicted LOF variants were retained for analysis. Missense variants were further annotated for deleteriousness using the MPC score^31^. We also annotated variants based on gene intolerance to LOF using pLI (probability of being LOF-intolerant) v.2.1.1 (refs. ^29,32^). All predicted variants were mapped to GENCODE^72^ (release 30) canonical transcripts.

For the association analysis, we filtered variants to include only those with a MAF < 1.0 × 10^−5^ in the UKB (649,321 PTVs, 5,431,793 missense variants and 3,060,387 synonymous variants) to enrich for pathogenic variants. In previous exome studies, the impact of an exome-wide ultrarare variant burden was associated with EDU, ID and psychiatric disorders. In these studies, ultrarare variants were defined as variants observed in fewer than 1 in 74,839 individuals (allele frequency < 1.34 × 10^−5^) or 1 in 201,176 individuals (allele frequency < 2.49 × 10^−6^) (refs. ^14,15^) in external reference samples. A recent large-scale exome study for SCZ also adopted a minor allele count cutoff of fewer than 5 alleles in 24,248 cases and 97,322 population controls, which corresponds to a MAF cutoff of 2.06 × 10^−5^ (ref. ^30^). Our choice of variant filtering for MAF < 1.0 × 10^−5^ is in line with these previous studies.

### The UKB genome-wide genotype data

We used imputed genotype data provided by the UKB with additional QC filtering. Genome-wide genotyping was performed for all UKB participants and imputed with the Haplotype Reference Consortium^73^ and UK10K^74^ plus 1000 Genomes Project reference panels^75^, resulting in a total of more than 90 million variants. We performed QC on the genotyping data by filtering out variants with an imputation quality INFO score < 0.8 and variants with a MAF < 0.01 using PLINK v.2.00 (ref. ^76^). We filtered out 1,804 individuals whose reported gender differed from their genetic gender, individuals showing sex chromosome aneuploidies, as well as 133 individuals who had withdrawn from the UKB (as of 24 August 2020).

To identify UKB samples from different genetic ancestries, we performed population assignment based on population structure using principal component analysis (PCA) with the 1000 Genomes Project reference samples (*n* sample = 2,504) from five major population groups: AFR; American (AMR); East Asian (EAS); EUR; and SAS. Details of the genetic PCA-based population assignment can be found in the Supplementary Information. We identified 8,406 AFR samples, 1,085 AMR samples, 1,609 EAS samples, 458,197 EUR samples, 9,224 SAS samples and 8,874 samples without explicit population assignment. Due to the small sample sizes, we did not analyze further the samples in the EAS and AMR groups. We also did not analyze further samples without an explicit population assignment. Within-population PCA was performed for the AFR, EUR and SAS samples for subsequent association analyses.

### Gene set-based rare coding variant burden test

#### Analysis overview

To estimate the association between cognitive function phenotypes (EDU, RT and VNR) and gene set-based rare coding variant burdens, we rank-based inverse-normal transformed the phenotypes and fitted a linear regression in unrelated UKB samples in samples from the same population group (as described in the section on population assignment). To minimize potential population stratification and confounding, we adjusted for sex, age, age^2^, sex by age interaction, sex by age^2^ interaction, top 20 principal components (PCs) and recruitment centers (as categorical variables) in all association analyses. We ran additional sensitivity analyses accounting for 40 PCs to assess the potential residual population stratification and found that the exome-wide burden results were consistent (Supplementary Table 2). The effect size (*β*), 95% CIs and *P* values were calculated for each burden association. The significance level of the burden association was determined using Bonferroni correction for the number of association tests in the defined set of analysis and is provided in Supplementary Tables 1, 2, 6, 7, 10 and 18–20.

#### Exome-wide burden

To characterize the effects of exome-wide rare coding variant burden on cognitive function, we calculated the cumulative minor allele counts of rare coding variants (MAF < 1.0 × 10^−5^) for each variant functional class as defined by the VEP^28^, LOFTEE^32^, MPC^31^ and pLI scores^32^. We defined the following variant classes: PTVs; high-confidence LOF variants; missense variants classified according to deleteriousness (MPC) into tier 1 for MPC > 3, tier 2 for 3 ≥ MPC > 2 and tier 3 for other missense variants not in the previous two tiers; and synonymous variants (identified by VEP). We further classified variants according to the LOF intolerance of the gene (pLI ≥ 0.9 or pLI < 0.9) in which the variant resides. The exome-wide rare coding variant burdens for each variant class were calculated and burden association tests were performed in the EUR, SAS and AFR samples in the UKB.

#### Gene set burden

We also calculated the rare coding variant burdens for several gene sets, including genes identified in: (1) exome studies for ASD (*n* gene = 102)^11^, DD (*n* gene = 285)^13^ and the DDG2P (https://www.deciphergenomics.org/ddd/ddgenes; *n* gene = 2,020); (2) GWAS for EDU (*n* gene = 1,140) (ref. ^5^), cognitive function (*n* gene = 807) (ref. ^4^), SCZ (*n* gene = 3,542) (ref. ^77^), BD (*n* gene = 218) (ref. ^78^) and depression (*n* gene = 269) (ref. ^79^); (3) gene sets annotated in the Molecular Signatures Database (v.7.2; *n* gene set annotated = 13,011); (4) gene sets with brain tissue expression specificity annotated in the Human Brain Atlas^80^ (*n* gene annotated = 16,270). Details on the calculation of gene set burdens and association analyses can be found in Supplementary Information.

### Exome-wide, gene-based PTV burden test

To identify genes associated with adult cognitive function, we calculated the rare PTV burden for each gene and performed burden association analyses. We used two-step whole-genome regression implemented in regenie for association testing^34^. Regenie accounts for population stratification and sample relatedness, which allowed us to leverage a larger sample size by including related samples. Regenie first fits a stacked block ridge regression to obtain leave-one-chromosome-out (LOCO) genetic prediction of the phenotype of interest; in the second step, the association test is carried out by fitting regression models conditioning on the LOCO predictions derived in the first step.

For the regenie step 1 regression, we first performed sample QC and then genotype QC by excluding variants with a genotyping call rate less than 90%, Hardy–Weinberg equilibrium test *P* < 10^−15^ and MAF < 1%. This retained 565,124 genotyped variants for the step 1 regression. We fitted a regenie first-step regression for rank-based inverse-normal transformed EDU, RT and VNR separately, adjusting for sex, age, age^2^, sex by age interaction, sex by age^2^ interaction, top 20 PCs and recruitment centers with tenfold cross-validation (regenie v.1.0.6.7) (ref. ^34^). For the regenie step 2 association test, we implemented an in-house pipeline (R v.3.6.1) for rare PTV burden association tests conditioned on the first-step LOCO prediction, following the linear regression model for association testing described in the regenie publication^34^. We treated the LOCO prediction as an offset in the linear regression model where rank-based inverse-normal transformed EDU, RT and VNR were regressed on gene-based rare PTV burden, adjusting for the same covariates used in the step 1 regression. We excluded genes with fewer than ten PTV carriers from the gene-based PTV burden analysis, which leads to a variable number of tests performed for each phenotype, especially for VNR, which has a much smaller sample size. We repeated the two-step regenie regression in the UKB EUR, SAS and AFR samples.

For the EUR samples, the sample sizes and number of genes tested for each cognitive function phenotype were as follows: *n* sample = 393,758 and test *n* = 15,782 for EDU; *n* sample = 394,600 and test *n* = 15,798 for RT; and *n* sample = 159,026 and test *n* = 11,905 for VNR. The Bonferroni correction for multiple testing was based on the actual number of tests performed per phenotype and across the three phenotypes. The significance levels for the gene-based rare PTV burden association tests were Bonferroni-corrected for the number of tests for each phenotype separately, which are 0.05/15,782 = 3.17 × 10^−6^ for EDU, 0.05/15,798 = 3.16 × 10^−6^ for RT and 0.05/11,905 = 4.20 × 10^−6^ for VNR. Note that seven of the eight cognitive function genes (that is, all except *BCAS3*) identified in our PTV burden association analysis in the UKB EUR samples were also exome-wide-significant after Bonferroni correction across all tests (*P* < 0.05/43,485 = 1.15 × 10^−6^). Additionally, we identified five genes with an FDR *Q* < 0.05 for EDU and VNR in the UKB EUR samples. For the SAS samples, the sample sizes and number of genes tested were as follows: *n* sample = 8,181 and test *n* = 1,247 for EDU; *n* sample = 8,018 and test *n* = 1,187 for RT; *n* sample = 4,430 and test *n* = 331 for VNR. For the AFR samples, the sample sizes and number of genes tested were as follows: *n* sample = 7,504 and test *n* = 887 for EDU; *n* sample = 7,331 and test *n* = 844 for RT; *n* sample = 3,890 and test *n* = 179 for VNR.

### Replication cohorts

To replicate our gene findings from the exome-wide, gene-based PTV burden tests in the UKB, we performed gene set-based and gene-based PTV burden association tests in three independent cohorts with samples of EUR ancestry: the SUPER-Finland study; the NFID study; and the MGBB. Details of phenotype, genotype and exome sequencing data processing and QC can be found in the Supplementary Information. In each replication cohort, we calculated PTV burdens for two cognitive function gene sets, including the eight genes with Bonferroni-corrected significance and the 13 genes with FDR significance identified in the UKB EUR samples. We also calculated the PTV burdens of individual cognitive genes with at least five rare PTV carriers (*ADGRB2*, *KDM5B*, *GIGYF1*, *ANKRD12* and *KIF26A*) in the NFID study. PTV burden association tests were then performed between the PTV burdens and cognitive traits in the replication cohorts. For the SUPER-Finland study, association tests were performed between PTV burdens and DD/ID, academic performance compared with schoolmates and EDU using either linear or logistic regression, adjusted for ten PCs, imputed sex, sequence assay and total number of coding variants in the genome. For the NFID study, we tested associations between PTV burdens and DD/ID using a logistic regression, adjusted for sex and the top ten PCs. In addition, we performed a meta-analysis of the DD/ID association with PTV burdens between the SUPER-Finland and NFID studies using an inverse-variance weighted random-effects meta-analysis. For the MGBB, we tested the association between PTV burdens and EDU using a linear regression, which was adjusted for sex, age, age^2^, sex by age interaction, sex by age^2^ interaction and the top 20 PCs. ORs, 95% CIs and *P* values were calculated for all association tests and meta-analyses.

### Phenome-wide association analysis

To explore the cognitive function genes identified for potential pleiotropic effects, we performed a PTV burden phenome-wide association analysis across 3,150 UKB phenotypes derived semiautomatically. Binary phenotypes included ICD-10 codes from inpatient records (congenital malformations; deformations and chromosomal abnormalities; diseases of the circulatory system; diseases of the digestive system; diseases of the eye and adnexa; diseases of the genitourinary system; diseases of the musculoskeletal system and connective tissue; diseases of the nervous system; diseases of the respiratory system; diseases of the skin and subcutaneous tissue; endocrine, nutritional and metabolic diseases; mental, behavioral and neurodevelopmental disorders; neoplasms; pregnancy; childbirth and the puerperium; symptoms, signs and abnormal clinical and laboratory findings; not elsewhere classified) and death records (ICD-10 cause of death), self-reported illness (cancer, non-cancer), self-reported medication, surgery and operation codes, and family history (father’s, mother’s and siblings’ illnesses were combined into a single phenotype for each of the 12 family history illnesses ascertained in the UKB questionnaires). Quantitative phenotypes included biomarkers such as blood cell count, blood biochemistry, infectious disease antigen assays and physical measurements. A list of all phenotypes with phenotype categories, UKB field numbers and phenotype full names can be found in Supplementary Table 9.

We restricted the phenome-wide association analysis to 321,843 unrelated UKB EUR samples and excluded binary phenotypes with fewer than 100 cases in our analysis. PTV burden testing for binary phenotypes was performed in all individuals using logistic regression, controlling for sex, age, age^2^, sex by age interaction, sex by age^2^ interaction, top 20 PCs and assessment centers. For binary phenotypes with a PTV burden association *P* < 0.01, we repeated the analysis using Firth’s logistic regression to account for situations where the logistic regression outputs may be biased due to separation^81^. For quantitative phenotypes, we excluded phenotypes with fewer than 100 observations. For each quantitative phenotype, individuals with outlier phenotype values (>5 s.d. from the mean) were excluded. The PTV burden test for quantitative traits was performed using linear regression on rank-based inverse-normal transformed phenotypes in all individuals, controlling for sex, age, age^2^, sex by age, sex by age^2^, top 20 PCs and assessment centers. We defined a Bonferroni-corrected phenome-wide significance threshold (using the number of tests per gene) of 1.59 × 10^−5^ (0.05/3,150).

### Characterization of cognitive phenotypes in *KDM5B* and *CACNA1A* PTV carriers

*KDM5B* is an established Mendelian disease gene, with HOM or compound HET mutations causing autosomal recessive ID (MIM 618109) (refs. ^44,45^). Similarly, *CACNA1A* is also an established disease gene with HET mutations causing developmental and epileptic encephalopathy (MIM 617106) (refs. ^41,42^), type 2 episodic ataxia (MIM 108500) (ref. ^40^) or spinocerebellar ataxia (MIM 183086) (refs. ^38,39^). To better understand the relationship between PTVs in *KDM5B* and *CACNA1A* and cognitive function phenotypes, we first processed EDU and VNR in the UKB EUR samples by residualizing EDU and VNR with sex, age, age^2^, sex by age interaction, sex by age^2^ interaction, top 20 PCs and recruitment centers and then standardized the residuals using rank-based inverse-normal transformation. Then, we plotted the standardized, residualized EDU and VNR for each PTV carrier against the genomic position of the PTV to characterize the phenotypic distribution of *KDM5B* and *CACNA1A* PTV carriers. We further compared the standardized, residualized EDU and VNR between three groups of PTV carriers for *KDM5B* and *CACNA1A*: (1) PTV carriers who do not have any inpatient ICD-10 diagnostic codes for neurological, psychiatric or neurodegenerative disorders or carry ClinVar pathogenic or likely pathogenic variants; (2) PTV carriers with inpatient ICD-10 diagnostic codes for neurological, psychiatric or neurodegenerative disorders; (3) PTV carriers of ClinVar pathogenic or likely pathogenic variants.

### *Kdm5b* mouse model

To experimentally investigate the potential additive dosage effect of *Kdm5b* LoF, we performed behavioral tests, morphological measurements and brain differential gene expression analysis in WT, HET and HOM *Kdm5b* LoF mice. A mouse *Kdm5b* LoF allele (Mouse Genome Informatics ID: 6153378) was generated previously^45^ using CRISPR/CAS9 mediated deletion of coding exon 7 (ENSMUSE00001331577), leading to premature translational termination due to a downstream frameshift. Breeding of testing cohorts was performed on a C57BL/6NJ background. Mice were housed in specific pathogen‐free mouse facilities with a 12-hour light–dark cycle (lights on at 7:30), an ambient room temperature of 21 °C and 55% humidity at the Research Support Facility of the Wellcome Sanger Institute. They were in mixed genotype cages (2–5 mice), and housed in individually ventilated cages (GM500, Tecniplast) containing Aspen chip bedding and environmental enrichment (Nestlets nesting material and cardboard play tunnels, Datesand). Food and water were provided ad libitum.

We applied a battery of behavioral tests commonly used to study mice for signs of perturbed neurodevelopment, including light–dark box (adapted from Gapp et al.^82^), Barnes maze probe trial and new object recognition (Supplementary Information). We assessed a cohort of 25 WT, 34 HET and 15 HOM *Kdm5b* mutant male mice at 10 weeks of age. Behavioral tests were carried out between 9:00 and 17:00, after 1 hour of habituation to the testing room. Experimenters were blind to genotype; mouse movements were recorded with an overhead infrared video camera for later tracking using automated video tracking (EthoVision XT 11.5, Noldus Information Technology). We also measured mouse cranial length and width, skull height and transitional vertebrae phenotype with X-ray whole-body radiography for 15 *Kdm5b*^+/+^, 12 *Kdm5b*^+/−^ and 9 *Kdm5b*^−/−^ mice (Supplementary Information).

All statistical analyses of mouse data were performed using R (v.4.1.3). Data were first transformed to achieve normality, using Box–Cox transformation (MASS package v.7.3–55) for behavioral data (*λ* limit = −2, 2) or quantile normalization (qnorm function) for X-ray data. Testing for genotype effect was performed using a double generalized linear model (dglm package v.1.8.5). The type of object used for new object recognition had a small (6%) and significant (*P* = 0.036) effect; therefore, it was used as a covariate for Box–Cox transformation and dglm. For visualization purposes, residual values were calculated from the linear model and *z*-scores were calculated relative to WT.

We also performed differential gene expression analysis for the *Kdm5b* mouse to assess the impact of the *Kdm5b* HET and HOM mutations on brain gene expression. RNA-seq was done for whole-brain embryonic tissue, and for the FC, HIP and CB of adult WT, HET and HOM *Kdm5b* mice. Differential gene expression and log_2_ fold changes were obtained, and *P* values for differences in gene expression were calculated. A *P* threshold of 0.10 was used to identify significant differences between WT and mutant samples. In addition, Gene Ontology (GO) enrichment analysis was performed to identify functionally enriched terms in the DEGs (with a 5% FDR threshold). In all analyses, the background consisted of only genes expressed in the tissue studied. GO terms with more than 1,000 genes were excluded from the analysis. The European Nucleotide Archive (ENA) accession numbers for the RNA-seq sequences reported are listed in Supplementary Table 17. Further details on *Kdm5b* mouse RNA extraction, sequencing, data processing and analyses can be found in the Supplementary Information.

### Overlapping rare and common variant association signals

To compare and contrast the genetic loci identified through the common variant association tests in GWAS to the genes identified in our rare PTV burden analysis, we cross-checked all independent genome-wide significant variants in the most recent, largest EDU GWAS^5^ and cognitive function GWAS^4^ with the 13 cognitive function-associated genes identified in the current study. For the EDU GWAS, we assessed the independent genome-wide significant variants listed in Lee et al.^5^ (See Supplementary Table 2 for any nearby genes with significant PTV burden association with cognitive function phenotypes.) We identified one SNP, rs10798888 (chr1:31733498 (GRCh38); MAF = 0.1725; EDU association *P* = 5.15 × 10^−14^), where *ADGRB2* (PTV burden *P* = 8.55 × 10^−12^ for EDU in the UKB EUR samples) is located in a nearby region. We then extracted the region surrounding SNP rs10798888 from the full summary statistics of the EDU GWAS (excluding the 23andMe data) obtained from the Social Science Genetic Association Consortium (SSGAC), generated regional plots (https://my.locuszoom.org/)^83^ of the GWAS results and compared these with the cognitive function phenotypes (EDU and VNR) among PTV carriers in the UKB EUR samples. Variants in the GWAS regional plots were further annotated for previous GWAS associations registered in the GWAS catalog using LocusZoom’s automated annotation feature.

For the cognitive function GWAS, we processed the GWAS summary statistics from Lam et al.^4^ with a GWAS summary statistics QC pipeline^50^ and used FUMA^84^ to identify independent genome-wide-significant loci for cognitive function from the GWAS. We identified one genome-wide-significant locus with the top independent genome-wide-significant SNP rs5751191 (chr22:41974987 (GRCh38), association *P* = 2.02 × 10^−12^) that overlapped with *NDUFA6* (PTV burden *P* = 6.98 × 10^−6^, FDR *Q* = 0.016 for EDU in the UKB EUR samples). We extracted variants in the region that covered the variants in linkage disequilibrium with the top SNP rs5751191 (*R*^2^ > 0.6) to generate a regional plot and identified genes in the region to extract the corresponding PTV burden association *P* values and the number of PTV carriers in the UKB EUR samples.

### Contributions of common variants and rare damaging coding variants to EDU and VNR

We examined the relative contribution of common variants and rare damaging coding variants to cognitive function. To do so, we first calculated the PRS to capture the impact of genome-wide common variants on cognitive function, using imputed genome-wide genotypes and variant weights derived using PRS-CS^47^ based on a cognitive function GWAS meta-analysis^4^ and a precomputed linkage disequilibrium reference panel based on the 1000 Genomes Project phase 3 EUR superpopulation samples. The cognitive function GWAS meta-analysis included only samples of EUR ancestry from the latest cognitive genomics consortium (COGENT) data freeze, excluding samples from the UKB^4^. The PRS-CS global shrinkage parameter *ϕ* was set to 0.01 because cognitive function is highly polygenic^4^. Using PRS-CS-derived variant weights and QC-imputed genotypes, we calculated PRS as a weighted sum of counted alleles across the genome using PLINK v.2.00. Then, after the exome-wide burden analysis, we identified rare damaging coding variant carriers as carriers of rare PTV or damaging missense variants with an MPC > 2 in LOF-intolerant genes (pLI > 0.9) across the exome.

To demonstrate the relative impact of PRS and rare damaging coding variant carrier status, we plotted standardized, residualized EDU and VNR against PRS, stratified according to rare damaging coding variant carrier status in unrelated UKB EUR samples. The phenotypes were residualized by sex, age, age^2^, sex by age interaction, sex by age^2^ interaction, top 20 PCs and recruitment centers and then rank-based inverse-normal transformed. The samples were grouped by PRS in 20% or 2% quantiles and are shown in Fig. 5 and Extended Data Fig. 9. The median of standardized, residualized EDU and VNR was calculated and plotted for each PRS group. We further assessed the prediction performance of cognitive function PRS and rare damaging coding variant carrier status for EDU and VNR. We fitted linear regression models by regressing rank-based inverse-normal transformed EDU and VNR on PRS and rare damaging coding variant carrier status jointly, adjusted for covariates, in unrelated UKB EUR samples. The regression coefficients, association *P* values and partial *R*^2^ were estimated^85^. We further examined the interaction between PRS and rare damaging coding variant carrier status by adding an interaction term to the linear regression model and tested for significant interaction effects. We also modeled PRS as both a continuous and a binary variable by dividing samples in the top 10% PRS group versus the remaining 90%.

### Reporting summary

Further information on research design is available in the Nature Portfolio Reporting Summary linked to this article.

## Online content

Any methods, additional references, Nature Portfolio reporting summaries, source data, extended data, supplementary information, acknowledgements, peer review information; details of author contributions and competing interests; and statements of data and code availability are available at 10.1038/s41588-023-01398-8.

## Supplementary information

Supplementary Methods, Note and Figs. 1–9.

Reporting Summary

Supplementary Tables 1–23.

## Data Availability

Full summary of the PTV burden association results derived from the UKB in this study can be found in Supplementary Table 4. For instructions on how to access the UKB exome sequencing data, see https://www.ukbiobank.ac.uk/enable-your-research/research-analysis-platform. For the SUPER-Finland study, individual-level genotype and diagnosis data are available through the THL biobank (https://thl.fi/en/web/thl-biobank/for-researchers/sample-collections/super-study). For the NFID study, due to consent and EU privacy regulations (General Data Protection Regulation), individual-level data can be used for research defined in the consent. Upon reasonable requests, aggregate-level data can be requested from the Institute of Molecular Medicine, University of Helsinki (FIMM) data access committee (fimm-dac@helsinki.fi); individual-level data can be used for collaborative research given that it is within the scope of the consent. Individual-level data are handled in a dedicated computational environment designated by FIMM. MGBB data are not publicly available due to privacy and ethical restrictions. Please contact the MGBB for further information on data access (https://www.massgeneralbrigham.org/en/research-and-innovation/participate-in-research/biobank/for-researchers). All *Kdm5b* mouse RNA-seq sequences (GRCm38) can be found at the ENA archive (https://www.ebi.ac.uk/ena/browser/home) using the accession numbers listed in Supplementary Table 17. The pLI score is available at https://storage.googleapis.com/gcp-public-data–gnomad/release/2.1.1/constraint/gnomad.v2.1.1.lof_metrics.by_gene.txt.bgz. The MPC score is available at ftp://ftp.broadinstitute.org/pub/ExAC_release/release1/regional_missense_constraint/ (open access ftp site, no registration required). The Brainspan RNA-seq data are available at https://www.brainspan.org/static/download.html. The Human Protein Atlas data are available at https://www.proteinatlas.org/humanproteome/brain/human+brain. The DDG2P gene list is available at https://www.deciphergenomics.org/ddd/ddgenes. The SSGAC EDU GWAS summary statistics are available at https://thessgac.com/ (registration required); the EDU GWAS summary statistics file used in this study can be accessed at https://thessgac.com/papers/3/12 (accessible after registration). Source data are provided with this paper.

## Source data

Source Data Fig. 4, Source Data Extended Data Fig. 4 Statistical source data.

## Code availability

The code used in this study can be found at *Zenodo* (10.5281/zenodo.7822074) (ref. ^86^). The software and software packages used include: R v.3.6.1 and v.4.1.3 (packages gprofiler2 v.0.2.1, MASS v.7.3-55, dglm v.1.8.5, data.table v.1.12.8, dplyr v.1.0.0, ggplot2 v.3.3.l, GSA v.1.03.l); PRS-CS v.1.0.0 (https://github.com/getian107/PRScs); PLINK v.2.00 (https://www.cog-genomics.org/plink/2.0); PLINK v.1.90 beta (https://www.cog-genomics.org/plink/); VEP v.96 (https://useast.ensembl.org/info/docs/tools/vep/index.html); LOFTEE (https://github.com/konradjk/loftee); regenie v.1.0.6.7 (https://rgcgithub.github.io/regenie/); Hail v.0.2 (https://github.com/hail-is/hail); LocusZoom v.0.14.0 (https://my.locuszoom.org/); FUMA v.1.3.7 (https://fuma.ctglab.nl/); FastQC v.0.11.8 (https://github.com/s-andrews/FastQC); STAR v.2.7.3a (https://code.google.com/archive/p/rna-star/); featureCounts v.2.0.0 (https://subread.sourceforge.net/); and DESeq2 v.1.34.0 (http://www.bioconductor.org/packages/release/bioc/html/DESeq2.html).
