# Supplementary material for: The impact of rare protein coding genetic variation on adult cognitive function
Source: Nat Genet. 2023 May 25;55(6):927–38. doi: 10.1038/s41588-023-01398-8 (PMC10260403; doi:10.1038/s41588-023-01398-8)
Supplement: Supplementary file 1 — Supplementary Methods, Note and Figs. 1–9. [file 41588_2023_1398_MOESM1_ESM.pdf]

nature genetics

Article https://doi.org/10.1038/s41588-023-01398-8

The impact of rare protein coding genetic variation on adult cognitive function

In the format provided by the authors and unedited

Table of Contents

Supplementary Methods 2
Details on phenotype curation in UK Biobank 2
Details on UK Biobank genetic data processing for population assignment 2
Comparison between the findings from gene-based PTV burden tests in the current study and previous exome studies in UK Biobank 3
Details on gene set burden calculations 4
The SUPER-Finland study 5
The Northern Finland Intellectual Disability (NFID) study 7
Supplementary note 13
Brief summary of cognitive function-associated genes identified 13
Estimating burden heritability for rare variants with Burden Heritability Regression 16
Biogen Biobank team 18
Supplementary Figures 19
Reference 35

Supplementary Methods

Details on phenotype curation in UK Biobank

UK Biobank (UKB) participants provided extensive phenotype data, including surveys on baseline characteristics and health outcomes, specific questionnaires and assessments, health records, physical measures and biomarkers[1]. By examining three cognitive phenotypes in the UKB, educational attainment (EDU), reaction time (RT) and verbal-numerical reasoning (VNR), we aimed at capturing different aspects of cognitive function for a more comprehensive discovery and to facilitate a dissection of the genetics of cognitive function.

Educational attainment. The educational attainment survey in UK Biobank (UKB; data field 6138) is a multiple-choice question with eight choices, including 7 categories of different qualifications and an option for "Prefer not to answer". We mapped the seven categories to years-of-schooling using the International Standard Classification of Education (ISCED) scale: none of the above (no qualifications) = 7 years of education; CSEs or equivalent = 10 years; O levels/GCSEs or equivalent = 10 years; A levels/AS levels or equivalent = 13 years; other professional qualification = 15 years; NVQ or HNC or equivalent = 19 years; college or university degree = 20 years of education[2]. Educational attainment for those who selected "Prefer not to answer" was treated as missing and excluded from the analysis.

Reaction time. Reaction time is measured by a digital test "Snap" game in UKB. In the "Snap" game, the participants were presented with pairs of matched or mismatched cards on the computer screen. If the two cards were matched, participants were to push a button box as quickly as possible. The game includes first five rounds of practice and then seven rounds of the actual tests, among which four rounds present matched pairs of cards. "Mean time to correctly identify matches" (data field 20023) is the mean time to correctly identify the four rounds of matched cards (in milliseconds).

Verbal-numerical reasoning. Verbal-numerical reasoning is measured by the "fluid intelligence" questionnaire, which contains 13 multiple-choice questions that assess verbal and numerical problem solving, where 7 questions are on numerical problem solving and 6 questions are on verbal problem solving. The participants are allowed 2 minutes to answer the questions. The questions not answered in the 2 minutes limit are scored zero. The final score is the total number of correctly answered questions in two minutes (UKB data fields 20016 "Fluid intelligence score").

Details on UK Biobank genetic data processing for population assignment

We processed and QCed both imputed genotype data in UKB and genotype data from the 1000 Genomes project[3] to perform genetic principal component analysis (PCA)-based population

assignment for UKB samples. We first performed quality control on the 1KG genotype data by retaining only the SNPs on autosomes with minor allele frequency (MAF) > 1% and removed SNPs located in known long-range LD regions (chr6:25-35Mb; chr8:7-13Mb). We also removed 1 sample from each pair of related samples (greater than second degree) in 1KG. We merged the UK biobank imputed genotype data that was filtered to imputation quality INFO > 0.8 and MAF > 1% with the 1KG genotype data and performed LD-pruning at $R^2 = 0.2$ with a 500 kb window. We then computed principal components (PCs) using the LD-pruned SNPs in 1KG sample and derived projected PCs of UK Biobank samples using the SNP-wise PC loadings from 1KG samples. Using the 5 major population labels of 1KG samples as the reference, we trained a random forest model with top 6 PCs to classify UK Biobank samples into 1KG population groups. We assigned UK Biobank samples into one of the 5 populations defined with 1KG reference based on a predicted probability for a specific population group > 0.8. We identified 1,609 EAS samples, 458,197 EUR samples, 8,406 AFR samples, 9,224 SAS samples, 1,085 AMR samples and 8,874 samples without explicit population assignment. Note that the final analytical sample sizes for each population are smaller due to the fact that exome sequencing was available for only 454,787 UKB samples, while population assignment was done for all UKB samples with genome-wide genotyping data.

After initial population assignment, we performed three rounds of within-population PCA for AFR, EUR and SAS samples to identify remaining population outliers, each time removing samples with any of the top 10 PCs that was more than 5 standard deviations (SD) away from the sample average. We used the in-sample PCs derived after outlier removal for each population in subsequent analyses.

Comparison between the findings from gene-based PTV burden tests in the current study and previous exome studies in UK Biobank

We note that some of the gene-based PTV burden findings reported in our study have also been observed in a recent cross-phenotype exome study by Backman et al.[4], where gene-based burden analyses for RT were conducted using different "gene burden masks" (annotations for grouping variants to calculate burden) and MAF cut-offs than our analysis. For verbal-numerical reasoning (originally termed "fluid intelligence" by UK Biobank), Backman et al. formatted the participants' responses to each verbal-numerical reasoning question into binary traits using one of the multiple choices against other choices and examined each question separately in their gene-based burden analyses. This is not consistent with how the UKB "fluid intelligence" data were analyzed in previous cognitive function GWAS[5–7]. The total number of correctly answered questions as we analyzed in this study is consistent with the phenotype definition used in previous cognitive function GWAS and is considered a standard measure of general cognitive function[5–7].

We also note that gene-based PTV burden and kernel-based (SKAT-O[8] and SKAT[9]) association analyses for reaction time and verbal-numerical reasoning were included in a recent study by Karcewski et al.[10], with results available through the Genebass browser (https://app.genebass.org/). In that study, gene-based PTV burden, SKAT, and SKAT-O tests were performed with a much more liberal MAF filter of 0.01 compared with the current study (which used a MAF filter of 0.00001). A direct comparison of PTV-burden, SKAT, and SKAT-O test results between both studies are presented in Supplementary Table 23. In the results from the Genebass browser, we can see that association p-values are comparable between SKAT-O and burden tests, while SKAT tests yield less significant p-values. This suggests that, since SKAT-O is an optimal unified test of a kernel-based test (i.e., SKAT) and a burden test, most of the gene discovery power in SKAT-O test is from the burden test. In this case, SKAT test would not provide much additional power in identifying novel genes. This is further supported by another publication based on an interim release of the UK Biobank exome sequencing data [20]. We also note that the effect sizes of identified cognitive function genes are at least 10-folds larger in the current study than in the Genebass browser, while the PTV-burden association p-value is sometimes more significant in the Genebass browser. This is likely due to different MAF cut-offs were used in the two studies (MAF<0.01 in Genebass and MAF<0.00001 in current study). While we are aiming at identifying genes that could be subject to strong, rare PTV effects by restricting to PTV with MAF<0.00001 in UKB, we sometimes lose power for association tests due to a smaller number of PTV carriers in the analysis.

Details on gene set burden calculations

Gene set burden - ASD, DD, DDG2P exome genes

We examined the impact of rare coding variants in genes identified in autism spectrum disorder (ASD; N gene=102)[11] and developmental disorder (DD; N gene=285)[12] exome studies, as well as in DD genes listed in the Development Disorder Genotype - Phenotype Database (DDG2P; https://www.deciphergenomics.org/ddd/ddgenes). DDG2P provides a curated list of genes reported to be associated with developmental disorders and curated by clinicians as part of the Deciphering Developmental Disorders (DDD) study to facilitate clinical reporting of likely causal variants. We included 2,020 confirmed and probable DD genes from DDG2P into our analysis. ASD, DD and DDG2P gene set burdens were calculated, and burden association analyses were conducted following the analysis procedure described above in unrelated UKB EUR samples (N sample=321,843).

Gene set burden - GWAS genes

Following the same procedure as above, we performed gene set-based burden analysis in genes identified in GWAS for educational attainment (N gene=1,140)[2], cognitive function (N gene=807)[7], schizophrenia (N gene=3,542)[13], bipolar disorder (N gene=218)[14] and depression (N gene=269)[15]. The respective GWAS gene lists were taken either directly from publications or from reprocessing the publicly available GWAS summary statistics with FUMA for positional

gene mapping[16]. We also calculated rare coding variant burden for a set of randomly selected genes not linked to cognitive function or psychiatric disease (N gene=1,082), which includes 5% of all genes excluding cognitive function genes identified in this study, ASD and DD exome studies[12,17] and cognitive function, educational attainment, schizophrenia, bipolar disorder and depression GWAS[2,7,13–15]. We followed the same procedure in exome-wide burden analysis to perform the gene set burden association analysis in unrelated UKB EUR samples.

Gene set burden - MSigDB curated gene sets and pathways
To identify pathways and gene sets associated with cognitive function, we calculated PTV burden for 13,011 gene sets identified in the Molecular Signatures Database (MSigDB v7.2; accessed on 11/26/2020; parsed with R v3.6.1 with GSA vl.03.l package) and performed self-contained pathway association analysis[18]. We included all C2 canonical pathways (BioCarta N set=292; KEGG N set=186; Pathway Interaction Database [PID] N set=196; Reactome N set=1,547; WikiPathways N set=587) and C5 gene ontology (GO) pathways (Biological Process N set=7,531; Cellular Component N set=996; Molecular Function N set=1,676). Again, we followed the same procedure as above to examine the association between pathway PTV burden and cognitive function phenotypes. The significance level was determined by Bonferroni correction for the number of pathways and gene sets tested for each phenotype ($0.05/13011 = 3.84x10^{-6}$). Note that we performed self-contained pathway analysis where the null hypothesis is that there is no association between the gene set PTV burden and the cognitive function phenotype (as opposed to competitive pathway analysis)[18].

Gene set burden - Genes with brain specific and non-specific expression
To examine a potential enrichment of rare coding variant burden association among genes with brain specific expression in cognitive function, we calculated rare coding variant burdens for three gene sets defined by gene expression specificity in the Human Brain Atlas[19]: genes with elevated expression in brain (N gene=2,587); genes with elevated expression in other tissues, but also expressed in brain (N gene=5,298); and genes with expression that has low tissue specificity (N gene=8,385). The burden association analysis was done in unrelated European UKB samples with the same procedure as above.

The SUPER-Finland study

The SUPER-Finland study is a cohort of 9,125 psychotic patients in Finland. Subjects with a diagnosis of a schizophrenia spectrum psychotic disorder (ICD-10 codes F20, F22-29), bipolar I disorder (F31) or major depressive disorder with psychotic features (F32.3 and F33.3) were recruited from in- and outpatient psychiatric, general care and housing units and by advertisements in local newspapers. DNA samples were genotyped with GWAS arrays, exome sequenced and linked to a wide range of phenotypic information ascertained through a structured interview, questionnaires, and cognitive testing. After receiving written and verbal information on the study and biobank research, all participants gave written informed consent for

participating in the study. The sample collection was conducted between 2016 and 2018 and has been funded by the Stanley Center for Psychiatric Research at the Broad Institute of MIT and Harvard, Boston, USA, and forms one arm of the Stanley Global Neuropsychiatric Genomics Initiative.

Whole-exome sequencing data generation and quality control

Blood samples were collected by venipuncture for DNA extraction (2x Vacutainer EDTA K2 5/4 ml, BD, serum (Vacutainer STII 10/8 ml gel, BD) and plasma (Vacutainer EDTA K2 10/10 ml, BD) analyses. In cases where venipuncture was not possible, a saliva sample (DNA OG-500, Oragene) was collected for DNA extraction. DNA extraction from EDTA-blood tubes was performed using PerkinElmer Janus chemagic 360i Pro Workstation with the CMG-1074 kit. Saliva samples (n= 509) were incubated in +50°C overnight before DNA extraction. Saliva samples were processed using Chemagen Chemagic MSM I robot with CMG-1035-1 kit. DNA was eluted in 400 µl 10 mM Tris-EDTA elution buffer (PerkinElmer) and DNA-concentration measured with Trinean DropSense spectrophotometer. Samples were aliquoted with Tecan Genesis/Tecan Freedom Evo and shipped to the Broad institute of MIT and Harvard, Boston, USA on dry ice for genetic analyses.

Exome sequencing was performed at the Broad Institute. The sequencing process included sample prep (Illumina Nextera, IIlumina TruSeq and Kapa Hyperprep), hybrid capture (Illumina Rapid Capture Enrichment (Nextera) - 37Mb target and Twist Custom Capture - 37Mb target) and sequencing (Illumina HiSeq4000, Illumina HiSeqX, Illumina NovaSeq6000 - 150bp paired reads). Sequencing was performed at a median depth of 85% targeted bases at > 20X. Sequencing reads were mapped by BWA-MEM to the hg38 reference using a "functional equivalence" pipeline. The mapped reads were then marked for duplicates, and base quality scores were recalibrated. They were then converted to CRAMs using Picard and GATK. The CRAMs were then further compressed using ref-blocking to generate gVCFs. These CRAMs and gVCFs were then used as inputs for joint calling. To perform joint calling, the single-sample gVCFs were hierarchically merged (separately for samples using Nextera and Twist exome capture).

Quality control (QC) analyses were conducted in Hail 0.2 and the full details are described in the SCHEMA manuscript. In brief, we annotated variants as frameshift, inframe deletion, inframe insertion, stop lost, stop gained, start lost, splice acceptor, splice donor, splice region, missense, or synonymous using the Ensembl Variant Effect Predictor tool. At the genotype level, we first split multiallelic sites and retained individual calls if they had a genotype quality (GQ) ≥ 20, allelic balance (AB) < 0.1 in homozygous calls, allelic balance (AB) ≥ 0.25 in heterozygous calls and depth (DP) ≥ 10. After applying genotype filters, we excluded variants with call rates < 0.9 or if they resided within low-complexity regions (LCR). We excluded samples that were 4 median absolute deviations from the mean in any of the following metrics: call rate (callRate),

number of heterozygous calls (nHet), number of homozygous alternate calls (nHomVar), number of non-reference calls (nNonRef), number of deletions (nDeletion), number of insertions (nInsertion), number of singleton calls (nSingleton), number of SNPs (nSNPs), heterozygous-homozygous call ratio (rHetHomVar), transition-transversion ratio (rTiTv) and insertion-deletion (rInsertionDeletion). Using the Hail PC-relate function, we pruned clusters of related individuals to ensure that no two samples were second-degree or closer in relations. Individuals with a Finnish predicted ancestry ($P > 0.7$) using a Random Forest model based on PCA using 1000 Genomes as a basis were retained.

Cognitive function phenotypes for replication analysis
The SUPER-Finland study protocol included a questionnaire, a structured interview by a research nurse, physical measurements, and blood/saliva sampling. The questionnaire and interview included questions on educational attainment (http://www.julkari.fi/handle/10024/78534), academic performance and learning difficulties at school, which were derived from the Finnish Health 2000 and 2011 general population surveys. We identified PTV carriers in the cognitive genes in the primary analysis and performed association tests between cognitive gene PTV burden and cognitive phenotypes. Association tests were done with either linear or logistic regression. We regressed the phenotypes on PTV status and corrected for 10 principal components, imputed sex, sequence assay and total number of coding variants in the genome. We focused on the following phenotypes: we define a developmental disorders/intellectual disability (DD/ID) case as someone with a diagnosis of learning difficulty and intellectual disability based on diagnostic codes in HILMO (THL). In the interview data, we asked "How did you fare in studies compared to your schoolmates?" with a response encoding of 'Below average', 'Moderately', 'Better than average'. Level of education completed was a trinarized measure based on definitions in the Health 2000 survey, and is encoded as 'low', 'middle', 'high' and correlates with only having completed primary, secondary, and tertiary education.

The Northern Finland Intellectual Disability (NFID) study

The Northern Finland Intellectual Disability (NFID) study consists of 1,097 intellectual disability cases from Northern Finland Intellectual Disability (NFID) study and 11,774 controls from the FINRISK 1992-2012 and Health 2000-2011 studies[20]. The details of the study sample recruitment and phenotyping, exome sequencing data generation and quality control and ethical permissions were described in Kurki et al. 2019[20].

The Mass General Brigham Biobank

The Mass General Brigham Biobank (MGBB) is a hospital-based biobank aiming at collecting blood samples, lifestyle and family history survey data, as well as electronic health record linkage from consented participants[21]. The release used for this study (as of November 2021)

includes 24,787 samples that were whole-exome sequenced and genome-wide genotyped in two batches. All MGBB patients gave informed consent for general biobank research.

Genotype and sample quality control

We conducted QC of genome-wide genotypes for 24,787 samples following a QC pipeline (https://github.com/Annefeng/PBK-QC-pipeline) by using PLINK v1.90, R, and python scripts. The following filters were used in sequence: variant call rate>0.95; sample call rate>0.98; second round variant filter with call rate>0.98. Variant-level missing rate was computed in each batch and variants with missing rate difference > 0.75% were filtered out. After merging two genotyping batches, we further removed duplicated variants, monomorphic variants and variants not confidently mapped to any chromosomes.

To identify MGBB samples of European ancestry, we leveraged 1000 Genomes (1KG) Project phase 3 samples as population reference. To do so, we first combined MGBB genotypes with 1KG genotypes (N sample=2,504)[3]. We only retained overlapping variants with MAF>0.05 and call rate>0.98 and filtered out multi-allelic and strand ambiguous variants. We LD-pruned variants at $R^2 = 0.1$ with window size 200 kb to obtain independent variants for principal components analysis (PCA), while excluding variants in long-range LD regions (chr6:25-35Mb and chr8:7-13Mb). With 1KG super population labels (African [AFR], American [AMR], East Asian [EAS], European [EUR], and South Asian [SAS]), we used top 6 PCs to train a random forest model and assigned MGBB samples into five populations (prediction probability>0.8). We identified 17,287 (69.7%) EUR samples for the subsequent analysis.

We further QCed MGBB EUR samples by filtering out 513 samples, including samples whose reported sex was different from genetically imputed sex (F-statistics<0.2 imputed as female; F-statistics>0.8 as male), samples with outlying heterozygosity rate (>5 standard deviation from the mean) and one of each pair of related samples (pi-hat>0.2). After removing variants showing significant batch effects ($P<1.0\times10^{-4}$), we performed PCA of QC-ed EUR samples and removed 73 outlier samples (6 standard deviations away from the mean in top 10 PCs). A total of 16,701 samples of European ancestry were retained as the final analytical sample. In-sample PCs were used to control for population stratification in the replication analysis.

Whole-exome sequencing data generation and quality control

Whole-exome sequencing was done in 26,421 MGBB samples using Illumina NovaSeq with a custom exome panel (TWIST Biosciences). The sequencing coverage was 20X for more than 85% of exonic target. Variants were joint-called by Genome Analysis ToolKit (GATK) GVCF workflow with HaplotypeCaller in gVCF mode. WES data quality control was done with Hail v0.2 (https://github.com/hail-is/hail). We first split multi-allelic variants into bi-allelic and retained high-quality variants with variant level genotype quality (GQ)>20, call rate>0.9, allele count>0, 200>mean depth (DP)>10, allele balance (AB)>0.9. Then, variants were separated into

SNPs and indels for hard filtering. For SNPs, we kept SNPs with QualByDepth (QD)≥2, FisherStrand (FS)≤60, StrandOddsRatio (SOR)≤3, RMSMappingQuality (MQ)≥40, MappingQualityRankSumTest (MQRankSum)≥-12.5 and ReadPosRankSumTest (ReadPosRankSum)≥-8. For indels, we kept variants with QD≥2, ReadPosRankSum≥-20, FS≤200 and SOR≤10. We retained 10,588,646 high-quality variants after QC. With high-quality variants, we then performed sample-level QC by keeping samples with number of singleton (n.singleton)<500, sample-level genotype quality (GQ)>40 and sample call rate>0.9.

Statistical analysis

We performed replication analysis on MGBB samples of European ancestry with genotype, whole-exome sequencing and educational attainment data using R v3.6.1 (with packages data.table vl.12.8, dplyr vl.0.0, and ggplot2 v3.3.l). The total analytic sample size is 8,389. We first extracted variants in the 8 genes identified in UKB gene-based PTV burden analysis from the MGBB exome data. We annotated the coding variants with Variant Effect Predictor (VEP) v96[22] and Loss-Of-Function Transcript Effect Estimator (LOFTEE)[23]. We identified 28 PTVs in the 8 genes and 36 PTVs in 13 genes with MAF ranging from $1.89x10^{-5}$ to $7.57x10^{-5}$. We calculated PTV burden across 8 or 13 cognitive genes in MGBB European samples and performed association tests between cognitive gene PTV burden and educational attainment. Educational attainment in MGBB was self-reported and converted from categories of educational levels to years-of-education following the sample rules used in processing UKB educational attainment data. We used linear regression for association testing, adjusted for sex, age, age^2, sex by age interaction, sex by age^2 interaction and top 20 PCs.

Details on Kdm5b mouse behavioral testing

We applied a battery of behavioral tests as they are commonly applied to study mice for signs of perturbed neurodevelopment, including light-dark box, Barnes Maze probe trial, and Novel Object Recognition. The details of these behavioral tests are as follows:

Light-dark box

This test was adapted from Gapp et al.[24]. Mice were housed in pairs or trios for at least 10 minutes before performing a grip strength test (BIO-GS3, Bioseb) and subsequently introducing them individually into the light-dark box, a plastic box (40 × 42 × 26 cm) divided in two compartments. One is smaller, closed, and dark (1/3 of the total surface area) and connected through a door (5 cm) to a larger, brightly lit (370 lux with an overhead lamp) compartment (2/3 of the box). Each mouse was placed in the dark compartment, the door was then opened, and the animal was left to explore for 10 min. The time spent in the light compartment was assessed and the difference between genotype groups was calculated as z-scores.

Barnes Maze probe trial

This assay is a test of visuo-spatial learning and memory on a circular maze (120 cm diameter table) with 20 holes around the perimeter[25]. One of the holes leads to a small dark box (Target) where the mice can escape from the brightly lit maze. Mice were trained for three days, 10 trials (4 min maximum each), to find the target location. On the probe trial, 72 hours after the last training day, the escape box was removed. Each mouse was given 4 minutes to explore the maze. The mouse's movements were tracked, and the amount of time spent around each of the holes during the first minute of the test was analyzed. Analysis results are expressed as z-scores of homozygote or heterozygote relative to wildtype mice.

Novel Object Recognition

This assay was conducted as part of an Object Displacement - Novel Object Recognition test on a square arena (37 cm side). Mice were first habituated to the arena for 20 min. The following day mice were tested during two 10min trials, with an inter-trial-interval of 1 hr. During these trials, mice were left to explore two identical objects (either small glass bijou bottles or similarly sized halogen light bulbs). 24 hr later, on day 3, mice were given the choice to explore two different objects, a familiar one (to which they had been exposed the previous day) and a novel one. The movement of each mouse was tracked and the amount of time their nose was in proximity to the objects was recorded and used as investigation time. The preference for investigating the novel object was calculated as a ratio, Preference Novel= $T_{novel}/(T_{novel} + T_{familiar})$, where T_{novel} and $T_{familiar}$ are the amount of time spent investigating the novel and familiar objects. Mice prefer to investigate novel objects over familiar ones, so deviation from this bias is interpreted as reduced recognition memory sensitivity or discriminatory ability [26].

Details on Kdm5b mouse whole body radiography

Fifteen *Kdm5b*$^{+/+}$, twelve *Kdm5b*$^{+/-}$ and nine *Kdm5b*$^{-/-}$ mice were anesthetized with ketamine/xylazine (100mg/10mg per kg of body weight) and then placed in an MX-20 X-ray machine (Faxitron X-Ray LLC). Whole body radiographs were taken in dorso-ventral and lateral orientations. Images were then analyzed, and morphological abnormalities assessed using Sante DICOM Viewer v7.2.1 (Santesoft LTD). To sufficiently power the analysis of transitional vertebrae in heterozygous animals, we analyzed a larger number of animals (*Kdm5b*$^{+/+}$*:* 46*, Kdm5b*$^{+/-}$*: 40, Kdm5b*$^{-/-}$:21).

Details on Kdm5b mouse RNA extraction, sequencing, and data processing

Mouse tissues were homogenized in buffer RLT plus (Qiagen) with β-mercaptoethanol (Sigma, M3148; 10µl/ml) using Qiagen TissueLyser LT, with sterile steel beads and operated at 50Hz for 2 minutes. Samples were passed over gDNA eliminator columns. Then total RNA was extracted on RNeasy Plus columns as per manufacturer's protocol (Qiagen), immediately snap frozen on dry ice and stored at -80C. An aliquot of each sample was quantified using 2100 Bioanalyzer (Agilent Technologies). RNA sequencing libraries were prepared using established protocols: library construction (poly(A) pulldown, fragmentation, 1st and 2nd strand synthesis, end prep and ligation) was performed using the NEB Ultra II RNA custom kit (New England Biolabs) on

an Agilent Bravo automated system. Indexed multiplexed sequencing was performed on an Illumina HiSeq 4000 system, using 75 bp paired-end sequencing reads. The sequencing data were de-multiplexed into separate CRAM files for each library in a lane. Adapters that had been hard-clipped prior to alignment were reinserted as soft-clipped post alignment, and duplicated fragments were marked in the CRAM files. The data pre-processing, including sequence QC and STAR alignments were made with a Nextflow pipeline, which is publicly available at https://github.com/wtsi-hgi/nextflow-pipelines/blob/rna_seq_mouse/pipelines/rna_seq.nf, including the specific aligner parameters. We assessed the sequencing data quality using FastQC v0.11.8. Reads were aligned to the GRCm38 mouse reference genome (Mus_musculus.GRCm38.dna.primary assembly.fa, Ensembl GTF annotation v99). We used STAR version 2.7.3a[27] with the --twopassMode Basic parameter. The STAR index was built against Mus_musculus GRCm38 v99 Ensembl GTF using the option -sjdbOverhang 75. We then used featureCounts version 2.0.0[28] to obtain a count matrix. Genes with less than 5 counts in more than 33% of samples were filtered out. The counts were normalized using DESEQ2's median of ratios method[29]. Differential gene expression and log2 fold changes were obtained using the DESEQ2 package[29] with SVA correction[30]. The default DESEQ2 adjusted p-value threshold of 0.10 was used to identify significant differences between wildtype and mutant samples. The number of differentially expressed genes (DEG) in each tissue was considered as the union of DEG in both *Kdm5b*$^{+/-}$ and *Kdm5b*$^{-/-}$ animals.

For the identification of functionally enriched terms in the differentially expressed genes, Gene Ontology (GO) enrichment analysis was performed using the gprofiler R package (gost function, ordered_query = FALSE). A threshold of 5% FDR and an enrichment significance threshold of P<0.05 (correction_method = "fdr" for multiple testing) was used. In all analyses, the background consisted of only the genes considered expressed in the tissue studied (genes that passed the minimum count filtering that had adjusted p-value with a numerical value, different to NA). GO terms with more than 1,000 genes were excluded from the analysis. The European Nucleotide Archive accession numbers for the RNA-seq sequences reported in this paper are as listed in Supplementary Table 17.

Temporal expression of cognitive function genes

We obtained temporal RNA-seq expression data from BrainSpan[31], an atlas of the developmental human brain. This data was generated from 42 individual donors, across 26 brain regions and in 31 developmental ages, with 524 samples in total. Gene expression was originally processed as reads per kilobase per million (RPKM). We first removed genes that were not expressed (RPKM<1) in more than 10% of the total samples, resulting in 11,744 genes with expression information available. Then, RPKM was transformed to $\log_2(\text{RPKM}+10^{-8})$ (adding 10^{-8} to avoid possible numerical error in logarithm transformation). Thirty-one developmental ages were grouped into 8 developmental stages, early prenatal (8-12 pcw), early mid-prenatal (13-18 pcw), late mid-prenatal (19-24 pcw), late prenatal (25-38 pcw), infancy (0-18 months), childhood (19

months - 11 years), adolescence (12-19 years) and adulthood (20-60+ years). Temporal expression of the cognitive function genes across development stages was fitted loess regression. We compared prenatal and postnatal expression of the cognitive function genes across the QCed dataset with a two-sided two-sample student's *t*-test. We also performed one-way ANOVA to test if the means of different developmental stages were significantly different. A post-hoc Tukey multiple pairwise-comparison between the means of stages was conducted if one-way ANOVA showed significant results.

Supplementary note

Brief summary of cognitive function-associated genes identified

ADGRB2. *ADGRB2* (adhesion G protein-coupled receptor B2; also known as *BAI2*) encodes an adhesion G protein coupled receptor (GPCR) that is one of the main mediators of signal transduction in the central nervous system. ADGRB2 is considered as an orphan GPCR (oGPCR), for which endogenous ligands have not yet been identified[32]. *ADGRB2* is primarily expressed in the brain (neurons and astrocytes in hippocampus, amygdala and cerebral cortex)[33,34]. Variants near *ADGRB2* have been associated with educational attainment in a genome-wide association study[2], and also found associated with other traits such as body mass index[35], smoking, Intraocular pressure[36], or parental longevity[37].

KDM5B. *KDM5B* (lysine demethylase 5B; also known as *JARID1B* or *PLU1*) encodes a lysine-specific histone demethylase in the jumonji/ARID domain-containing family of histone demethylases. The encoded protein can demethylate tri-, di- and monomethylated lysine 4 of histone H3 (H3K4me1/2/3)[38–40], which is broadly associated with enhancers and promoters of actively transcribed genomic loci. Mutations in *KDM5B* are the cause for an autosomal-recessive intellectual disability syndrome[41] (OMIM # 618109) and have further been found associated with schizophrenia[42] and autism spectrum disorder[43,44] in sequencing studies, where disrupted neuronal differentiation was suggested as a potential mechanism. A search on GWAS Catalog (https://www.ebi.ac.uk/gwas/; accessed on Feb. 6, 2022) did not identify significant associations of *KDM5B* variants in previous GWAS. However, we note that the association of *KDM5B* with RT may be influenced by its association with reduced handgrip strength we observed in UKB, which might contribute to the epidemiological observation in UKB that hand grip strength and cognitive function share common mechanisms[45].

GIGYF1. *GIGYF1* (GRB10 interacting GYF protein 1) encodes an adaptor protein (a member of the gyf family) that binds growth factor receptor-bound 10 (GRB10), which in turn binds activated insulin receptors and insulin-like growth factor-1 (IGF-1) receptors[46,47]. By influencing the insulin and IGF-1 signaling pathway, *GIGYF1* plays a role in metabolic diseases and related anthropometric traits. For instance, significant associations were identified in previous GWAS for hemoglobin[48], total cholesterol, low density lipoprotein cholesterol, glucose and apolipoprotein B levels[49]. *GIGYF1* was also associated with mosaic loss of chromosome Y (LOY)[50] and metabolic diseases including glucose and HbA1c levels and type 2 diabetes[51] in previous exome sequencing studies.

ANKRD12. *ANKRD12* (ankyrin repeat domain 12; also known as *ANCO-2*) encodes a member of the ankyrin repeats-containing cofactor (ANCO) family. ANCOs are transcriptional co-regulators that interact with both co-activators and co-repressors[52]. *ANKRD12* interacts with the p160 co-activators (by recruiting HDACs [histone deacetylases]) and the co-activator ADA3

(alteration/deficiency in activation 3)[52,53]. *ANKRD12* was found to be associated with corpuscular measures in GWAS[35,54].

SLC8A1. *SLC8A1* (solute carrier family 8 member A1; also known as *NC*X1) encodes a bidirectional calcium transporter, the cardiac sarcolemmal Na(+)-Ca(2+) exchanger, which is the primary mechanism for cardiac myocyte returning to its resting state following excitation (through extrusion of calcium) and plays a critical role in cardiac contractility[55]. *SLC8A1* expression is enriched in human heart tissue. *SLC8A1* has been shown to be associated with bone mineral density[56], blood pressure[57], blood biomarkers (for example IGF-1 [49]), electrocardiographic traits (PR interval[58], QT interval[59], etc.) and hand grip strength[60] among others.

RC3H2. *RC3H2* (ring finger and CCCH-type domains 2) encodes roquin-2 that belongs to a family of highly conserved RNA-binding proteins (roquins) that regulate their target genes on the post-transcriptional level. Roquins contain a RING (Really Interesting New Gene)-type E3 ubiquitin ligase domain, followed by a ROQ domain and a CCCH-type ZnF domain[61–63]. Roquins play key roles in maintaining peripheral immunological tolerance and autoimmune diseases[64]. It has been shown that *RC3H2* (and *RC3H1*) restricts T-cell activation and costimulation via *ICOS* and *OX40* to prevent inappropriate Tfh cell differentiation[65]. Roquin-2 is widely expressed in all human tissues. *RC3H2* showed genome-wide significant association with insomnia[66] and HbA1c[35] in GWAS.

CACNA1A. *CACNA1A* (calcium voltage-gated channel subunit alpha1 A) encodes the alpha-1A subunit of the voltage-dependent calcium channels. It is primarily expressed in neuronal tissue. Mutations in *CACNA1A* are a cause for type 2 episodic ataxia (OMIM #108500), spinocerebellar ataxia 6 (OMIM #183086), developmental and epileptic encephalopathy 42 (OMIM #617106) and familial hemiplegic migraine (OMIM #141500). *CACNA1A* was implicated in a previous educational attainment GWAS[2], but the top associated SNP and LD peak do not fall into the *CACNA1A* gene region, but rather located in the intergenic region between *CACNA1A* and *RPL12P42*. Other GWAS associations for *CACNA1A* include depressive symptoms[67], age at first birth[68] and brain region volume[69].

BCAS3. *BCAS3* (BCAS3 microtubule associated cell migration factor) encodes a large, highly conserved cytoskeletal protein involved in human embryogenesis and tumor angiogenesis[70,71]. It has recently been shown that *BCAS3* loss-of-function variants can cause Hengel-Maroofian-Schols syndrome (HEMARS; OMIM #619641), which is an autosomal recessive neurodevelopmental disorder characterized by severe global developmental delay starting from infancy or early childhood with facial dysmorphism and brain abnormalities [71]. *BCAS3* has also been associated with glomerular filtration rate[72], bone mineral density[56], serum creatinine level[35],

hemoglobin concentration[54], serum urate level[73], red blood cell count[35], ophthalmologic measures (e.g. macular thickness[74]), coronary artery disease[75] and additional traits in GWAS.

Estimating burden heritability for rare variants with Burden Heritability Regression

We used a new method, burden heritability regression (BHR)[76], to estimate the phenotypic variance explained (burden heritability) by the gene-wise burden of rare coding variants. Using exome sequencing data in the UK Biobank European samples, we performed single variant association tests for educational attainment (EDU), reaction time (RT) and verbal-numerical reasoning (VNR) with PTVs and missense variants with a minor allele count greater than 5. The association tests were done using two-step whole genome regression implemented in Regenie (version 3.0.3) with the same model set up as described in the Method section "Exome-wide gene-based PTV burden test"). We estimated burden heritability for PTV and missense variants separately following the default settings of BHR with provided baseline model (https://github.com/ajaynadig/bhr) for 3 different minor allele frequency categories for the burden: [0, 0.0001), [0, 0.001), and [0, 0.01).

The burden heritability estimates are shown in the table below. In our analysis, the burden heritability for EDU, RT, and VNR ranged from 0.0025 to 0 for PTV and missense burdens separately with MAF cut-off from 0.0001 to 0.01. From Weiner et al., the burden heritability was estimated as 0.0191 (SE=0.00283) for VNR and 0.0063 (SE=0.00091) for RT by combining PTV and missense variants with MAF<0.001. Compared with the burden heritability estimates for RT and VNR presented in the BHR method paper by Weiner et al., our burden heritability estimates appear to be lower, albeit the two sets of results (our current study and Weiner et al.) are not directly comparable due to different data curation, annotation, and statistical analysis set-up (e.g., different allele frequency and annotation categories were used). In particular, we performed single variant association tests using two-step whole genome regression implemented in Regenie and followed the recommendation of the method developer to exclude all variants with minor allele count (MAC) less than 5 (approximately MAF<$7x10^{-6}$) to ensure stable estimation for single variant association statistics. On the other hand, Weiner et al. used single variant association test statistics obtained from the Genebass Browser (https://app.genebass.org/)[10], which were generated with a mixed model association test implemented in SAIGE-GENE and included all single variants with MAC>0. As shown in Weiner et al., most burden heritability is explained by ultra-rare loss-of-function variants with MAF between 10^{-6} to 10^{-5}, which we mostly excluded from our burden heritability regression analyses. This may potentially explain lower burden heritability estimates in our analyses. Notably, a common theme is that the burden heritability of rare PTVs and missense variants are much lower than common variant heritability for cognitive function. The common variant-based heritability from previous GWAS were 0.15 for EDU[2], 0.07 for RT[6], and 0.25 for VNR[6]. These results support the proposed flattening hypothesis[77], where it hypothesized that only a limited number of genes are critical for most of the human complex phenotypes and negative selection is gradually removing such variants with large effects in critical genes from the population (hence they are rare) and leaves behind a lot more common variants with small effects in less critical genes. This phenomenon may lead to the differences we observe between heritability for common variants with small effect versus rare coding variants with large effects. On the other hand, this hypothesis also explains why genes identified in rare variant association studies with large effect sizes are of higher biological relevance even with a smaller number of people in the population affected by genetic variants in these genes.

Burden heritability estimates for educational attainment, reaction time and verbal-numerical reasoning in UK Biobank European samples

Phenotype	Variant category	Burden MAF cut-off (max)	Bruden heritability	SE
Educational attainment	PTV	0.0001	-0.0002	0.00027
Educational attainment	missense	0.0001	0.0011	0.00024
Educational attainment	PTV	0.001	0.0004	0.00026
Educational attainment	missense	0.001	0.0009	0.00025
Educational attainment	PTV	0.01	0.0008	0.00024
Educational attainment	missense	0.01	0.0025	0.00038
Reaction time	PTV	0.0001	0.0005	0.00035
Reaction time	missense	0.0001	0.0007	0.00031
Reaction time	PTV	0.001	0.0005	0.00038
Reaction time	missense	0.001	0.0011	0.00033
Reaction time	PTV	0.01	0.0004	0.00022
Reaction time	missense	0.01	0.0020	0.00041
Verbal-numerical reasoning (baseline)	PTV	0.0001	-0.0006	0.00075
Verbal-numerical reasoning (baseline)	missense	0.0001	0.0006	0.00082
Verbal-numerical reasoning (baseline)	PTV	0.001	0.0003	0.00068
Verbal-numerical reasoning (baseline)	missense	0.001	0.0025	0.00082
Verbal-numerical reasoning (baseline)	PTV	0.01	0.0021	0.00061
Verbal-numerical reasoning (baseline)	missense	0.01	0.0054	0.00106

Biogen Biobank team

Steering team: Ellen Tsai, Sally John, Heiko Runz

Data management team: Eric Marshall, Mehool Patel, Saranya Duraisamy

Extended Scientific team: Dennis Baird, Danai Chasioti, Chia-Yen Chen, Susan Eaton, Jake Gagnon, Feng Gao, Cynthia Gubbels, Yunfeng Huang, Varant Kupelian, Stephanie Loomis, Helen McLaughlin, Adele Mitchell, Lili Peng, Coro Paisan-Ruiz, Benjamin Sun

Supplementary Figures

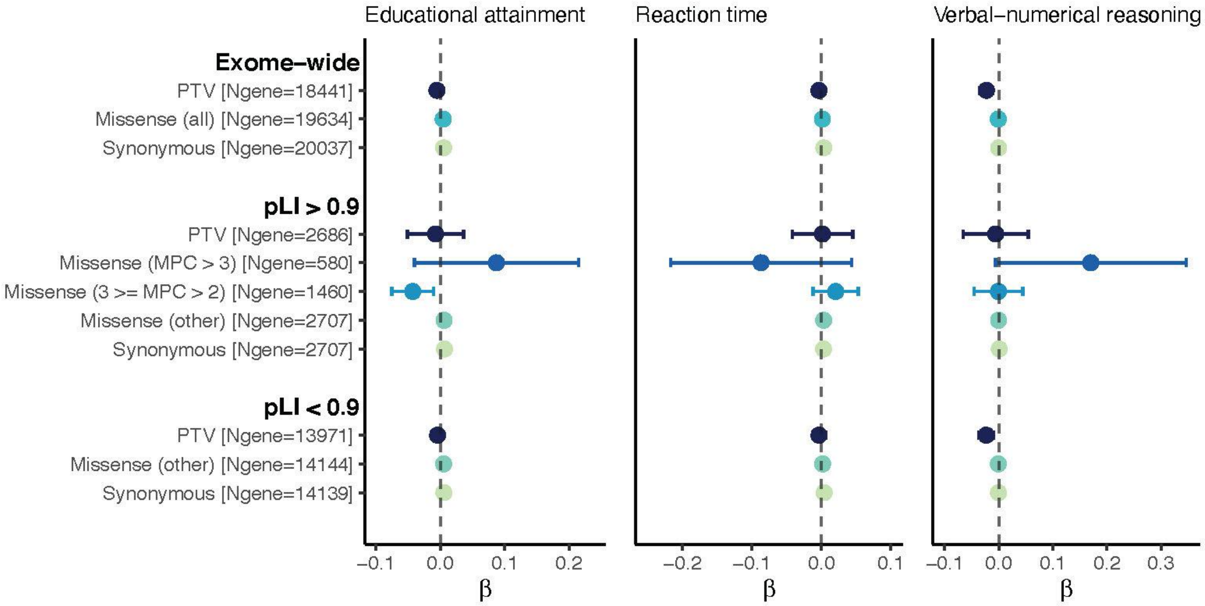

Supplementary Fig. 1. Impact of exome-wide burden of rare protein coding variants on educational attainment (EDU), reaction time (RT) and verbal-numerical reasoning (VNR) in unrelated South Asian (SAS) samples in the UK Biobank (N=6,604 for EDU, 6,483 for RT, and 3,589 for VNR).

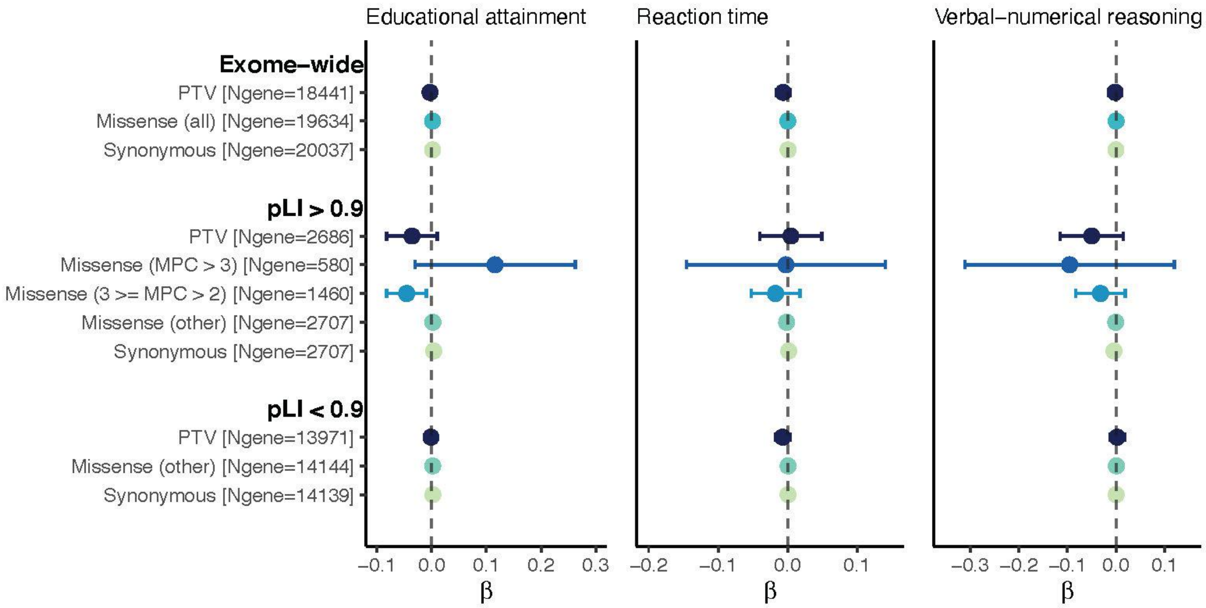

Supplementary Fig. 2. Impact of exome-wide burden of rare protein coding variants on educational attainment (EDU), reaction time (RT) and verbal-numerical reasoning (VNR) in African samples (AFR) in the UK Biobank (N=6,065 for EDU, 5,931 for RT, and 3,149 for VNR).

a

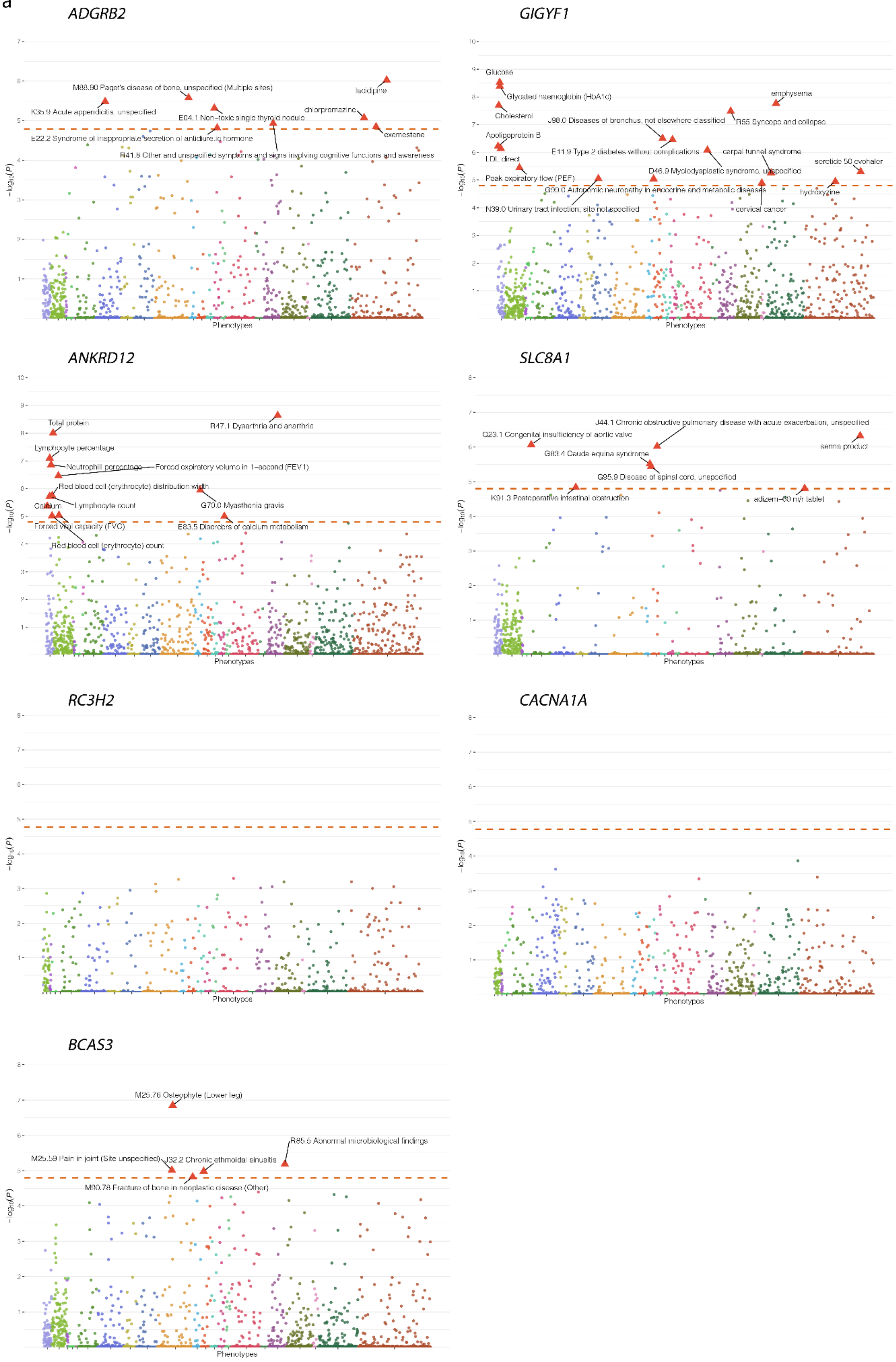
ADGRB2
M88.90 Paget's disease of bone, unspecified (Multiple sites)
lacidipine
K35.9 Acute appendicitis, unspecified
chlorpromazine
E04.1 Non-toxic single thyroid nodule
exemestane
E22.2 Syndrome of inappropriate secretion of antidiuretic hormone
R41.8 Other and unspecified symptoms and signs involving cognitive functions and awareness
−log10(P)
Phenotypes
GIGYF1
Glucose
Glycated haemoglobin (HbA1c)
emphysema
Cholesterol
J98.0 Diseases of bronchus, not elsewhere classified
R55 Syncope and collapse
Apolipoprotein B
E11.9 Type 2 diabetes without complications
carpal tunnel syndrome
LDL direct
secretide 50 cyclohaler
D46.9 Myelodysplastic syndrome, unspecified
Peak expiratory flow (PEF)
G99.0 Autonomic neuropathy in endocrine and metabolic diseases
hydroxyzine
N39.0 Urinary tract infection, site not specified
cervical cancer
−log10(P)
Phenotypes
ANKRD12
Total protein
R47.1 Dysarthria and anarthria
Lymphocyte percentage
Neutrophill percentage
Forced expiratory volume in 1-second (FEV1)
Red blood cell (erythrocyte) distribution width
Calcium
Lymphocyte count
G70.0 Myasthenia gravis
Forced vital capacity (FVC)
E83.5 Disorders of calcium metabolism
Red blood cell (erythrocyte) count
−log10(P)
Phenotypes
SLC8A1
J44.1 Chronic obstructive pulmonary disease with acute exacerbation, unspecified
Q23.1 Congenital insufficiency of aortic valve
senna product
G83.4 Cauda equina syndrome
G95.9 Disease of spinal cord, unspecified
K91.3 Postoperative intestinal obstruction
adizem-60 m/r tablet
−log10(P)
Phenotypes
RC3H2
−log10(P)
Phenotypes
CACNA1A
−log10(P)
Phenotypes
BCAS3
M25.76 Osteophyte (Lower leg)
R85.5 Abnormal microbiological findings
M25.59 Pain in joint (Site unspecified)
J32.2 Chronic ethmoidal sinusitis
M80.78 Fracture of bone in neoplastic disease (Other)
−log10(P)
Phenotypes

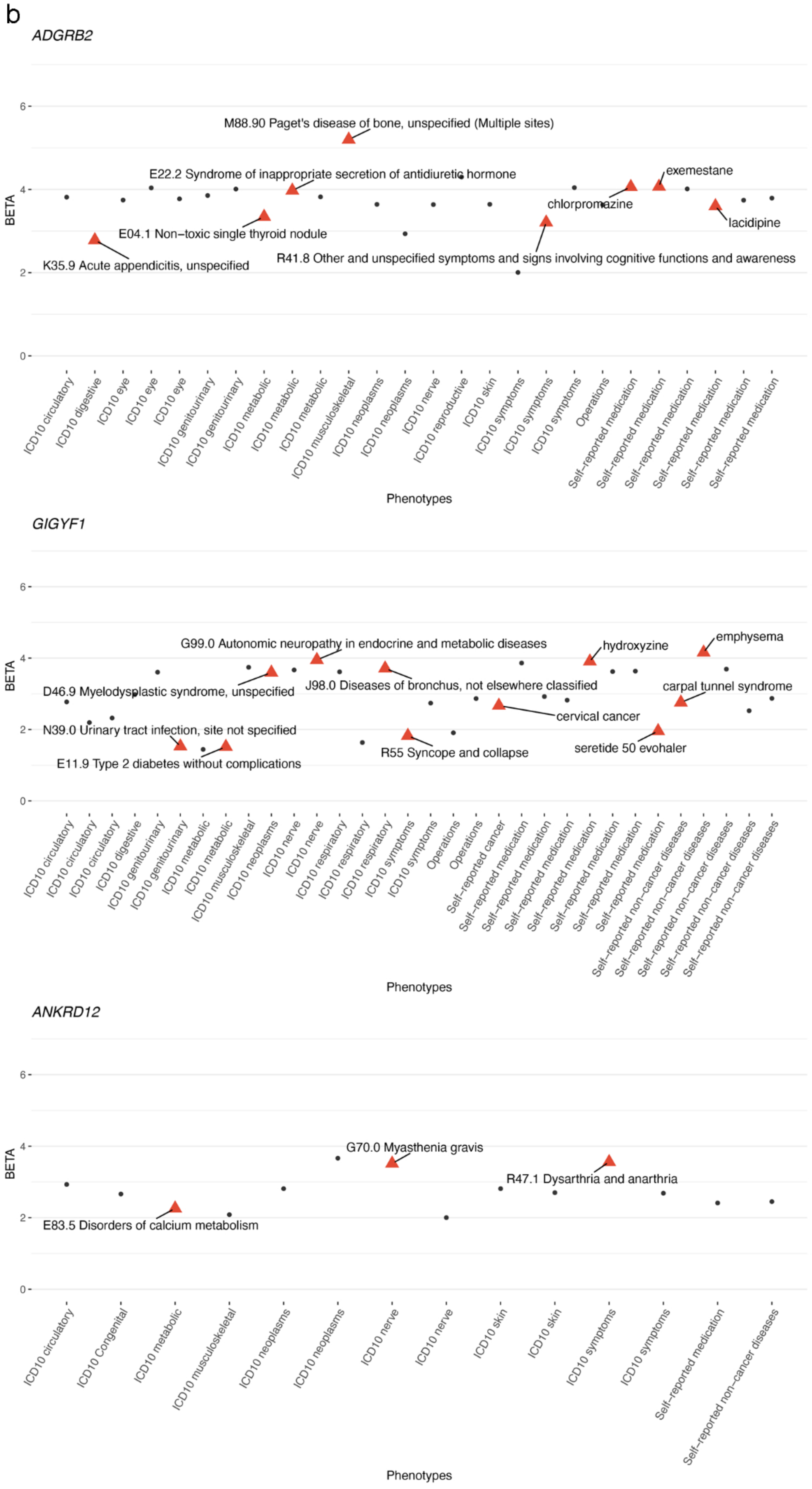
b
ADGRB2
BETA
M88.90 Paget's disease of bone, unspecified (Multiple sites)
E22.2 Syndrome of inappropriate secretion of antidiuretic hormone
exemestane
chlorpromazine
E04.1 Non−toxic single thyroid nodule
lacidipine
K35.9 Acute appendicitis, unspecified
R41.8 Other and unspecified symptoms and signs involving cognitive functions and awareness
ICD10 circulatory
ICD10 digestive
ICD10 eye
ICD10 eye
ICD10 eye
ICD10 genitourinary
ICD10 genitourinary
ICD10 metabolic
ICD10 metabolic
ICD10 metabolic
ICD10 musculoskeletal
ICD10 neoplasms
ICD10 neoplasms
ICD10 nerve
ICD10 reproductive
ICD10 skin
ICD10 symptoms
ICD10 symptoms
ICD10 symptoms
Operations
Self−reported medication
Self−reported medication
Self−reported medication
Self−reported medication
Self−reported medication
Self−reported medication
Phenotypes
GIGYF1
BETA
G99.0 Autonomic neuropathy in endocrine and metabolic diseases
hydroxyzine
emphysema
D46.9 Myelodysplastic syndrome, unspecified
J98.0 Diseases of bronchus, not elsewhere classified
carpal tunnel syndrome
cervical cancer
N39.0 Urinary tract infection, site not specified
seretide 50 evohaler
R55 Syncope and collapse
E11.9 Type 2 diabetes without complications
ICD10 circulatory
ICD10 circulatory
ICD10 circulatory
ICD10 digestive
ICD10 genitourinary
ICD10 genitourinary
ICD10 metabolic
ICD10 metabolic
ICD10 musculoskeletal
ICD10 neoplasms
ICD10 nerve
ICD10 nerve
ICD10 respiratory
ICD10 respiratory
ICD10 respiratory
ICD10 symptoms
ICD10 symptoms
Operations
Operations
Self−reported cancer
Self−reported medication
Self−reported medication
Self−reported medication
Self−reported medication
Self−reported medication
Self−reported medication
Self−reported medication
Self−reported non−cancer diseases
Self−reported non−cancer diseases
Self−reported non−cancer diseases
Self−reported non−cancer diseases
Self−reported non−cancer diseases
Phenotypes
ANKRD12
BETA
G70.0 Myasthenia gravis
R47.1 Dysarthria and anarthria
E83.5 Disorders of calcium metabolism
ICD10 circulatory
ICD10 Congenital
ICD10 metabolic
ICD10 musculoskeletal
ICD10 neoplasms
ICD10 neoplasms
ICD10 nerve
ICD10 nerve
ICD10 skin
ICD10 skin
ICD10 symptoms
ICD10 symptoms
Self−reported medication
Self−reported non−cancer diseases
Phenotypes

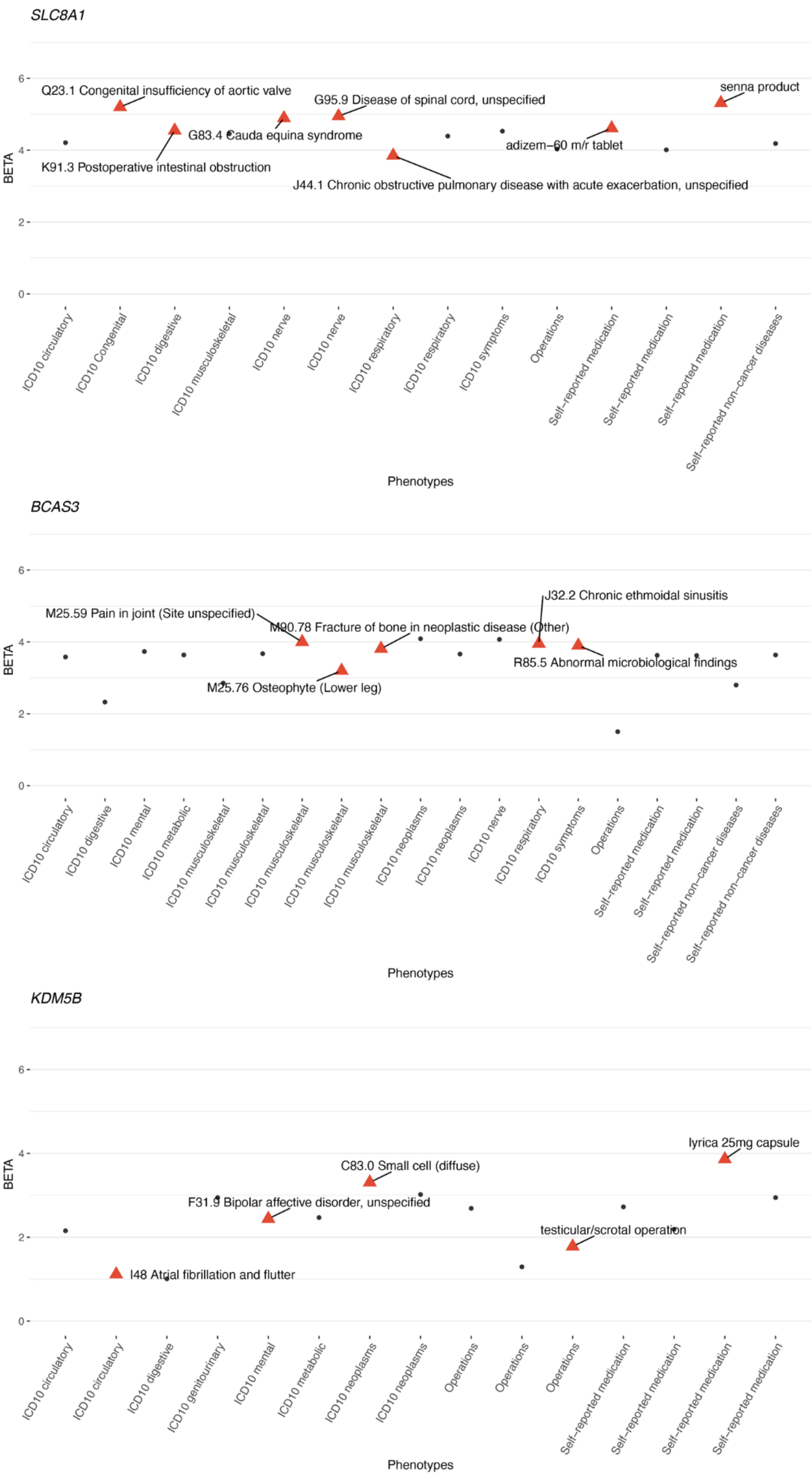
SLC8A1
BETA
Q23.1 Congenital insufficiency of aortic valve
G95.9 Disease of spinal cord, unspecified
senna product
G83.4 Cauda equina syndrome
adizem−60 m/r tablet
K91.3 Postoperative intestinal obstruction
J44.1 Chronic obstructive pulmonary disease with acute exacerbation, unspecified
ICD10 circulatory
ICD10 Congenital
ICD10 digestive
ICD10 musculoskeletal
ICD10 nerve
ICD10 nerve
ICD10 respiratory
ICD10 respiratory
ICD10 symptoms
Operations
Self−reported medication
Self−reported medication
Self−reported medication
Self−reported non−cancer diseases
Phenotypes
BCAS3
BETA
J32.2 Chronic ethmoidal sinusitis
M25.59 Pain in joint (Site unspecified)
M90.78 Fracture of bone in neoplastic disease (Other)
R85.5 Abnormal microbiological findings
M25.76 Osteophyte (Lower leg)
ICD10 circulatory
ICD10 digestive
ICD10 mental
ICD10 metabolic
ICD10 musculoskeletal
ICD10 musculoskeletal
ICD10 musculoskeletal
ICD10 musculoskeletal
ICD10 musculoskeletal
ICD10 neoplasms
ICD10 neoplasms
ICD10 nerve
ICD10 respiratory
ICD10 symptoms
Operations
Self−reported medication
Self−reported medication
Self−reported non−cancer diseases
Self−reported non−cancer diseases
Phenotypes
KDM5B
BETA
lyrica 25mg capsule
C83.0 Small cell (diffuse)
F31.9 Bipolar affective disorder, unspecified
testicular/scrotal operation
I48 Atrial fibrillation and flutter
ICD10 circulatory
ICD10 circulatory
ICD10 digestive
ICD10 genitourinary
ICD10 mental
ICD10 metabolic
ICD10 neoplasms
ICD10 neoplasms
Operations
Operations
Operations
Self−reported medication
Self−reported medication
Self−reported medication
Self−reported medication
Phenotypes

Supplementary Fig. 3. Phenome-wide association analysis (3,150 phenotypes) for *ADGRB2*, *GIGYF1*, *ANKRD12*, *SLC8A1*, *RC3H2, CACNA1A* and *BCAS3* in unrelated European samples in the UK Biobank.
The full PheWAS results can be found in Supplementary Table 9.
a. PheWAS association p-values were plotted for each phenotype. Orange dashed line represents the Bonferroni corrected phenome-wide significance p-value (two-sided *t*-test) threshold ($0.05/3150=1.59\times10^{-5}$). Phenotypes were grouped and color-coded from left to right in the following categories: biomarker; composite phenotypes; family history; ICD-10 cause of death, ICD-10 congenital malformations; deformations and chromosomal abnormalities; ICD-10 diseases of the circulatory system; ICD-10 diseases of the digestive system; ICD-10 diseases of the eye and adnexa; ICD-10 diseases of the genitourinary system; ICD-10 diseases of the musculoskeletal system and connective tissue; ICD-10 diseases of the nervous system; ICD-10 diseases of the respiratory system; ICD-10 diseases of the skin and subcutaneous tissue; ICD-10 endocrine, nutritional and metabolic diseases; ICD-10 mental, behavioral and neurodevelopmental disorders; ICD-10 neoplasms; ICD-10 pregnancy, childbirth and the puerperium; ICD-10 symptoms, signs and abnormal clinical and laboratory findings, not elsewhere classified; operation code; self-reported illness: cancer; self-reported illness: non−cancer; self-reported medication.
b. PheWAS association effect sizes (β) were plotted for disease phenotypes with association p-value less than 0.0001. Phenotypes with phenome-wide significant association p-values were labeled. Other phenotypes were represented by the phenotype categories on x-axis. Triangles represent phenome-wide significant associations.

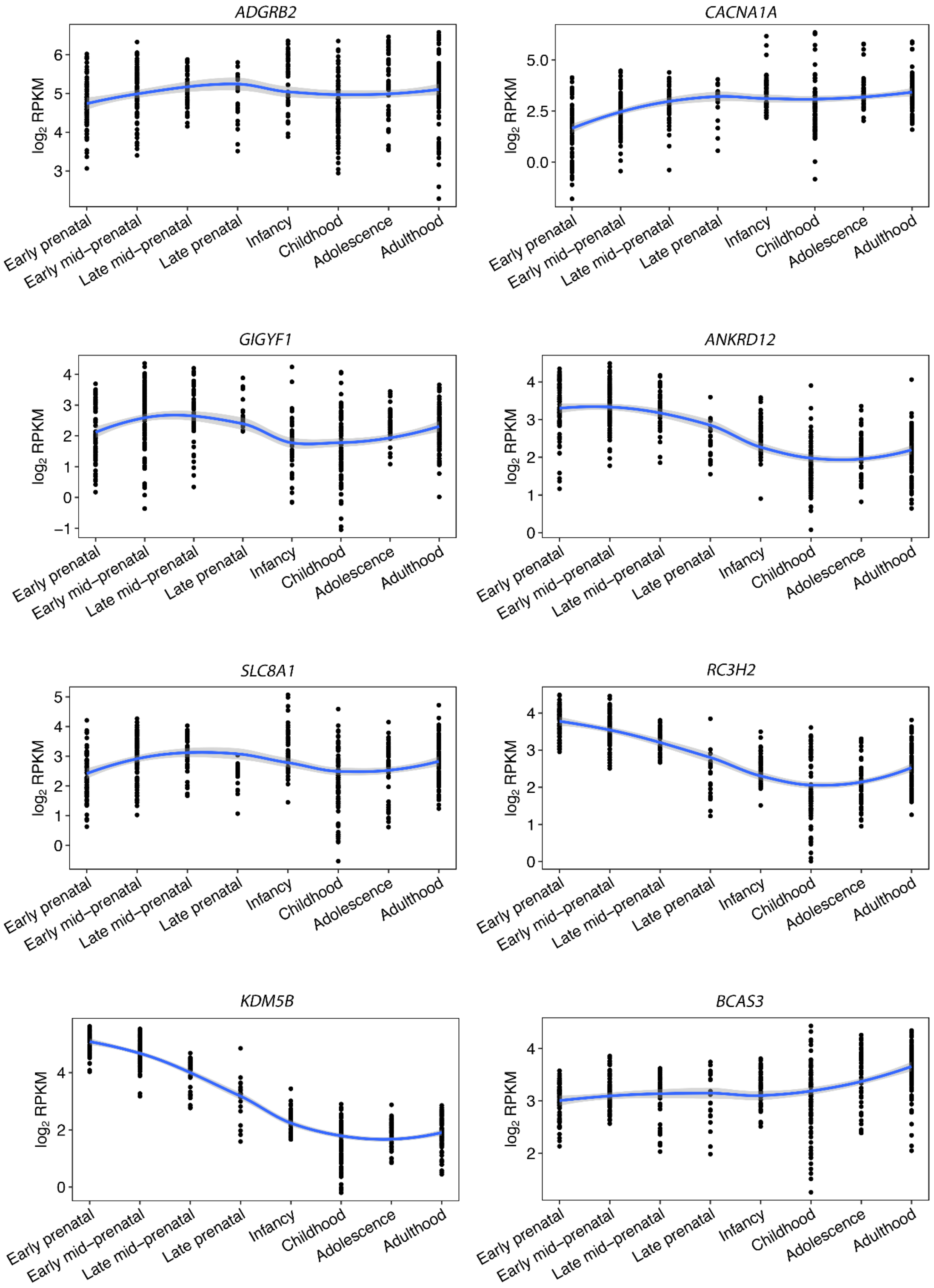

Supplementary Fig. 4. Cognitive function gene expression in brain tissue at different developmental stages. RNA-seq data obtained from BrainSpan[31].
The blue line represents fitted loess regression on *KDM5B* expression cross development stages. The gray band represents 95% confidence intervals for the fitted loess regression.

E18.5

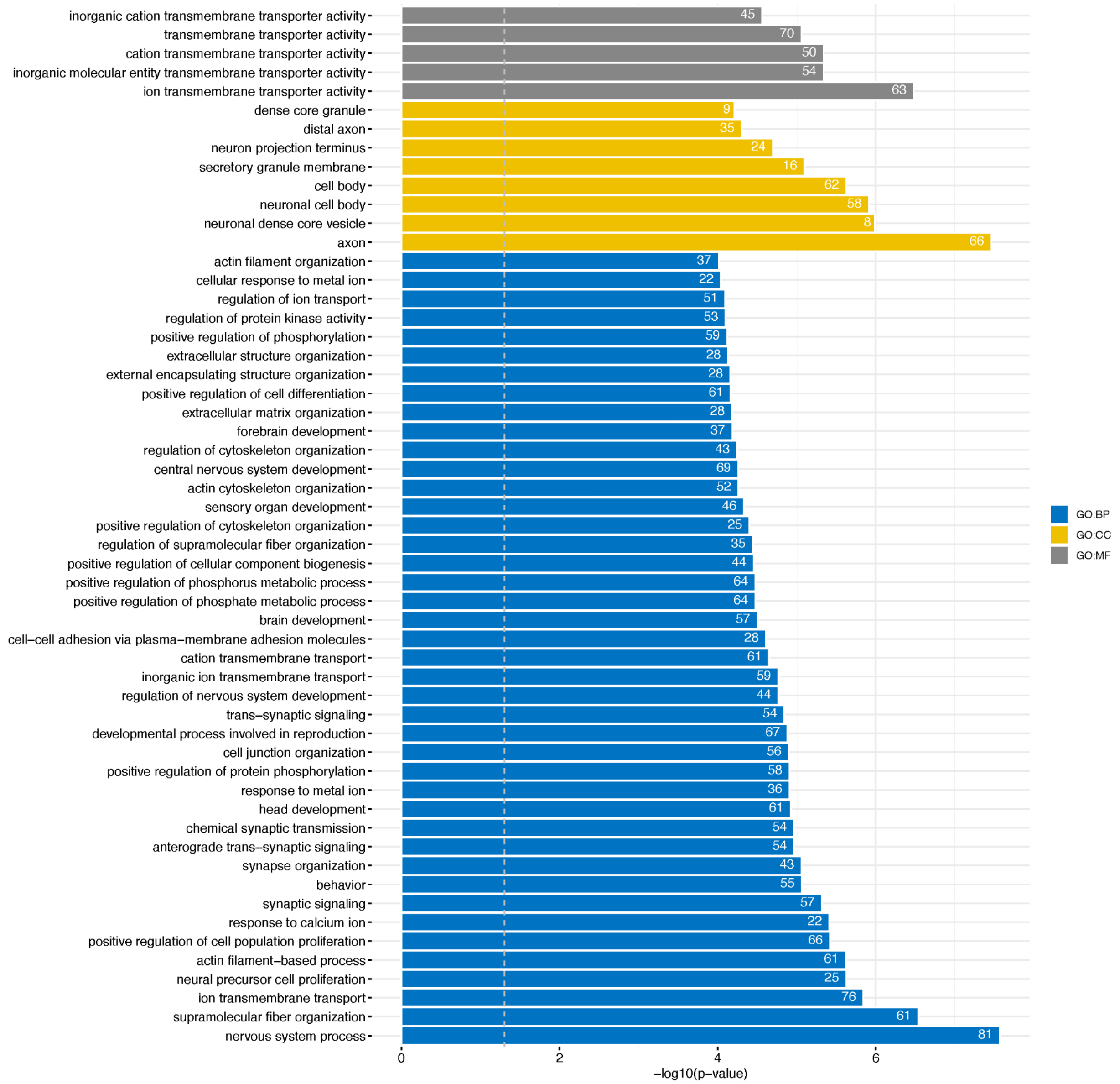
inorganic cation transmembrane transporter activity
transmembrane transporter activity
cation transmembrane transporter activity
inorganic molecular entity transmembrane transporter activity
ion transmembrane transporter activity
dense core granule
distal axon
neuron projection terminus
secretory granule membrane
cell body
neuronal cell body
neuronal dense core vesicle
axon
actin filament organization
cellular response to metal ion
regulation of ion transport
regulation of protein kinase activity
positive regulation of phosphorylation
extracellular structure organization
external encapsulating structure organization
positive regulation of cell differentiation
extracellular matrix organization
forebrain development
regulation of cytoskeleton organization
central nervous system development
actin cytoskeleton organization
sensory organ development
positive regulation of cytoskeleton organization
regulation of supramolecular fiber organization
positive regulation of cellular component biogenesis
positive regulation of phosphorus metabolic process
positive regulation of phosphate metabolic process
brain development
cell−cell adhesion via plasma−membrane adhesion molecules
cation transmembrane transport
inorganic ion transmembrane transport
regulation of nervous system development
trans−synaptic signaling
developmental process involved in reproduction
cell junction organization
positive regulation of protein phosphorylation
response to metal ion
head development
chemical synaptic transmission
anterograde trans−synaptic signaling
synapse organization
behavior
synaptic signaling
response to calcium ion
positive regulation of cell population proliferation
actin filament−based process
neural precursor cell proliferation
ion transmembrane transport
supramolecular fiber organization
nervous system process
45
70
50
54
63
9
35
24
16
62
58
8
66
37
22
51
53
59
28
28
61
28
37
43
69
52
46
25
35
44
64
64
57
28
61
59
44
54
67
56
58
36
61
54
54
43
55
57
22
66
61
25
76
61
81
0
2
4
6
−log10(p−value)
GO:BP
GO:CC
GO:MF

Adult Frontal Cortex

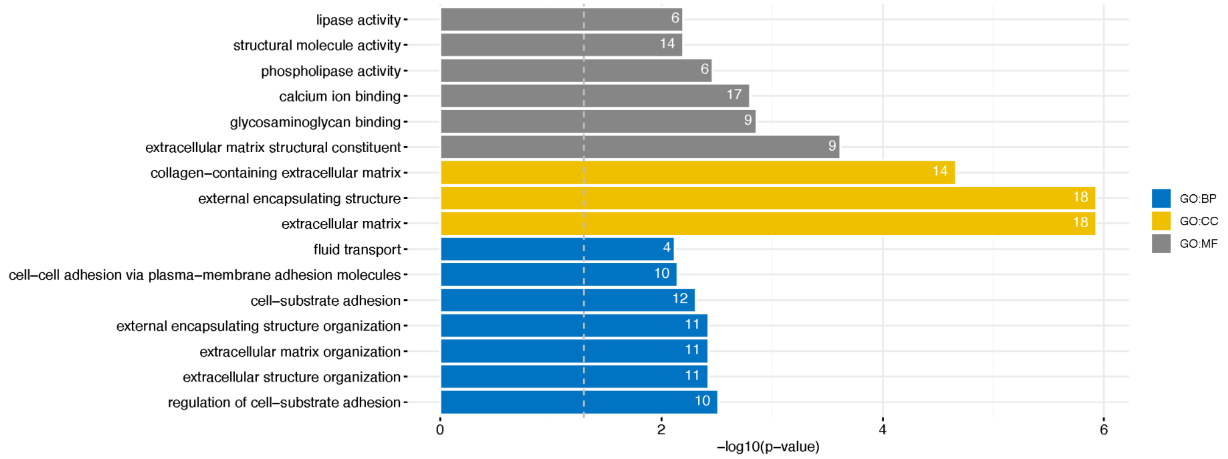

Adult Hippocampus

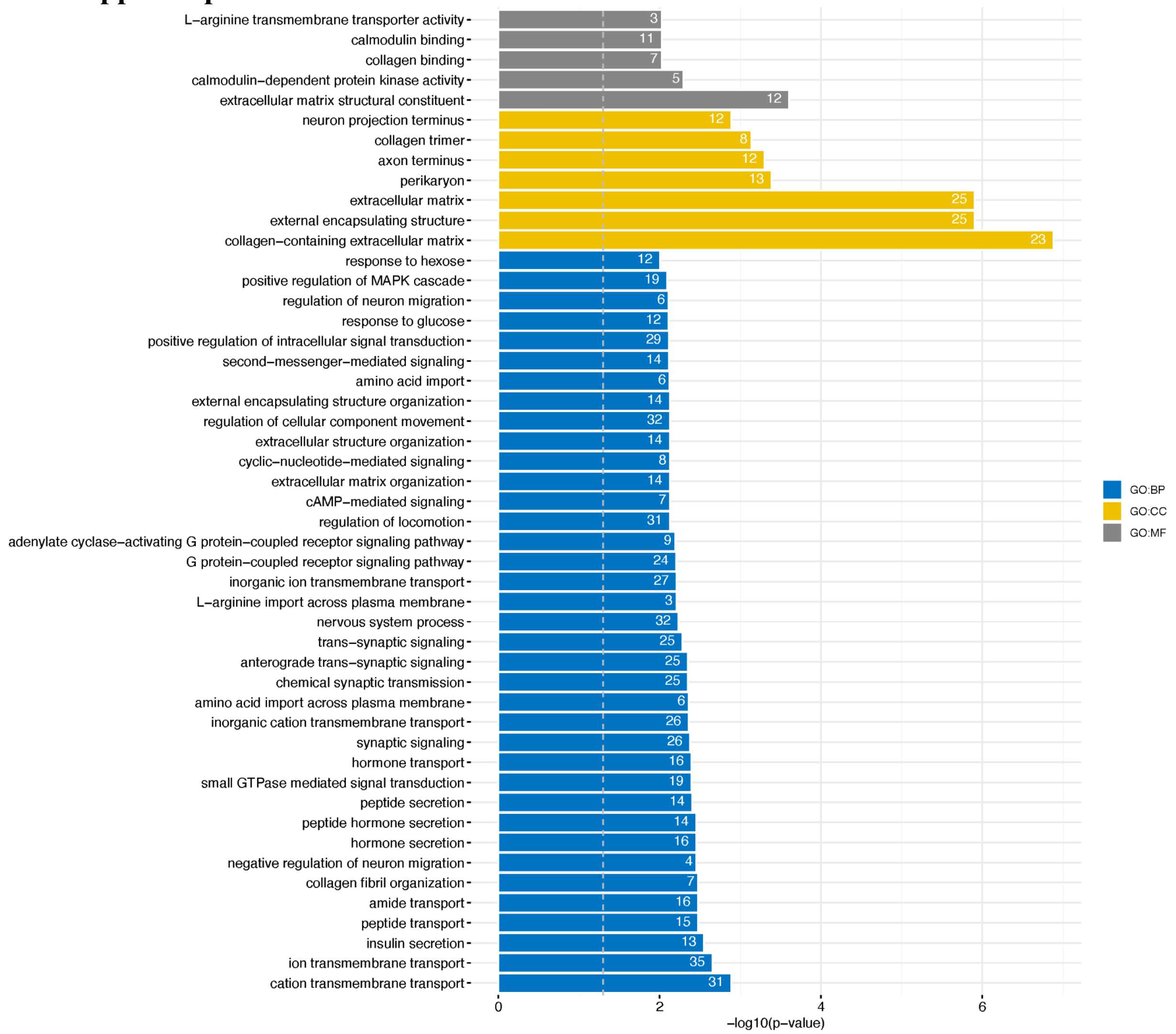

Adult Cerebellum

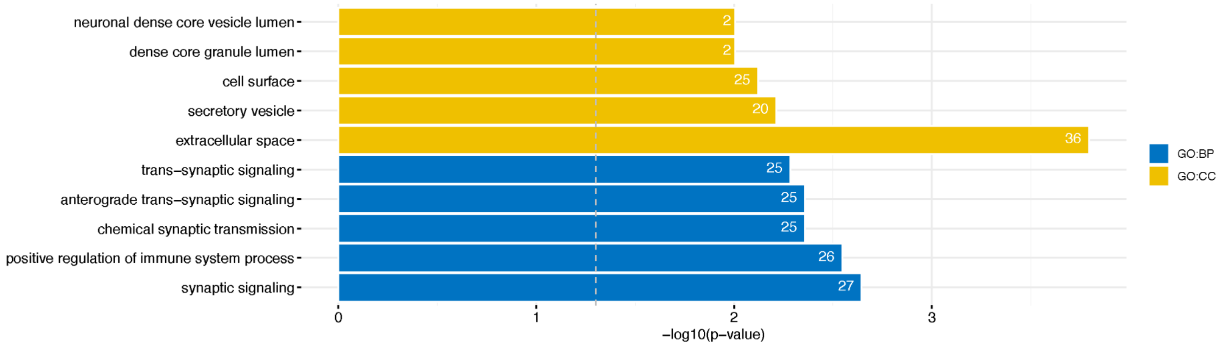

Supplementary Fig. 5. GO term enrichment for differentially expressed genes in *Kdm5b* mutant mice.

Differentially expressed genes (DEGs) from E18.5 and adult brain tissues of *Kdm5b*$^{+/-}$ and *Kdm5b*$^{-/-}$ mice were subject to Gene ontology (GO) pathway enrichment analysis using the gprofiler R package, with a threshold of 5% FDR and an enrichment significance threshold of $P<0.05$ (hypergeometric test with FDR correction for multiple testing). For the E18.5 sample, we only showed results with enrichment p-value<0.0001 (for display purposes). Full results are provided in Supplementary Table 16. The European Nucleotide Archive accession numbers for the RNA-seq sequences reported are provided in Supplementary Table 17. Background comprised only expressed genes in each tissue of interest.

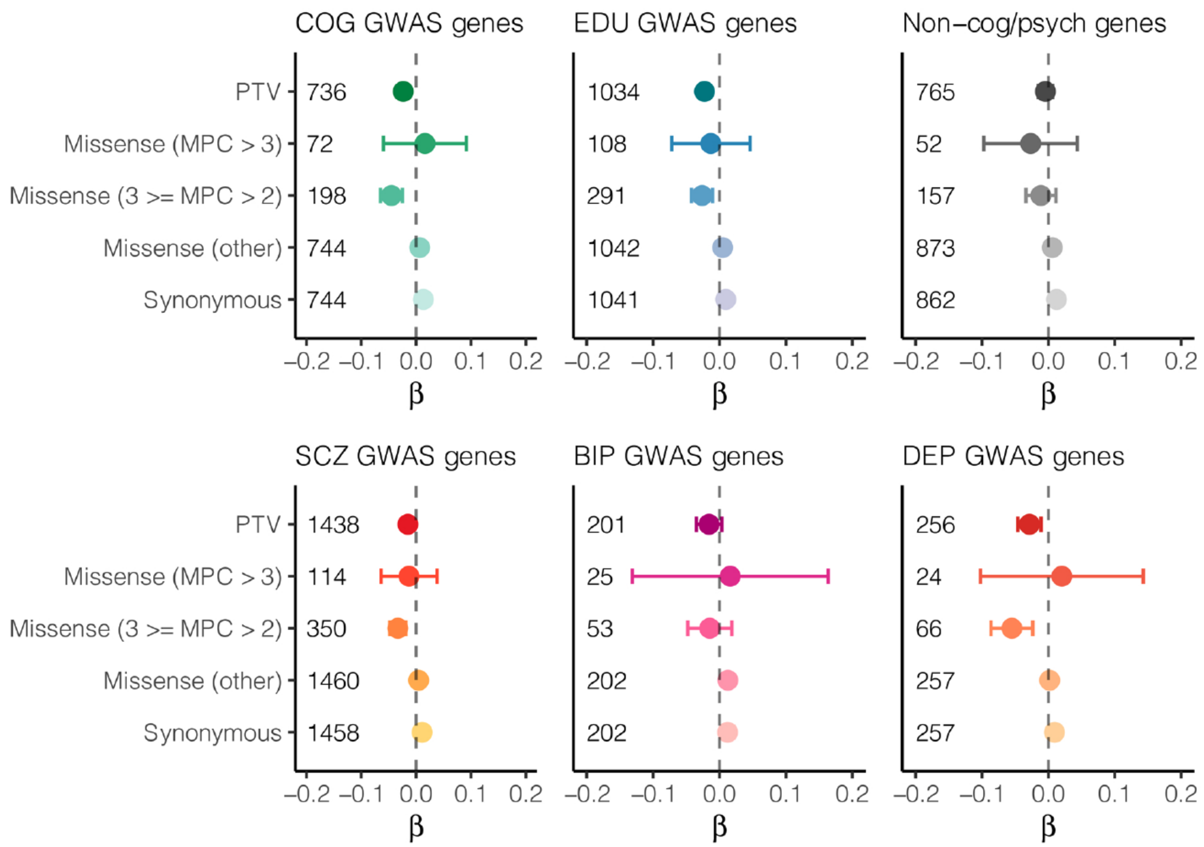

Supplementary Fig. 6. Rare coding variant burden in genes identified in GWAS for cognitive function, educational attainment, schizophrenia, bipolar disorder and depression and non-cognitive function related genes on educational attainment (EDU).
Unrelated UKB EUR samples were included for this analysis (N=318,844). The impact of rare coding variant burden in genes identified through common variant association in GWAS for cognitive function (COG), educational attainment (EDU), schizophrenia (SCZ), bipolar disorder (BIP) and depression (DEP) and in non-cognitive function/non-psychiatric disorder-related (non-cog/psych) genes on EDU. Missense variants were classified by deleteriousness (MPC) into 3 tiers: MPC>3; 3≥MPC>2; and all missense variants not in the previous two tiers. The number of genes included in each burden was labeled in each panel. Data are presented in effect size estimates (β) with 95% confidence intervals.

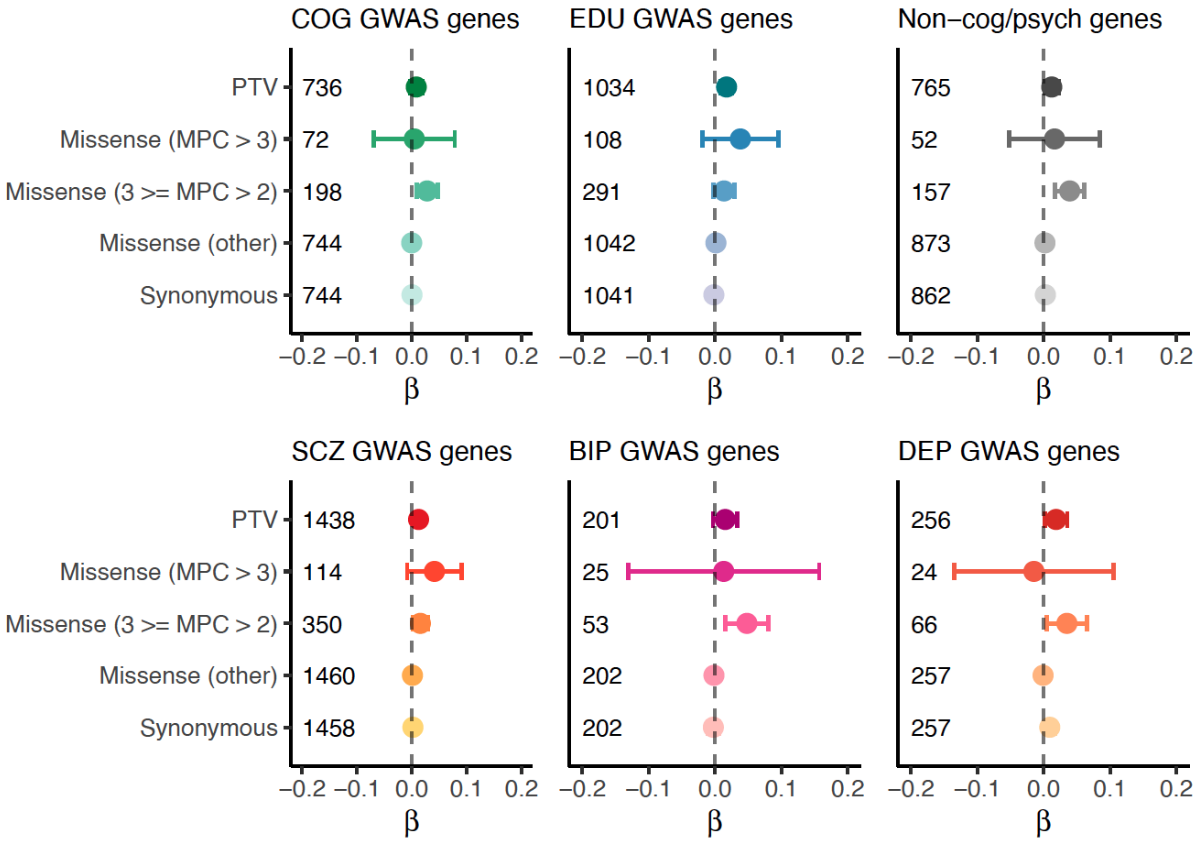

Supplementary Fig. 7. Rare coding variant burden in genes identified in GWAS for cognitive function, educational attainment, schizophrenia, bipolar disorder and depression and non-cognitive function related genes on reaction time (RT).

Unrelated UKB EUR samples were included for this analysis (N=319,536). The impact of rare coding variant burden in genes identified through common variant association in GWAS for cognitive function (COG), educational attainment (EDU), schizophrenia (SCZ), bipolar disorder (BIP) and depression (DEP) and in non-cognitive function/non-psychiatric disorder-related (non-cog/psych) genes on EDU. Missense variants were classified by deleteriousness (MPC) into 3 tiers: MPC>3; 3≥MPC>2; and all missense variants not in the previous two tiers. The number of genes included in each burden was labeled in each panel in Supplementary Fig. 6. Data are presented in effect size estimates (β) with 95% confidence intervals.

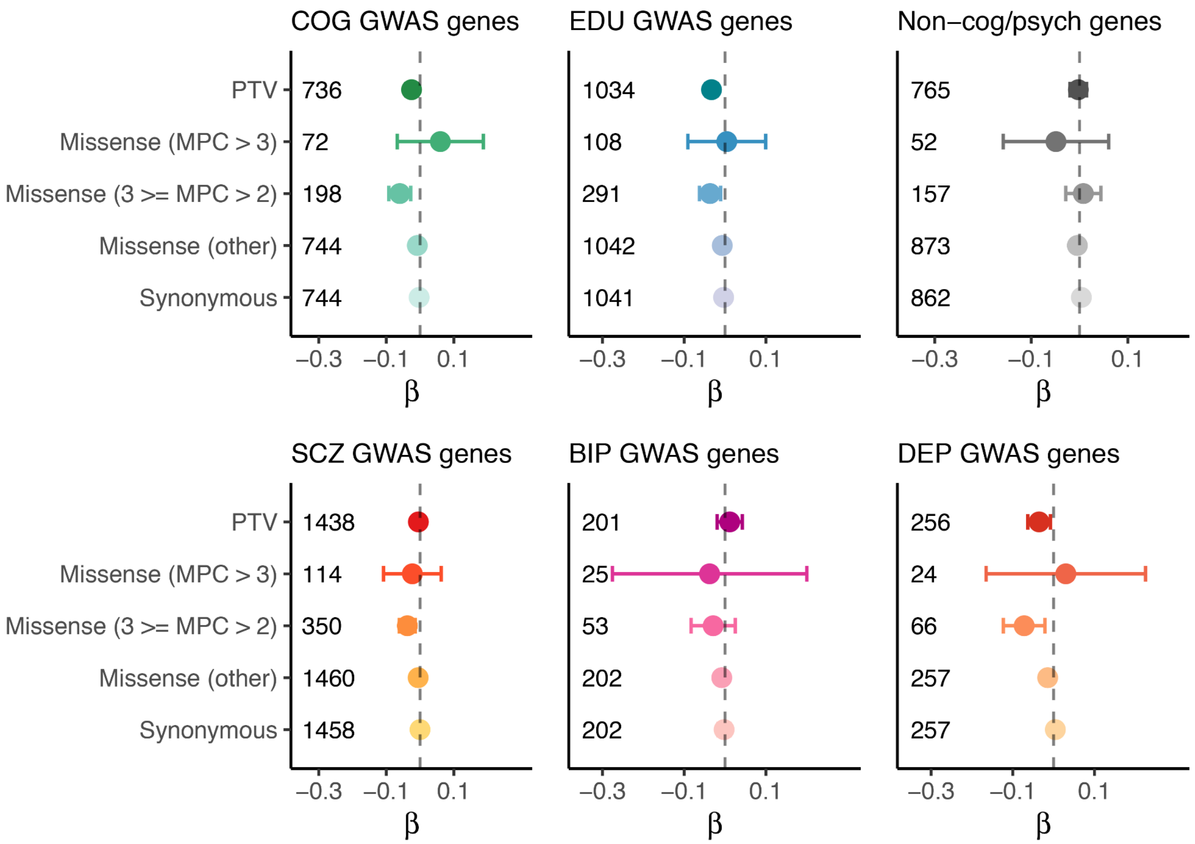

Supplementary Fig. 8. The impact of rare coding variant burdens in genes identified in GWAS for cognitive function, educational attainment, schizophrenia, bipolar disorder and depression and non-cognitive function related genes on verbal-numerical reasoning (VNR). Unrelated UKB EUR samples were included for this analysis (N=128,812). The impact of rare coding variant burden in genes identified through common variant association in GWAS for cognitive function (COG), educational attainment (EDU), schizophrenia (SCZ), bipolar disorder (BIP) and depression (DEP) and in non-cognitive function/non-psychiatric disorder-related (non-cog/psych) genes on EDU. Missense variants were classified by deleteriousness (MPC) into 3 tiers: MPC>3; 3≥MPC>2; and all missense variants not in the previous two tiers. The number of genes included in each burden was labeled in each panel in Supplementary Fig. 6. Data are presented in effect size estimates (β) with 95% confidence intervals.

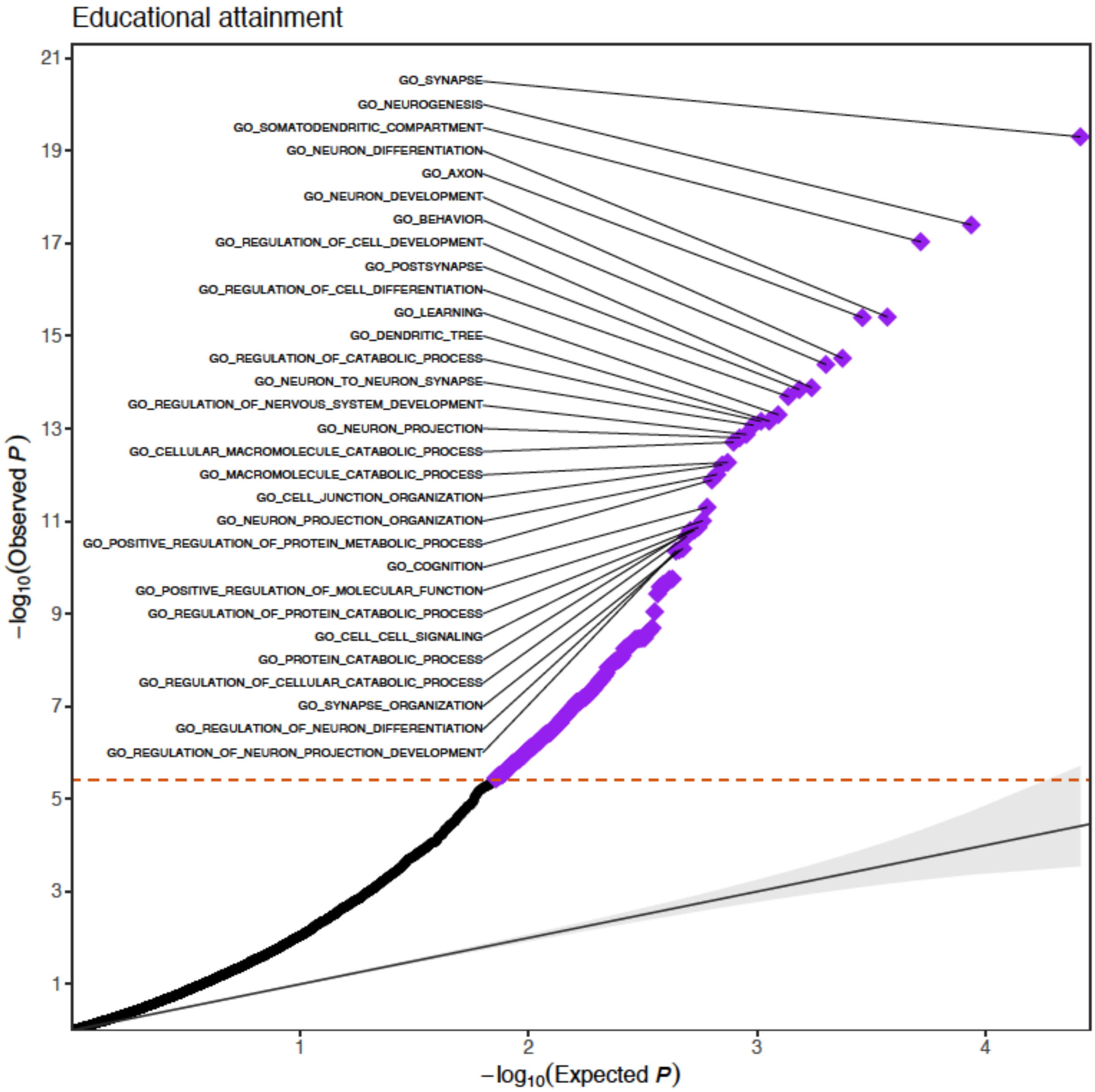
Educational attainment
GO_SYNAPSE
GO_NEUROGENESIS
GO_SOMATODENDRITIC_COMPARTMENT
GO_NEURON_DIFFERENTIATION
GO_AXON
GO_NEURON_DEVELOPMENT
GO_BEHAVIOR
GO_REGULATION_OF_CELL_DEVELOPMENT
GO_POSTSYNAPSE
GO_REGULATION_OF_CELL_DIFFERENTIATION
GO_LEARNING
GO_DENDRITIC_TREE
GO_REGULATION_OF_CATABOLIC_PROCESS
GO_NEURON_TO_NEURON_SYNAPSE
GO_REGULATION_OF_NERVOUS_SYSTEM_DEVELOPMENT
GO_NEURON_PROJECTION
GO_CELLULAR_MACROMOLECULE_CATABOLIC_PROCESS
GO_MACROMOLECULE_CATABOLIC_PROCESS
GO_CELL_JUNCTION_ORGANIZATION
GO_NEURON_PROJECTION_ORGANIZATION
GO_POSITIVE_REGULATION_OF_PROTEIN_METABOLIC_PROCESS
GO_COGNITION
GO_POSITIVE_REGULATION_OF_MOLECULAR_FUNCTION
GO_REGULATION_OF_PROTEIN_CATABOLIC_PROCESS
GO_CELL_CELL_SIGNALING
GO_PROTEIN_CATABOLIC_PROCESS
GO_REGULATION_OF_CELLULAR_CATABOLIC_PROCESS
GO_SYNAPSE_ORGANIZATION
GO_REGULATION_OF_NEURON_DIFFERENTIATION
GO_REGULATION_OF_NEURON_PROJECTION_DEVELOPMENT
−log10(Observed P)
−log10(Expected P)
21
19
17
15
13
11
9
7
5
3
1
1
2
3
4

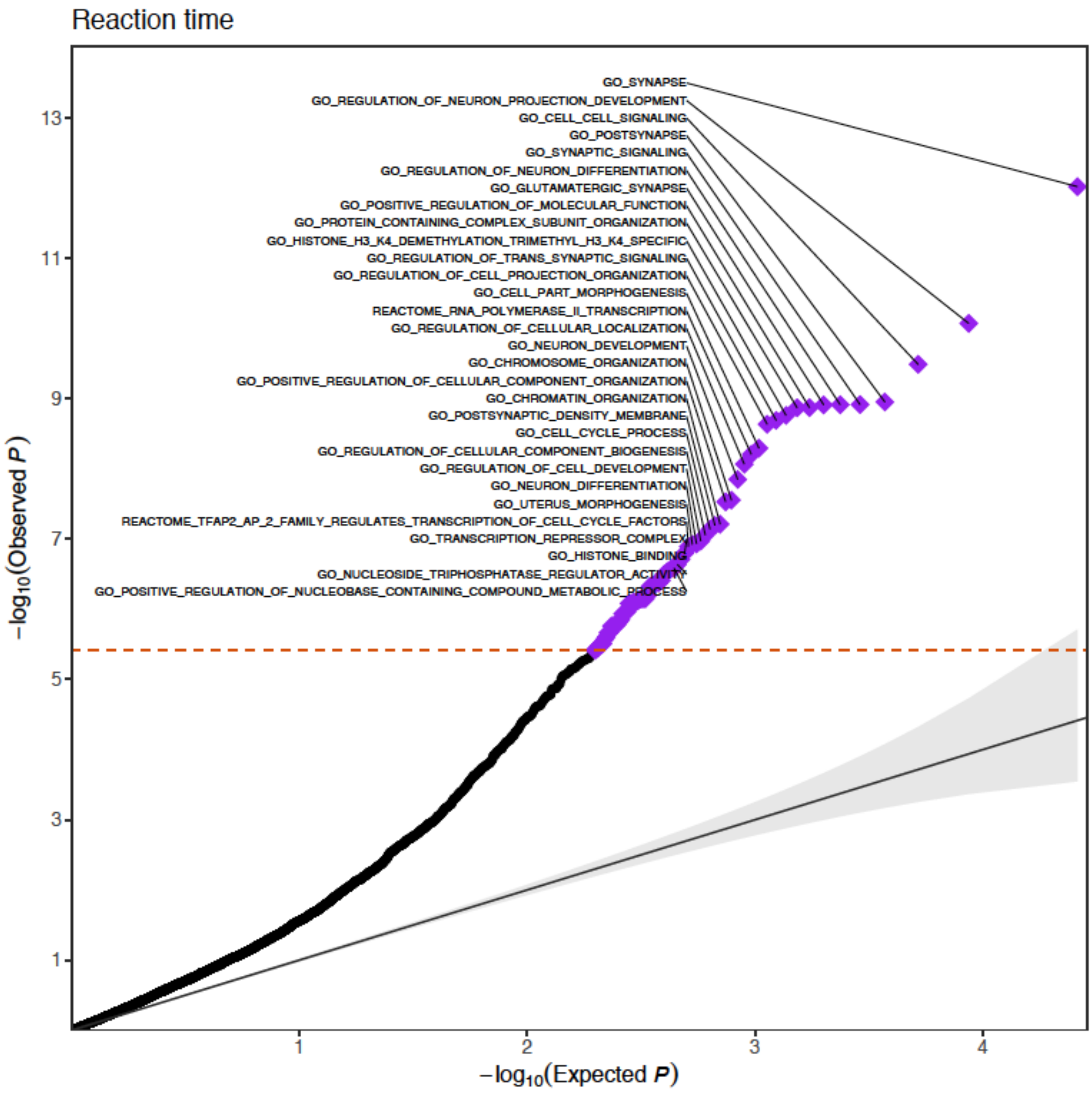

Reaction time
GO_SYNAPSE
GO_REGULATION_OF_NEURON_PROJECTION_DEVELOPMENT
GO_CELL_CELL_SIGNALING
GO_POSTSYNAPSE
GO_SYNAPTIC_SIGNALING
GO_REGULATION_OF_NEURON_DIFFERENTIATION
GO_GLUTAMATERGIC_SYNAPSE
GO_POSITIVE_REGULATION_OF_MOLECULAR_FUNCTION
GO_PROTEIN_CONTAINING_COMPLEX_SUBUNIT_ORGANIZATION
GO_HISTONE_H3_K4_DEMETHYLATION_TRIMETHYL_H3_K4_SPECIFIC
GO_REGULATION_OF_TRANS_SYNAPTIC_SIGNALING
GO_REGULATION_OF_CELL_PROJECTION_ORGANIZATION
GO_CELL_PART_MORPHOGENESIS
REACTOME_RNA_POLYMERASE_II_TRANSCRIPTION
GO_REGULATION_OF_CELLULAR_LOCALIZATION
GO_NEURON_DEVELOPMENT
GO_CHROMOSOME_ORGANIZATION
GO_POSITIVE_REGULATION_OF_CELLULAR_COMPONENT_ORGANIZATION
GO_CHROMATIN_ORGANIZATION
GO_POSTSYNAPTIC_DENSITY_MEMBRANE
GO_CELL_CYCLE_PROCESS
GO_REGULATION_OF_CELLULAR_COMPONENT_BIOGENESIS
GO_REGULATION_OF_CELL_DEVELOPMENT
GO_NEURON_DIFFERENTIATION
GO_UTERUS_MORPHOGENESIS
REACTOME_TFAP2_AP_2_FAMILY_REGULATES_TRANSCRIPTION_OF_CELL_CYCLE_FACTORS
GO_TRANSCRIPTION_REPRESSOR_COMPLEX
GO_HISTONE_BINDING
GO_NUCLEOSIDE_TRIPHOSPHATASE_REGULATOR_ACTIVITY
GO_POSITIVE_REGULATION_OF_NUCLEOBASE_CONTAINING_COMPOUND_METABOLIC_PROCESS
$-\log_{10}(\text{Observed } P)$
13
11
9
7
5
3
1
1
2
3
4
$-\log_{10}(\text{Expected } P)$

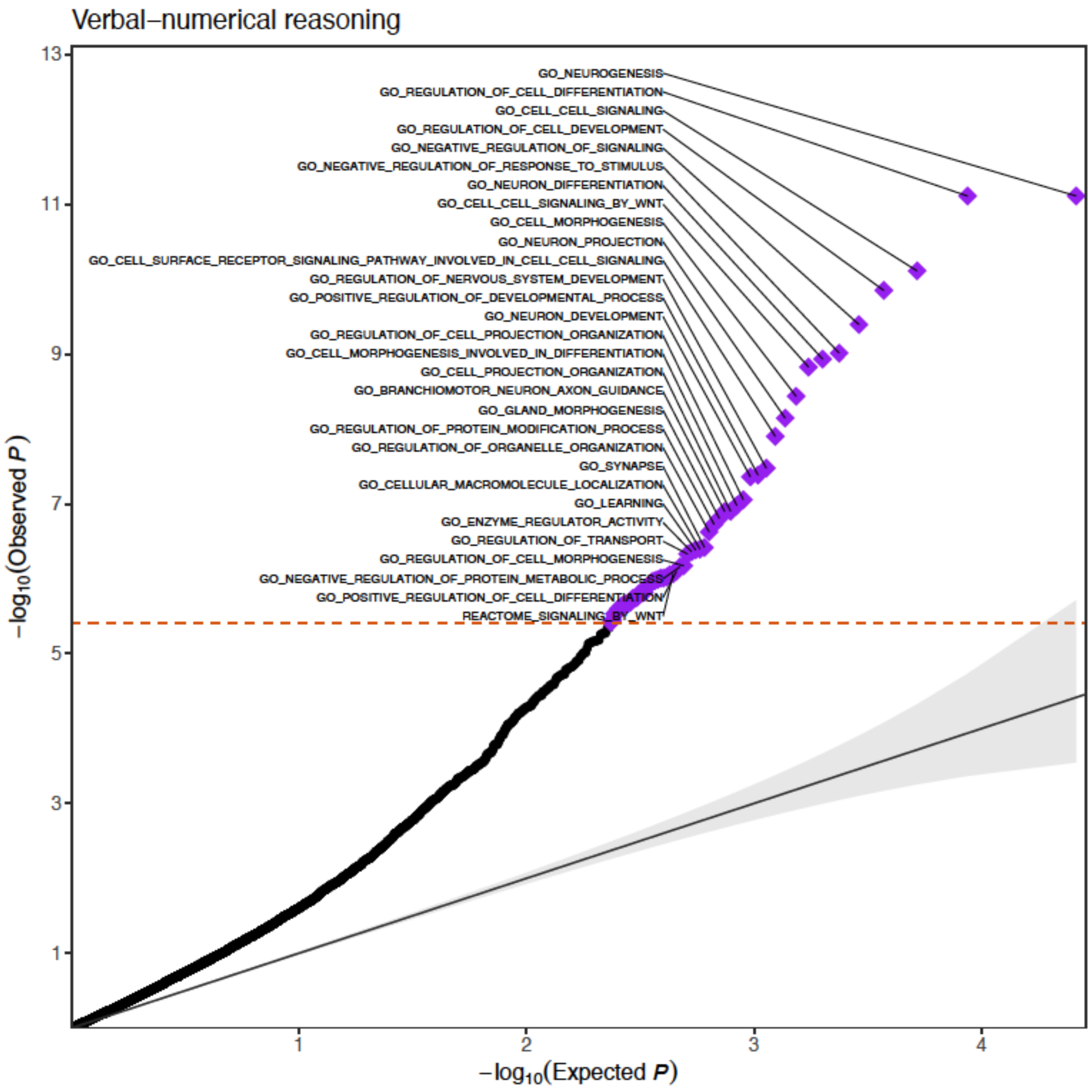

Supplementary Fig. 9. Gene set-based PTV burden analysis in European samples in the UK Biobank for educational attainment, Reaction time and verbal-numerical reasoning.
Top 30 gene sets were labeled in the figure. A total of 13,011 gene set from MSigDB v7.2 were identified, including C2 canonical pathways (N=2,808) and C5 Gene Ontology biological process (N=7,531), cellular component (N = 996), and molecular function (N=1,676). The gray band represents the 95% confidence interval. The gene set association p-values were based on two-sided *t*-tests from linear regression, adjusted for sex, age, age2, sex by age interaction, sex by age2 interaction, top 20 PCs, and recruitment centers (as categorical variables).

Reference

1. Bycroft, C. *et al.* The UK Biobank resource with deep phenotyping and genomic data. *Nature* **562**, 203–209 (2018).
2. Lee, J. J. *et al.* Gene discovery and polygenic prediction from a genome-wide association study of educational attainment in 1.1 million individuals. *Nat. Genet.* **50**, 1112–1121 (2018).
3. 1000 Genomes Project Consortium *et al.* A global reference for human genetic variation. *Nature* **526**, 68–74 (2015).
4. Backman, J. D. *et al.* Exome sequencing and analysis of 454,787 UK Biobank participants. *Nature* **599**, 628–634 (2021).
5. Savage, J. E. *et al.* Genome-wide association meta-analysis in 269,867 individuals identifies new genetic and functional links to intelligence. *Nat. Genet.* **50**, 912–919 (2018).
6. Davies, G. *et al.* Study of 300,486 individuals identifies 148 independent genetic loci influencing general cognitive function. *Nat. Commun.* **9**, 2098 (2018).
7. Lam, M. *et al.* Identifying nootropic drug targets via large-scale cognitive GWAS and transcriptomics. *Neuropsychopharmacology* **46**, 1788–1801 (2021).
8. Lee, S. *et al.* Optimal unified approach for rare-variant association testing with application to small-sample case-control whole-exome sequencing studies. *Am. J. Hum. Genet.* **91**, 224–237 (2012).
9. Wu, M. C. *et al.* Rare-variant association testing for sequencing data with the sequence kernel association test. *Am. J. Hum. Genet.* **89**, 82–93 (2011).
10. Karczewski, K. J. *et al.* Systematic single-variant and gene-based association testing of thousands of phenotypes in 394,841 UK Biobank exomes. *Cell Genomics* **2**, 100168 (2022).
11. Satterstrom, F. K. *et al.* Autism spectrum disorder and attention deficit hyperactivity disorder have a similar burden of rare protein-truncating variants. *Nat. Neurosci.* **22**, 1961–1965 (2019).
12. Kaplanis, J. *et al.* Evidence for 28 genetic disorders discovered by combining healthcare and research data. *Nature* **586**, 757–762 (2020).

13. Trubetskoy, V. *et al.* Mapping genomic loci implicates genes and synaptic biology in schizophrenia. *Nature* **604**, 502–508 (2022).
14. Stahl, E. A. *et al.* Genome-wide association study identifies 30 loci associated with bipolar disorder. *Nat. Genet.* **51**, 793–803 (2019).
15. Howard, D. M. *et al.* Genome-wide meta-analysis of depression identifies 102 independent variants and highlights the importance of the prefrontal brain regions. *Nat. Neurosci.* **22**, 343–352 (2019).
16. Watanabe, K., Taskesen, E., van Bochoven, A. & Posthuma, D. Functional mapping and annotation of genetic associations with FUMA. *Nat. Commun.* **8**, 1826 (2017).
17. Satterstrom, F. K. *et al.* Large-Scale Exome Sequencing Study Implicates Both Developmental and Functional Changes in the Neurobiology of Autism. *Cell* **180**, 568–584.e23 (2020).
18. de Leeuw, C. A., Neale, B. M., Heskes, T. & Posthuma, D. The statistical properties of gene-set analysis. *Nat. Rev. Genet.* **17**, 353–364 (2016).
19. Sjöstedt, E. *et al.* An atlas of the protein-coding genes in the human, pig, and mouse brain. *Science* **367**, eaay5947 (2020).
20. Kurki, M. I. *et al.* Contribution of rare and common variants to intellectual disability in a sub-isolate of Northern Finland. *Nat. Commun.* **10**, 410 (2019).
21. Karlson, E. W., Boutin, N. T., Hoffnagle, A. G. & Allen, N. L. Building the Partners HealthCare Biobank at Partners Personalized Medicine: Informed Consent, Return of Research Results, Recruitment Lessons and Operational Considerations. *J Pers Med* **6**, (2016).
22. McLaren, W. *et al.* The Ensembl Variant Effect Predictor. *Genome Biol.* **17**, 122 (2016).
23. Karczewski, K. J. *et al.* The mutational constraint spectrum quantified from variation in 141,456 humans. *Nature* **581**, 434–443 (2020).
24. Gapp, K. *et al.* Potential of Environmental Enrichment to Prevent Transgenerational Effects of Paternal Trauma. *Neuropsychopharmacology* **41**, 2749–2758 (2016).
25. Koopmans, G., Blokland, A., van Nieuwenhuijzen, P. & Prickaerts, J. Assessment of spatial learning abilities of mice in a new circular maze. *Physiol. Behav.* **79**, 683–693 (2003).

26. Cruz-Sanchez, A. *et al.* Developmental onset distinguishes three types of spontaneous recognition memory in mice. *Sci. Rep.* **10**, 10612 (2020).

27. Dobin, A. *et al.* STAR: ultrafast universal RNA-seq aligner. *Bioinformatics* **29**, 15–21 (2013).

28. Liao, Y., Smyth, G. K. & Shi, W. featureCounts: an efficient general purpose program for assigning sequence reads to genomic features. *Bioinformatics* **30**, 923–930 (2014).

29. Love, M. I., Huber, W. & Anders, S. Moderated estimation of fold change and dispersion for RNA-seq data with DESeq2. *Genome Biol.* **15**, 550 (2014).

30. Leek, J. T. & Storey, J. D. Capturing heterogeneity in gene expression studies by surrogate variable analysis. *PLoS Genet.* **3**, 1724–1735 (2007).

31. Kang, H. J. *et al.* Spatio-temporal transcriptome of the human brain. *Nature* **478**, 483–489 (2011).

32. Watkins, L. R. & Orlandi, C. Orphan G Protein Coupled Receptors in Affective Disorders. *Genes* **11**, 694 (2020).

33. Kee, H. J. *et al.* Expression of brain-specific angiogenesis inhibitor 2 (BAI2) in normal and ischemic brain: involvement of BAI2 in the ischemia-induced brain angiogenesis. *J. Cereb. Blood Flow Metab.* **22**, 1054–1067 (2002).

34. Fagerberg, L. *et al.* Analysis of the human tissue-specific expression by genome-wide integration of transcriptomics and antibody-based proteomics. *Mol. Cell. Proteomics* **13**, 397–406 (2014).

35. Sakaue, S. *et al.* A cross-population atlas of genetic associations for 220 human phenotypes. *Nat. Genet.* **53**, 1415–1424 (2021).

36. Khawaja, A. P. *et al.* Genome-wide analyses identify 68 new loci associated with intraocular pressure and improve risk prediction for primary open-angle glaucoma. *Nat. Genet.* **50**, 778–782 (2018).

37. Wright, K. M. *et al.* A Prospective Analysis of Genetic Variants Associated with Human Lifespan. *G3* **9**, 2863–2878 (2019).

38. Vallianatos, C. N. & Iwase, S. Disrupted intricacy of histone H3K4 methylation in neurodevelopmental disorders. *Epigenomics* **7**, 503–519 (2015).

39. Han, M., Xu, W., Cheng, P., Jin, H. & Wang, X. Histone demethylase lysine demethylase 5B in development and cancer. *Oncotarget* **8**, 8980–8991 (2017).

40. Xhabija, B. & Kidder, B. L. KDM5B is a master regulator of the H3K4-methylome in stem cells, development and cancer. *Semin. Cancer Biol.* **57**, 79–85 (2019).

41. Najmabadi, H. *et al.* Deep sequencing reveals 50 novel genes for recessive cognitive disorders. *Nature* **478**, 57–63 (2011).

42. Takata, A. *et al.* Loss-of-function variants in schizophrenia risk and SETD1A as a candidate susceptibility gene. *Neuron* **82**, 773–780 (2014).

43. De Rubeis, S. *et al.* Synaptic, transcriptional and chromatin genes disrupted in autism. *Nature* **515**, 209–215 (2014).

44. Iossifov, I. *et al.* The contribution of de novo coding mutations to autism spectrum disorder. *Nature* **515**, 216–221 (2014).

45. Firth, J. *et al.* Grip Strength Is Associated With Cognitive Performance in Schizophrenia and the General Population: A UK Biobank Study of 476559 Participants. *Schizophr. Bull.* **44**, 728–736 (2018).

46. Giovannone, B. *et al.* Two novel proteins that are linked to insulin-like growth factor (IGF-I) receptors by the Grb10 adapter and modulate IGF-I signaling. *J. Biol. Chem.* **278**, 31564–31573 (2003).

47. Dufresne, A. M. & Smith, R. J. The adapter protein GRB10 is an endogenous negative regulator of insulin-like growth factor signaling. *Endocrinology* **146**, 4399–4409 (2005).

48. Astle, W. J. *et al.* The Allelic Landscape of Human Blood Cell Trait Variation and Links to Common Complex Disease. *Cell* **167**, 1415–1429.e19 (2016).

49. Sinnott-Armstrong, N. *et al.* Genetics of 35 blood and urine biomarkers in the UK Biobank. *Nat. Genet.* **53**, 185–194 (2021).

50. Zhao, Y. *et al.* GIGYF1 loss of function is associated with clonal mosaicism and adverse metabolic health. *Nat. Commun.* **12**, 4178 (2021).

51. Deaton, A. M. *et al.* Gene-level analysis of rare variants in 379,066 whole exome sequences identifies an association of GIGYF1 loss of function with type 2 diabetes. *Sci. Rep.* **11**, 21565 (2021).

52. Li, C.-W., Dinh, G. K., Zhang, A. & Chen, J. D. Ankyrin repeats-containing cofactors interact with ADA3 and modulate its co-activator function. *Biochem. J* **413**, 349–357 (2008).

53. Zhang, A. *et al.* Identification of a novel family of ankyrin repeats containing cofactors for p160 nuclear receptor coactivators. *J. Biol. Chem.* **279**, 33799–33805 (2004).

54. Chen, M.-H. *et al.* Trans-ethnic and Ancestry-Specific Blood-Cell Genetics in 746,667 Individuals from 5 Global Populations. *Cell* **182**, 1198–1213.e14 (2020).

55. Shieh, B. H. *et al.* Mapping of the gene for the cardiac sarcolemmal Na(+)-Ca2+ exchanger to human chromosome 2p21-p23. *Genomics* **12**, 616–617 (1992).

56. Morris, J. A. *et al.* An atlas of genetic influences on osteoporosis in humans and mice. *Nat. Genet.* **51**, 258–266 (2019).

57. Giri, A. *et al.* Trans-ethnic association study of blood pressure determinants in over 750,000 individuals. *Nat. Genet.* **51**, 51–62 (2019).

58. Ntalla, I. *et al.* Multi-ancestry GWAS of the electrocardiographic PR interval identifies 202 loci underlying cardiac conduction. *Nat. Commun.* **11**, 2542 (2020).

59. Arking, D. E. *et al.* Genetic association study of QT interval highlights role for calcium signaling pathways in myocardial repolarization. *Nat. Genet.* **46**, 826–836 (2014).

60. Willems, S. M. *et al.* Large-scale GWAS identifies multiple loci for hand grip strength providing biological insights into muscular fitness. *Nat. Commun.* **8**, 16015 (2017).

61. Siess, D. C. *et al.* A human gene coding for a membrane-associated nucleic acid-binding protein. *J. Biol. Chem.* **275**, 33655–33662 (2000).

62. Vinuesa, C. G. *et al.* A RING-type ubiquitin ligase family member required to repress follicular helper T cells and autoimmunity. *Nature* **435**, 452–458 (2005).

63. Zhang, Q. *et al.* New Insights into the RNA-Binding and E3 Ubiquitin Ligase Activities of Roquins.

Sci. Rep. **5**, 15660 (2015).

64. Heissmeyer, V. & Vogel, K. U. Molecular control of Tfh-cell differentiation by Roquin family proteins. *Immunol. Rev.* **253**, 273–289 (2013).

65. Vogel, K. U. *et al.* Roquin paralogs 1 and 2 redundantly repress the Icos and Ox40 costimulator mRNAs and control follicular helper T cell differentiation. *Immunity* **38**, 655–668 (2013).

66. Jansen, P. R. *et al.* Genome-wide analysis of insomnia in 1,331,010 individuals identifies new risk loci and functional pathways. *Nat. Genet.* **51**, 394–403 (2019).

67. Baselmans, B. M. L. *et al.* Multivariate genome-wide analyses of the well-being spectrum. *Nat. Genet.* **51**, 445–451 (2019).

68. Mills, M. C. *et al.* Identification of 371 genetic variants for age at first sex and birth linked to externalising behaviour. *Nat Hum Behav* **5**, 1717–1730 (2021).

69. Zhao, B. *et al.* Genome-wide association analysis of 19,629 individuals identifies variants influencing regional brain volumes and refines their genetic co-architecture with cognitive and mental health traits. *Nat. Genet.* **51**, 1637–1644 (2019).

70. Siva, K., Venu, P., Mahadevan, A., S K, S. & Inamdar, M. S. Human BCAS3 expression in embryonic stem cells and vascular precursors suggests a role in human embryogenesis and tumor angiogenesis. *PLoS One* **2**, e1202 (2007).

71. Hengel, H. *et al.* Bi-allelic loss-of-function variants in BCAS3 cause a syndromic neurodevelopmental disorder. *Am. J. Hum. Genet.* **108**, 1069–1082 (2021).

72. Wuttke, M. *et al.* A catalog of genetic loci associated with kidney function from analyses of a million individuals. *Nat. Genet.* **51**, 957–972 (2019).

73. Tin, A. *et al.* Target genes, variants, tissues and transcriptional pathways influencing human serum urate levels. *Nat. Genet.* **51**, 1459–1474 (2019).

74. Gao, X. R., Huang, H. & Kim, H. Genome-wide association analyses identify 139 loci associated with macular thickness in the UK Biobank cohort. *Hum. Mol. Genet.* **28**, 1162–1172 (2019).

75. Koyama, S. *et al.* Population-specific and trans-ancestry genome-wide analyses identify distinct and

shared genetic risk loci for coronary artery disease. *Nat. Genet.* **52**, 1169–1177 (2020).

76. Weiner, D. J. *et al.* Polygenic architecture of rare coding variation across 394,783 exomes. *Nature* **614**, 492–499 (2023).

77. O'Connor, L. J. *et al.* Extreme Polygenicity of Complex Traits Is Explained by Negative Selection. *Am. J. Hum. Genet.* **105**, 456–476 (2019).
