# Supplementary material for: The impact of rare protein coding genetic variation on adult cognitive function
Source: Nat Genet. 2023 May 25;55(6):927–38. doi: 10.1038/s41588-023-01398-8 (PMC10260403; doi:10.1038/s41588-023-01398-8)
Supplement: Supplementary file 2 — Reporting Summary [file 41588_2023_1398_MOESM2_ESM.pdf]

nature portfolio

Corresponding author(s): Chia-Yen Chen, Heiko Runz

Last updated by author(s): Apr 12, 2023

Reporting Summary

Nature Portfolio wishes to improve the reproducibility of the work that we publish. This form provides structure for consistency and transparency in reporting. For further information on Nature Portfolio policies, see our Editorial Policies and the Editorial Policy Checklist.

Statistics

For all statistical analyses, confirm that the following items are present in the figure legend, table legend, main text, or Methods section.

n/a	Confirmed	
☐	☒	The exact sample size (*n*) for each experimental group/condition, given as a discrete number and unit of measurement
☐	☒	A statement on whether measurements were taken from distinct samples or whether the same sample was measured repeatedly
☐	☒	The statistical test(s) used AND whether they are one- or two-sided<br>*Only common tests should be described solely by name; describe more complex techniques in the Methods section.*
☐	☒	A description of all covariates tested
☐	☒	A description of any assumptions or corrections, such as tests of normality and adjustment for multiple comparisons
☐	☒	A full description of the statistical parameters including central tendency (e.g. means) or other basic estimates (e.g. regression coefficient) AND variation (e.g. standard deviation) or associated estimates of uncertainty (e.g. confidence intervals)
☐	☒	For null hypothesis testing, the test statistic (e.g. *F*, *t*, *r*) with confidence intervals, effect sizes, degrees of freedom and *P* value noted<br>*Give P values as exact values whenever suitable.*
☐	☒	For Bayesian analysis, information on the choice of priors and Markov chain Monte Carlo settings
☒	☐	For hierarchical and complex designs, identification of the appropriate level for tests and full reporting of outcomes
☐	☒	Estimates of effect sizes (e.g. Cohen's *d*, Pearson's *r*), indicating how they were calculated

Our web collection on statistics for biologists contains articles on many of the points above.

Software and code

Policy information about availability of computer code

Data collection	No specific software were employed in collection.
Data analysis	The code used in this study can be found at Zenodo (https://doi.org/10.5281/zenodo.7822074). Software used include R v3.6.1 and v4.1.3 (packages gprofiler2 v0.2.1, MASS v7.3-55, dglm v1.8.5, data.table vl.12.8, dplyr vl.0.0, ggplot2 v3.3.l, GSA vl.03.l); PRS-CS v1.0.0: https://github.com/getian107/PRScs; PLINK v2.00: https://www.cog-genomics.org/plink/2.0; PLINK vl.90: https://www.cog-genomics.org/plink/; VEP v96: https://useast.ensembl.org/info/docs/tools/vep/index.html; LOFTEE: https://github.com/konradjk/loftee; Regenie v1.0.6.7: https://rgcgithub.github.io/regenie/; Hail 0.2: https://github.com/hail-is/hail; LocusZoom v0.14.0: https://my.locuszoom.org/; FUMA v1.3.7: https://fuma.ctglab.nl/; FastQC v0.11.8: https://github.com/s-andrews/FastQC ; STAR version 2.7.3a: https://code.google.com/archive/p/rna-star/; featureCounts version 2.0.0: https://subread.sourceforge.net/; DESEQ2 v1.34.0: http://www.bioconductor.org/packages/release/bioc/html/DESeq2.html. Details of specific software and references can be found within text in the relevant Methods and Supplementary Methods sections.

For manuscripts utilizing custom algorithms or software that are central to the research but not yet described in published literature, software must be made available to editors and reviewers. We strongly encourage code deposition in a community repository (e.g. GitHub). See the Nature Portfolio guidelines for submitting code & software for further information.

Data

Policy information about availability of data

All manuscripts must include a data availability statement. This statement should provide the following information, where applicable:

- Accession codes, unique identifiers, or web links for publicly available datasets
- A description of any restrictions on data availability
- For clinical datasets or third party data, please ensure that the statement adheres to our policy

Full summary PTV burden association results derived from UK Biobank in this study can be found in Supplementary Table 4. For instructions on how to access the UK Biobank exome sequencing data, see https://www.ukbiobank.ac.uk/enable-your-research/research-analysis-platform. For the SUPER-Finland study, individual level genotype and diagnosis data is available through the THL biobank https://thl.fi/en/web/thl-biobank/for-researchers/sample-collections/super-study. For the Northern Finland Intellectual Disability (NFID) study, due to consent and EU privacy regulations (GDPR), individual level data can be used for research defined in the consent. Upon reasonable requests, aggregate level data can be requested from the Institute of Molecular Medicine, University of Helsinki (FIMM) data access committee (DAC; fimm-dac@helsinki.fi) or individual level data can be utilized for collaborative research given that it is within the scope of the consent and the individual level data is handled in a dedicated computational environment designated by the FIMM. Mass General Brigham Biobank data are not publicly available due to privacy and ethnical restrictions. Please contact MGB Biobank for further information on data access (https://www.massgeneralbrigham.org/en/research-and-innovation/participate-in-research/biobank/for-researchers). All Kdm5b mouse RNA-seq sequences (GRCm38) can be found in the European Nucleotide Archive (https://www.ebi.ac.uk/ena/browser/home) with accession numbers listed in Supplementary Table 17. pLI score is available at https://storage.googleapis.com/gcp-public-data--gnomad/release/2.1.1/constraint/gnomad.v2.1.1.lof_metrics.by_gene.txt.bgz. MPC score is available at ftp://ftp.broadinstitute.org/pub/ExAC_release/release1/regional_missense_constraint/ (open access ftp site; no registration required). Brainspan RNA sequencing data is available at https://www.brainspan.org/static/download.html. Human protein atlas data are available at https://www.proteinatlas.org/humanproteome/brain/human+brain. The Development Disorder Genotype - Phenotype Database (DDG2P) gene list is available at https://www.deciphergenomics.org/ddd/ddgenes. Social Science Genetic Association Consortium (SSGAC) educational attainment GWAS summary statistics are available at https://thessgac.com/ (registration required) and the educational attainment GWAS summary statistics file used in this study is at https://thessgac.com/papers/3/12 (accessible after registration)

Human research participants

Policy information about studies involving human research participants and Sex and Gender in Research.

Reporting on sex and gender	To minimize potential confounding, we adjusted for sex in all association analysis as a binary covariate. Sex information was collected by self-report in each respective study.
Population characteristics	The UK Biobank is a prospective cohort study of the UK population with over 500,000 participants. Participants were aged between 40 to 69 years at recruitment in 2006-2010 and provided extensive phenotype data, including surveys on baseline characteristics and health outcomes, specific questionnaires and assessments, health records, physical measures and biomarkers. A total of 454,787 whole-exome sequenced UK Biobank participants were included in this study.<br><br>The SUPER-Finland study is a cohort of 9,125 psychotic patients in Finland. Subjects with a diagnosis of a schizophrenia spectrum psychotic disorder, bipolar I disorder or major depressive disorder with psychotic features were recruited from in and outpatient psychiatric, general care and housing units and by advertisements in local newspapers. DNA samples were genotyped with GWAS arrays, exome sequenced and linked to a wide range of phenotypic information ascertained through a structured interview, questionnaires, and cognitive testing.<br><br>The Northern Finland Intellectual Disability (NFID) study includes 1,097 intellectual disability cases. We included 11,774 controls from the FIN RISK 1992-2012 and Health 2000-2011 studies. The details of the study sample recruitment and phenotyping, exome sequencing data generation and quality control and ethical permissions were described in Kurki et al. 2019.<br><br>The Mass General Brigham Biobank is a hospital-based biobank aiming at collecting blood samples, lifestyle and family history survey data, as well as electronic health record linkage from consented participants. The release used for this study (as of November 2021) includes 24,787 samples that were whole-exome sequenced and genome-wide genotyped.
Recruitment	The UK Biobank recruitment was done by participants volunteered to enter the study. The potential healthy volunteer bias in UK Biobank was investigated (Fry et al. 2017) and it was suggested that UK Biobank participants were less likely to smoke and drink alcohol daily, less likely to be obese, and had fewer self-reported health conditions, compared with the general population of UK. However, the healthy volunteer bias has minimal effects on the genetic association we investigated in this study.<br><br>Psychotic patients in the SUPER-Finland study were recruited from in- and outpatient psychiatric, general care and housing units and by advertisements in local newspapers. There are no known biases related to the sample recruitment.<br><br>For the Northern Finland Intellectual Disability (NFID) study, the intellectual disability cases were recruited all pediatric neurology units and centers for intellectual disability care in the special responsibility area of Oulu University Hospital. Cases were first identified through hospital records and then invited via mail. Additional recruitment during routine clinical visits were also done at the participating pediatric neurology units and centers. The controls were from FIN RISK study. FIN RISK is population-based cohort where health surveys on chronic diseases were carried out every 5 years since 1972. There are no known biases related to the sample recruitment in the NFID study and FINRISK study.<br><br>The Mass General Brigham Biobank is a hospital-based biobank where participants volunteered to enter the study. There are

	no known biases related to the sample recruitment.
Ethics oversight	UK Biobank is approved by the North West Multi-centre Research Ethics Committee (MREC; https://www.ukbiobank.ac.uk/learn-more-about-uk-biobank/about-us/ethics). The current study was conducted under the UK Biobank application number 26041. The data in UK Biobank were collected following informed consents obtained from all participants. The Human Research Committee of MGB approved the Biobank research protocol (2009P002312)71. The data in MGB Biobank were collected following a broad-based consent obtained from all participants. The Coordinating Ethical Committee of the Helsinki and Uusimaa Hospital Region approved the SUPER-Finland Study 16 July 2015 (pilot) and 9 February 2016 (full study). All participants of the SUPER-Finland Study signed an informed consent that permits the research use of collected samples and data. The ethical committees of the Northern Ostrobothnia Hospital District and the Hospital District of Helsinki and Uusimaa approved the NFID study. All participants and/or their legal guardians provided a written informed consent to the study.

Note that full information on the approval of the study protocol must also be provided in the manuscript.

Field-specific reporting

Please select the one below that is the best fit for your research. If you are not sure, read the appropriate sections before making your selection.

☒ Life sciences ☐ Behavioural & social sciences ☐ Ecological, evolutionary & environmental sciences

For a reference copy of the document with all sections, see nature.com/documents/nr-reporting-summary-flat.pdf

Life sciences study design

All studies must disclose on these points even when the disclosure is negative.

Sample size	See Methods for detailed descriptions on sample sizes for UK Biobank and other replication studies. Sample size were chosen to maximize the power for gene discovery from the largest available population cohorts with relevant cognitive function phenotypes, covariates, and whole-exome sequencing data. Note that we did not calculate power for detecting pre-specified effect sizes, as this study is aiming at gene discovery through association tests rather than estimating specific effect sizes.
Data exclusions	See Methods for data quality control details. Overall, samples failed quality control or with missing information were excluded from the analysis.
Replication	We performed replication in three independent studies for the findings from the discovery analysis in UK Biobank European samples, including the SUPER-Finland study, the Northern Finland Intellectual Disability (NFID) study, and Mass General Brigham Biobank. Our replication analyses validated that LoF in the cognitive function genes identified in UK Biobank reduces adult cognitive function.
Randomization	Randomization is not applicable as this is an observational study.
Blinding	Blinding is not applicable as this is an observational study.

Reporting for specific materials, systems and methods

We require information from authors about some types of materials, experimental systems and methods used in many studies. Here, indicate whether each material, system or method listed is relevant to your study. If you are not sure if a list item applies to your research, read the appropriate section before selecting a response.

Materials & experimental systems

n/a	Involved in the study	
☒	☐	Antibodies
☒	☐	Eukaryotic cell lines
☒	☐	Palaeontology and archaeology
☐	☒	Animals and other organisms
☒	☐	Clinical data
☒	☐	Dual use research of concern

Methods

n/a	Involved in the study	
☒	☐	ChIP-seq
☒	☐	Flow cytometry
☒	☐	MRI-based neuroimaging

Animals and other research organisms

Policy information about studies involving animals; ARRIVE guidelines recommended for reporting animal research, and Sex and Gender in Research

Laboratory animals	A mouse Kdm5b loss of function allele (MGI:6153378) was generated previously by CRISPR/CAS9 mediated deletion of coding exon 7 (ENSMUSE00001331577), leading to a premature translational termination due to a downstream frameshift. Breeding of testing cohorts was performed on a C57BL/6NJ background. The testing cohort with C57BL/6NJ strain background (see https://www.jax.org/

	strain/005304 for details). at 10-16 weeks of age adult mice, and E18.5 for embryonic mice brain. Mice were housed in specific pathogen-free mouse facilities with 12 hours light/dark cycle (lights on at 07:30 am), ambient temperature of 21 degree Celsius and humidity of 55%. Food and water were available ad libitum.
Wild animals	This study did not involve wild animals.
Reporting on sex	Our goal with this experiment is to determine the gene dosage effects on behavioral, cognitive, and molecular phenotypes in mice across sex and therefore sex information was not use in the analysis of the mouse data and no sex-specific analysis was done.
Field-collected samples	This study did not involve animals collected in the field.
Ethics oversight	The breeding, housing of mice and all procedures for the Kdm5b loss of function mouse experiments were assessed by the Animal Welfare and Ethical Review Body of the Wellcome Sanger Institute and conducted under the regulation of UK Home Office license (P6320B89B), and in accordance with institutional guidelines.

Note that full information on the approval of the study protocol must also be provided in the manuscript.
